# Spatially Informed Nonnegative Matrix Trifactorization for Coclustering Mass Spectrometry Data

**DOI:** 10.1002/bimj.70031

**Published:** 2025-03-19

**Authors:** Andrea Sottosanti, Francesco Denti, Stefania Galimberti, Davide Risso, Giulia Capitoli

**Affiliations:** ^1^ Department of Medicine University of Padova Padova Italy; ^2^ Department of Statistical Sciences University of Padova Padova Italy; ^3^ Bicocca Bioinformatics Biostatistics and Bioimaging B4 Center School of Medicine and Surgery University of Milano‐Bicocca Monza Italy; ^4^ Biostatistics and Clinical Epidemiology Fondazione IRCCS San Gerardo dei Tintori Monza Italy

**Keywords:** brain imaging, coclustering, MALDI‐MSI, matrix trifactorization, spatial proteomics

## Abstract

Mass spectrometry imaging techniques measure molecular abundance in a tissue sample at a cellular resolution, all while preserving the spatial structure of the tissue. This kind of technology offers a detailed understanding of the role of several molecular factors in biological systems. For this reason, the development of fast and efficient computational methods that can extract relevant signals from massive experiments has become necessary. A key goal in mass spectrometry data analysis is the identification of molecules with similar functions in the analyzed biological system. This result can be achieved by studying the spatial distribution of the molecules' abundance patterns. To do so, one can perform coclustering, that is, dividing the molecules into groups according to their expression patterns over the tissue and segmenting the tissue according to the molecules' abundance levels. We present TRIFASE, a semi‐nonnegative matrix trifactorization technique that performs coclustering while accounting for the spatial correlation of the data. We propose an estimation algorithm that solves the proposed matrix trifactorization problem. Moreover, to improve scalability, we also propose two heuristic approximations of the most expensive steps, which help the algorithm converge while significantly streamlining the computational cost. We validated our method on a series of simulation experiments, comparing the different estimating strategies discussed in the article. Last, we analyzed a mouse brain tissue sample processed with MALDI‐MSI technology, showing how TRIFASE extracts specific expression patterns of molecule abundance in localized tissue areas and discovers blocks of proteins whose activation is directly linked to specific biological mechanisms.

## 1 Introduction

### 1.1 Motivating Data Set and Need for Coclustering From a Biological Perspective

Biological systems are inherently intricate, and many molecular factors influence their functions. Recent advancements in molecular approaches, such as mass spectrometry, have greatly improved the in‐depth exploration of tissue biopsy. In particular, in fields like clinical pathology, there has been a swift acceleration in the expansion of single‐cell omics technologies, offering the potential for invaluable insights into human diseases.

Among all the techniques, matrix‐assisted laser desorption/ionization mass spectrometry imaging (MALDI‐MSI) is a technology that captures and measures various biomolecules within their native spatial context at a spatial resolution up to the single‐cell level. Indeed, the combination of the information deriving from the spectra with their spatial references sheds new light in the field of cytopathology, as it permits the detection of sparsely dispersed abnormal cells or intricate dynamics present in the tumor microenvironment. These characteristics are usually overseen by traditional methodologies (Schober et al. 2012). More precisely, MALDI‐MSI can capture the distribution of different molecular information, such as lipids, tryptic peptides, and N‐glycans, allowing the highlight of a complete picture of these systems (Rohner, Staab, and Stoeckli 2005; Denti et al. 2022). By combining molecular data with morphological information, such as tissue structure, it is possible to gain a more comprehensive understanding of the molecules associated with different biological functions of the tissue sample. Thus, MALDI‐MSI enhances value in scenarios where clinical tissue samples are limited, such as in the study of rare diseases or when multiple histological and immunohistochemical assessments are required. The availability of these complex measurements has paved the way for the development of statistical methods designed to capture these molecular landscapes.

**FIGURE 1:**
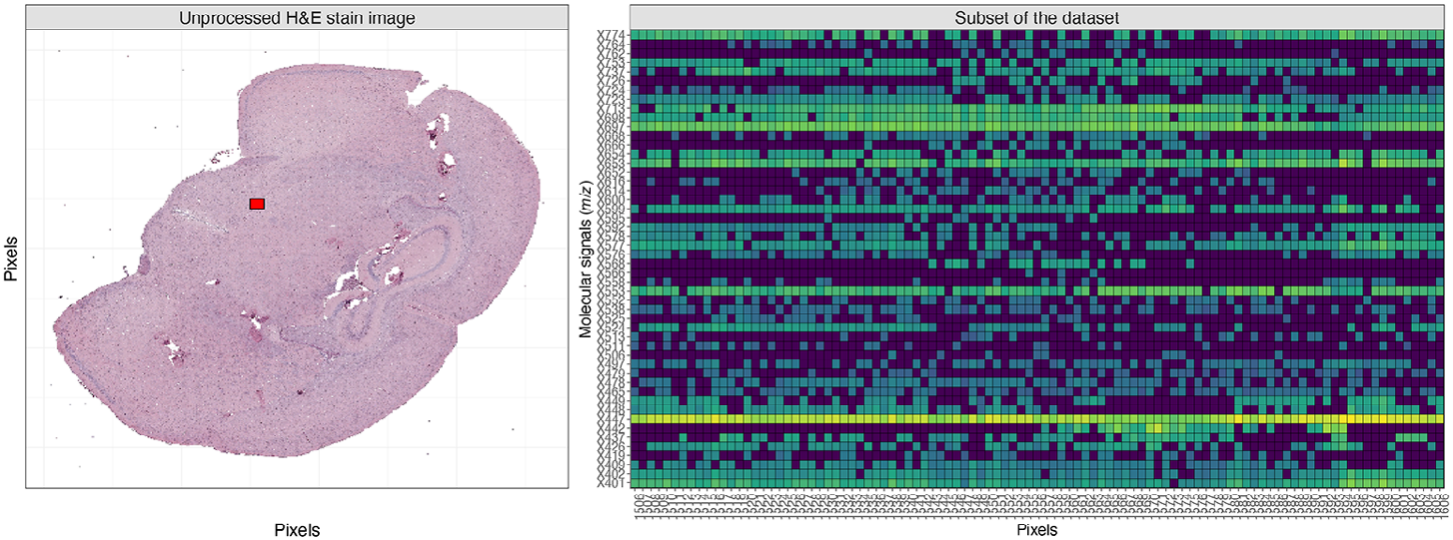
Left panel: unprocessed hematoxylin and eosin (H&E) stain representation of the analyzed brain tissue obtained from a healthy mouse. Right panel: heatmap displaying the structure of a subset of the original data matrix we analyze.

In this work, we consider the MALDI‐MSI analysis of a single section of a healthy mouse brain tissue; in particular, we investigate the mass spectra of lipids. The slice from the mouse brain, depicted in the left panel of Figure 1, is partitioned into a grid of pixels, creating a raster of cells of 50 μm each. Then, MALDI‐MSI acquires a mass spectrum for each pixel. Each spectrum measures the observed abundance for every mass‐to‐charge (m/z) signal value, representing analytes of interest (Boggio et al. 2011). Due to experimental limitations, the MALDI‐MSI spectra might contain noise affecting the statistical analysis. Indeed, the raw data set undergoes some initial preprocessing steps to filter out the noise. In addition, the preprocessing needs to reduce the intrasample variability and correct for analytical and instrumental variability following sample preparation to ensure accurate m/z localization. A detailed description of this well‐established preprocessing pipeline is beyond the scope of this article, but the interested reader may refer to Smith et al. (2021) for more details. The preprocessed data, measuring the abundances of lipids across the image, are organized into a matrix. The matrix columns correspond to approximately 6500 locations. Indeed, each column is accompanied by its pixel coordinates (x,y) in the original image and represents the extracted mass spectrum in a specific location, measuring the abundance of approximately 100 m/z signals. Therefore, the matrix rows contain the m/z values. In this work, the MALDI‐MSI analysis extracted the information regarding lipid molecules. In the right panel of Figure 1, we display the heatmap of a subset of the original matrix, corresponding to the red area highlighted over the brain slice.

The nontrivial structure of the measurements resulting from this experiment calls for the development of tailored methods that can adequately consider the complexity of the dependencies among the data. In this work, we focus on developing a fast coclustering algorithm, intending to simultaneously estimate a two‐dimensional partition, one across the rows and one across the columns. The latter, in particular, would allow the recovery of image segmentation solutions. In other words, we aim to obtain clusters of rows and columns to detect brain subregions where a certain subset of signals is significantly over‐ or underexpressed.

The statistical literature that focuses on coclustering problems largely concentrates on the latent block model, which is an extension of the mixture model for performing simultaneous clustering of the two dimensions of a matrix. Several versions of this model for the analysis of multiple types of data, such as continuous data, binary data, and contingency tables, have been developed both within the frequentist and the Bayesian paradigms (see, e.g., Govaert and Nadif 2013; Bouveyron et al. 2019). Beyond traditional statistical approaches, nonnegative matrix trifactorization (NMTF) methods offer an alternative and computationally efficient avenue for coclustering. NMTF seeks to approximate an input matrix with the product of three low‐rank matrices. Depending on the constraints applied to these matrices, NMTF can be employed for diverse purposes, such as pattern recognition (Zitnik and Zupan 2016; C̆opar, žitnik, and Zupan 2017), image segmentation (Gong et al. 2013; Hanmandlu et al. 2013), and clustering (Ding et al. 2006; Wang et al. 2011; Wang and Huang 2017; C̆opar, Zupan, and Zitnik 2019). The constantly evolving literature on matrix trifactorization algorithms ensures that new and efficient solutions are continuously proposed to cope with massive data sets.

### 1.2 Main Contributions and Outline

In this work, we propose the *TRIFactorization Algorithm of Spatial Expressions* (TRIFASE), a matrix trifactorization procedure designed for performing coclustering in a framework where the columns (or rows) of the data exhibit some form of dependence. We specifically focus on spatial correlation, thus when each data column (or row) is observed at a particular point in space, like the MALDI‐MSI data.

In recent years, several clustering techniques that embed the spatial structure of the data into the model have appeared in the literature. While Zhang et al. (2016) primarily focused on image analysis, two notable examples of clustering methods for the analysis of biological tissues processed with spatial transcriptomic technologies are BayesSpace (Zhao et al. 2021), which models the spatial data structure through the Potts model, and GraphST (Long et al. 2023), which is based on graph neural networks. A notable clustering method for spatial proteomic data was proposed by Fernsel (2021), utilizing a matrix factorization algorithm based on total variation regularization, illustrating its application in the classification of areas in a mouse brain tissue sample. The reader can refer to Prasad et al. (2022) for a thorough survey of methods for determining spatial features and clusters in MALDI‐MSI data, and to Leng et al. (2017) for the first example of total variation regularization employing nonnegative matrix factorization for a clustering problem. All these methods represent significant advancements in the analysis and clustering of data observed on a spatial domain, but they cannot perform simultaneous clustering on both the observations and the features of a data set.

Moving to the coclustering framework, most of the algorithms based on trifactorization proposed in the literature approach the coclustering problem by imposing nonnegative or orthogonal constraints on the three low‐rank matrices employed in the trifactorization (Ding et al. 2006; Ding, Li, and Jordan 2010; Pompili et al. 2014; Wang and Huang 2017; C̆opar, Zupan, and Zitnik 2019). Instead, our algorithm directly collects the membership labels into two of the three matrices, delivering a direct interpretation of the results and the discovered groups, as originally proposed in Wang et al. (2011). In the literature of trifactorization methods, there are only a few attempts to consider generic forms of data dependency, which is always expressed in the form of graphs (Gu and Zhou 2009; Wang et al. 2011; Shang, Jiao, and Wang 2012). The recent coclustering algorithm by Deng et al. (2021) regulates the model parameter estimates using both $L_{1}$ and $L_{2}$ penalizations and embeds the connection structure of observations and features. However, they assume all three matrices involved in the trifactorization are positive, thus requiring a further postprocessing step to obtain the coclusters. To the best of our knowledge, no solution based on matrix trifactorization specifically focuses on embedding the spatial covariance of the data, expressed as a function of the distances of the points, into the minimization problem. We remark that, albeit inspired by research on trifactorization methods applied to nonnegative matrices, our method works for real‐valued matrix data. The literature refers to this case as *semi ‐NMTF* (Ding, Li, and Jordan 2010).

TRIFASE minimizes a loss function that quantifies the discrepancy between the input experiment matrix and the product of three matrices containing the row and column clustering labels and the centroids, while also accounting for spatial covariance across observations through a fourth matrix. This loss function can be optimized with an iterative algorithm that reduces the main minimization problem into a series of convex subproblems. Although each algorithm step is not computationally demanding, the cost of the entire procedure can significantly grow with the data size. For this reason, in addition to the main algorithm, we propose a heuristic solution that significantly reduces the computational burden. Our approximated step revealed to be efficient and sufficiently precise in several simulation experiments. We also propose an alternative, stochastic step that helps the algorithm avoid suboptimal solutions.

The rest of the manuscript is structured as follows. In Section 2, we first review the main NMTF methods. Then, we focus on the spatial NMTF problem that integrates the data spatial structure and we present the TRIFASE procedure for minimizing the objective function and discuss its limitations. We then highlight the relation between the presented trifactorization problem and probabilistic clustering; we leverage this relation to develop a rule to update some of the parameters used in our model to handle the spatial correlation. Last, we propose two alternative algorithms that help solve the limitations of the main algorithm. In Section 3, we compare the proposed algorithms with a series of simulation experiments, providing a comprehensive view of the strengths and weaknesses of each approach. We further show that the classical NMTF problems lead to a substantial loss of information when applied to spatially correlated data. In Section 4, we apply TRIFASE on MALDI‐MSI data, discussing the results from a biological perspective. We conclude the article with Section 5, offering some final considerations and prospects for future works.

## 2 Methods

We denote as $T\subseteq R^{2}$ the spatial surface representing a tissue sample processed with a mass spectrometry imaging technology, where the abundance of n molecules is measured at p spatial locations, called *pixels*. A pixel j is identified through its spatial coordinates $s_{j}\in T$. Let X be the n×p experiment matrix whose entries are denoted with $X_{i,j}\in R_{+}$, the abundance of molecule i measured at pixel j, with i=1,⋯,n and j=1,⋯,p. The scope of our analysis is clustering the molecules according to their abundance in different tissue areas. In addition, we want to simultaneously partition the tissue into areas of similar molecules' abundance.

### 2.1 NMTF for Coclustering

NMTF aims at finding three nonnegative matrices, which we generally call $F\in R_{+}^{n\times K}$, $\mu \in R_{+}^{K\times R}$, and $G\in R_{+}^{p\times R}$, that well approximate a positive matrix X in terms of the Frobenius norm $\left|\right|\cdot {\left|\right|}_{F}$. In other words, we search for the solution of

(1) $$\min_{F\in R_{+}^{n\times K},\mu \in R_{+}^{K\times R},G\in R_{+}^{p\times R}}||X-F\mu G^{T}{\left|\right|}_{F}^{2}.$$

The goal of (1) is projecting through the matrix F the rows of X into a K‐dimensional latent space featured by the rows of μ, and the columns of X into an R‐dimensional latent space featured by the columns of μ through G. Ding et al. (2006) made one of the first attempts in the literature to cluster the rows and the columns of X using NMTF. Their proposal consists of setting F and G to be nonnegative and orthogonal matrices. Thus, under this construction, the normalized quantity $F_{i,k}/\sum_{k^{\prime }=1}^{K}F_{i,k^{\prime }}$ might be interpreted as the probability of row i belonging to cluster k, and $G_{j,r}/\sum_{r^{\prime }=1}^{R}G_{j,r^{\prime }}$ as the probability of column j belonging to cluster r. The problem of minimizing (1) under orthogonal constraints of F and G has been widely treated over the last two decades, with numerous algorithms that are compared in terms of clustering accuracy and computational cost (see, e.g., Pompili et al. 2014; Wang and Huang 2017; C̆opar, Zupan, and Zitnik 2019).

In alternative to the orthogonal constraints, Wang et al. (2011) and more recently, Song et al. (2021) assume F and G to be two cluster indicator matrices. They refer to this algorithm as *fast nonnegative matrix trifactorization* (FNMTF). In detail, they set $F_{i,k}=1$ when the ith row of X belongs to cluster k∈{1,⋯,K}, and $F_{i,k^{\prime }}=0$ for all $k^{\prime }\neq k$. In the same way, $G_{j,r}=1$ when the jth column of X belongs to cluster r∈{1,⋯,R}, and $G_{j,r^{\prime }}=0$ for all $r^{\prime }\neq r$. This formulation has some computational advantages with respect to the ones that consider F and G to be orthogonal, as the clustering operation can be performed for the rows and the columns in parallel, and it does not involve any matrix product. In addition, this formulation directly conveys the partition, as F and G already state the clusters to which the rows and columns of X are assigned without relying on any postprocessing step.

However, the FNMTF has some drawbacks. In fact, although the proposed algorithms are very computationally attractive, the assumption of independence of the rows and the columns of X strongly limits the range of applications, making the method inadequate for those scenarios that present some sources of dependence. This aspect is particularly evident in the field of spatial proteomics, where the molecule measurements exhibit clear spatial patterns over the tissue. A first attempt to extend the matrix trifactorization in this direction was proposed by Wang et al. (2011) through their algorithm LP‐FNMTF. They account for data dependency through two undirected graphs, one for the rows and one for the columns, and the loss function is penalized by two terms that include the normalized Laplacian matrices of the graphs. This idea was previously explored by Gu and Zhou (2009) but assumed F and G only to be nonnegative. This formulation should lead the algorithm to form the clusters based not just on how data points are similar (in terms of Euclidean norm, denoted as ||·|| in the next sections), but also considering the connections given by the graphs. The LP‐FNMTF algorithm remains computationally attractive, yet it lacks properties as it does not guarantee the decrease of the objective function. We elaborate on this aspect in Supporting Information Section 1.

### 2.2 Spatial NMTF

In this section, we propose an extension of the FNMTF problem for coclustering that accounts for the spatial structure of the data. We define an isotropic kernel matrix $\Sigma ={\left\{\Sigma_{j,j^{\prime }}\right\}}_{j,j^{\prime }=1,\cdots ,p}$, where each $\Sigma_{j,j^{\prime }}$ is a function of the spatial Euclidean distance between spatial points j and $j^{\prime }$. To this end, we set 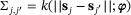, where k(·;φ) is a parametric stationary kernel parameterized by a d‐dimensional vector of positive parameters, $\varphi \in R_{+}^{d}$. The dimension of φ depends on the type of kernel employed: see Rasmussen and Williams (2008) for a complete review of the most used parametric kernels. In this work, we consider the exponential kernel parameterized by φ={τ,ϕ}, where τ represents the marginal variance and ϕ is the kernel scale. Thus, Σ=Σ(φ)=τK(S;ϕ), where $K\left(S;\phi \right)=\{\exp(-\left|\right|s_{j}-s_{j^{\prime }}\left|\right|/{\phi )\}}_{j,j^{\prime }=1\cdots ,p}$. In addition, let $\Sigma =LL^{T}$ be the Cholesky factorization of Σ, where L=L(φ) is a lower triangular matrix.

Being Ψ the space of all cluster indicator matrices, we recall the terms previously defined in Section 2.1 that appear in the minimization problem of Wang et al. (2011): $F\in \Psi^{n\times K}$ and $G\in \Psi^{p\times R}$ are the matrices containing the row and column clustering labels, and $\mu \in R_{+}^{K\times R}$ is the matrix of cocluster centroids. The *spatial NMTF* problem is

(2) $$\min_{F\in \Psi^{n\times K},\mu \in R_{+}^{K\times R},G\in \Psi^{p\times R},\varphi \in R_{+}^{d}}\left|\right|\left(X-F\mu G^{T}\right){\left(L^{-1}\right)}^{T}{\left|\right|}_{F}^{2}.$$

As mentioned in the previous section, if the positivity assumption of X is relaxed, $X\in R^{n\times p}$, then the minimization is still valid, and it is just necessary to consider $\mu \in R^{K\times R}$. Further explanation of the rationale for including L as it appears in Formula (2) will be provided in Section 2.3. The presented loss function is not convex, yet it can be broken into a series of simpler and convex problems by fixing all but one matrix at a time. We present now the TRIFASE procedure that iteratively solves (2). TRIFASE articulates in the following sequential updating steps:

**Step 1**: Fixing F, G, and φ, the updating rule of μ is
$$\mu \leftarrow {\left(F^{T}F\right)}^{-1}F^{T}X\Sigma^{-1}G{\left(G^{T}\Sigma^{-1}G\right)}^{-1}.$$
  Details about the derivation of the updating rule are given in Supporting Information Section 2.1. This problem is convex; therefore, given F, G, and Σ, it is guaranteed that an update of μ implies a decrease of the loss function (2).
**Step 2**: Fixing μ, G, and φ, and considering $\tilde{X}=X{\left(L^{-1}\right)}^{T}$ and $\tilde{G}=L^{-1}G$, update each row vector of F independently setting
$$F_{i,l}\leftarrow \left\{\begin{array}{cc} 1 & \;\text{if}\;l=\arg\min_{k=1,\cdots ,K}\left|\right|{\tilde{X}}_{i,.}-\mu_{k,.}{\tilde{G}}^{T}{\left|\right|}_{F}^{2},\\ 0 & \text{otherwise}, \end{array}\right.$$
  where ${\tilde{X}}_{i,.}$ and $\mu_{k,.}$ are, respectively, the ith row vector of $\tilde{X}$ and the kth row vector of μ. Details about the derivation of the updating rule are given in Supporting Information Section 2.2. Since every $F_{i,.}$ is updated by taking the clustering configuration that minimizes (2) among the K possible choices, we conclude that, given μ, G, and Σ, Step 2 guarantees the value of (2) to be nonincreasing. From our derivation in Supporting Information Section 2.2, one can see that the minimization problem of F can be broken into n subproblems, which consist of minimizing the loss function (2) with respect to each row $F_{i,.}$ separately, as also pointed out by Song et al. (2021).
**Step 3**: Fixing μ, F, and φ, the clustering labels in G cannot be updated independently as was done with F because of the presence of Σ. To keep a closed‐form updating rule, we can further break the minimization of G into p sequential problems, which update one row of G at a time. In formula,
(3) $$G_{j,l}\leftarrow \left\{\begin{array}{cc} 1 & \;\text{if}\;l=\arg\min_{r=1,\cdots ,R}\left|\right|\tilde{X}-F\mu {G^{\left(j,r\right)}}^{T}{\left(L^{-1}\right)}^{T}{\left|\right|}_{F}^{2},\\ 0 & \text{otherwise}, \end{array}\right.$$
  where $G^{\left(j,r\right)}$ equals the current value of G, but setting $G_{j,r}=1$ and $G_{j,r^{\prime }}=0,\text{for}r^{\prime }\neq r$. For every j, $G_{j,.}$ is updated by taking the clustering configuration that minimizes (2) among the R possible choices. Therefore, given μ, F, and Σ, Step 3 guarantees the value of (2) to be nonincreasing.
**Step 4**: The update of φ cannot be performed by considering only (2) because, fixing μ, F, and G, the loss function is monotonically decreasing in both elements of φ. To produce an estimate, we propose penalizing (2) by a quantity that is a function of the covariance matrix Σ. We will discuss this penalization approach more in detail in Section 2.3, based on some analogies of our method with the probabilistic clustering framework. Our approach guarantees the existence of a unique minimum of the loss function with respect to the gradient for τ, considering μ, F, G, and ϕ fixed while it is not guaranteed the existence of a unique stationary point with respect to ϕ.

Steps 1–4 must be iterated until a convergence criterion is reached. For example, one can stop the algorithm if the relative decrement of the loss function in (2) is smaller than a predefined threshold or, alternatively, if the clustering configuration does not change for a given number of iterations in a row.

By keeping ϕ fixed, the procedure described above guarantees the loss function (2) to be not increased. Each iteration involves minimizing with respect to the continuous variables μ and τ, which converge to a local minimum for each fixed F and G. In addition, F and G changes at each iteration are guaranteed not to increase the loss function. Therefore, the overall algorithm will converge to a point where μ and τ are at a local minimum for the current F and G, and F and G are at a value where the function is not increased by changing their values. Notice that there is no guarantee that the algorithm converges to the global minimum because (2) is not globally convex. The kernel scale ϕ is the only parameter that raises an exception because the gradient of (2) with respect to ϕ can present multiple stationary points. To overcome this issue, we will discuss in the next section a workaround that guarantees our algorithm runs only steps that involve a unique minimum conditioning on the other values.

The presented algorithm shows, however, some notable limitations in updating G because it requires p conditional updates, as described in Step 3. This request increases the chances of converging to a solution in correspondence of a point that deviates significantly from the global optimum. In addition, the computational cost of Step 3 becomes unsustainable as the number of pixels p grows, making it necessary to develop alternatives to explore the space of the partitions.

### 2.3 Analogies With the Probabilistic Clustering Framework

In the same spirit of Ding, He, and Simon (2005) and Ding, Li, and Jordan (2010), which highlighted the analogies between k‐means clustering and nonnegative matrix factorization, we notice that our spatial NMTF problem is linked to the probabilistic clustering framework. In fact, from the equivalence

$$\left|\right|\left(X-F\mu G^{T}\right){\left(L^{-1}\right)}^{T}{\left|\right|}_{F}^{2}=\mathrm{tr}\left[\left(X-F\mu G^{T}\right)\Sigma^{-1}{\left(X-F\mu G^{T}\right)}^{T}\right],$$

we recognize the elements of the kernel of a matrix variate Gaussian density function (Gupta and Nagar 1999). Recalling the notation $X∼N_{n,p}\left(A,B,C\right)$, where A is the n×p mean matrix and B and C are, respectively, the n×n covariance matrix of the rows and the p×p covariance matrix of the columns, we conclude that solving our spatial nonnegative trifactorization problem in (2) is equivalent to performing the maximum likelihood estimation of μ, F, G, and φ, assuming $X∼N_{n,p}\left(F\mu G^{T},I_{n},\Sigma \right)$. Rephrasing the TRIFASE loss function as the logarithm of the kernel of a matrix variate Gaussian model gives us a deeper comprehension of the role of L, and how our model extends the trifactorization algorithm of Wang et al. (2011). Specifically, it clarifies that $\Sigma =LL^{T}$ is the covariance matrix of the columns, capturing the dependence across spatial pixels based on their distances. Consequently, the trifactorization model in (1) corresponds to a matrix variate Gaussian model that treats both the rows and the columns as independent. In addition, performing Steps 1–3 iteratively is equivalent to maximizing the likelihood function with respect to the parameters μ, F, and G, until the convergence is reached.

Given these considerations, we propose to leverage the analogies of our method with the likelihood‐based clustering framework to derive, when possible, a closed‐form updating rule of φ. As an example, we consider the exponential kernel discussed in Section 2.2: a closed‐form updating solution of τ is possible after penalizing (2) by a term of nlog|Σ|. This quantity corresponds to the piece of the log‐transformed normalizing constant of a matrix variate Gaussian model that includes Σ. The resulting updating rule for τ is thus equivalent to the maximum likelihood estimator of τ under the Gaussian model

$$\tau \leftarrow \frac{\mathrm{tr}[\left(X-F\mu G^{T}\right)K{\left(S;\phi \right)}^{-1}{\left(X-F\mu G^{T}\right)}^{T}]}{np}.$$

We show in Supporting Information Section 2.4 that this solution corresponds to the unique stationary point of (2) with respect to τ, and it is a minimum conditional on the other variables. Regarding the scale parameter ϕ, it cannot be updated in closed form and requires to be updated numerically, or possibly, it can be fixed a priori to save computational cost and to guarantee that the minimization with respect to the gradients of μ, F, G, and τ gives a unique coordinate‐wise minimum.

### 2.4 An Approximate Version of Step 3

Among the algorithms reviewed by C̆opar, Zupan, and Zitnik (2019), the alternating least squares is a fast alternative for updating μ, F, and G. As both F and G have to be nonnegative, the algorithm treats them first as dense and real matrices, and then it forces nonnegativity using a heuristic. When applied to G, this rule derives from the following minimization problem:

$$\min_{G\in R^{p\times R}}\left|\right|\left(X-F\mu G^{T}\right){\left(L^{-1}\right)}^{T}{\left|\right|}_{F}^{2}.$$

The least squares solution that is forced to be nonnegative is

(4) $$G\leftarrow {\left[X^{T}F\mu {\left(\mu^{T}F^{T}F\mu \right)}^{-1}\right]}_{+},$$

where the operator ${\left[A\right]}_{+}$ sets each negative entry of the matrix A equal to zero. The solution in Formula (4) is independent of Σ.

Transferring this consideration into our clustering framework, we propose to update G treating the columns of X as independent. By removing Σ from Step 3 of Section 2.2, the update of G is performed as follows:

**Step 3A**: Keeping fixed μ and F, update the elements of G using
$$G_{j,l}\leftarrow \left\{\begin{array}{cc} 1 & \text{if}\;l=\arg\min_{r=1,\text{…},R}\left|\right|X_{.,j}-F\mu_{.,r}|{|}_{F}^{2},\\ 0 & \text{otherwise}. \end{array}\right.$$

Notice that this new step allows the pixel clustering to be performed in parallel, substantially reducing the computational cost. Unfortunately, Step 3A provides an approximate solution to the problem in (2); therefore, it does not ensure a monotonic decrement in the loss function.

### 2.5 A Stochastic Version of Step 3

In addition to the approximate solution proposed in Step 3A, we also suggest an alternative for Step 3 wherein the closed‐form minimization step is replaced by the stochastic generation of a new clustering partition of the columns. In Section 2.3, we have already outlined the analogies between the loss function of TRIFASE and probabilistic clustering. If the unknown G is treated as a random variable, the maximization step can be substituted with a simulation from the conditional distribution G|X,F,μ,Σ. This approach, commonly known in the statistical literature as Stochastic EM (Celeux and Govaert 1992), is widely recognized as an alternative to maximization when closed‐form solutions are not feasible (Bouveyron et al. 2018; Casa et al. 2021; Sottosanti and Risso 2023). Within our framework, the solution returned by our estimation algorithm strongly depends on the initialization due to the presence of multiple local minima in the objective function (2). Estimation algorithms based on stochastic moves, such as the Stochastic EM and the *simulated annealing* (van Laarhoven and Aarts 1987; Section 4.2 of Celeux and Govaert 1992), were introduced to aid the estimation algorithm avoid local minima that are substantially far from the global minimum. The new step works as follows:

**Step 3S**: Keeping fixed F, μ, and φ, for j=1,⋯,p define
$$\omega_{j,r}=\exp\{-||\tilde{X}-F\mu {G^{\left(j,r\right)}}^{T}{\left(L^{-1}\right)}^{T}{\left|\right|}_{F}^{2}\}$$
  and $\pi_{j,r}=\omega_{j,r}/\sum_{r'=1}^{R}\omega_{j,r'}$. Let ${\tilde{g}}_{j}$ be a draw from a multinomial distribution $M\{1,\left(\pi_{j,1},\cdots ,\pi_{j,R}\right)\}$. Set $G_{j,.}\leftarrow {\tilde{g}}_{j}$.

The final estimate of G corresponds to the clustering partition that guaranteed the lowest value of (2). Note that one can also propose the same strategy to update the row cluster assignments, which we will refer to as Step 2S.

Similarly to Step 3A, Step 3S does not assure continuous minimization of the loss function, but it considers the correlation of the columns of X when performing the clustering. While the convergence rule outlined in Section 2.2 remains applicable, we recommend checking the algorithm's effective convergence, displaying the loss function values across the iterations.

## 3 Simulation Studies

We propose an extensive simulation study to validate the performance and robustness of our coclustering algorithm. We deployed an ample range of parameter simulation settings to ensure a wide variety of scenarios. The scope is to benchmark the different updating strategies of TRIFASE outlined in Section 2 in terms of goodness of kernel parameter estimation, clustering accuracy, and computational cost.

Recall that the row clustering step of TRIFASE can be performed either with the so‐called classification rule (C, Step 2) or with the stochastic move (S, Step 2S) and that the column clustering can be exact (C, Step 3), stochastic (S, Step 3S), or approximate (A, Step 3A). We compare the results obtained with our method under all six resulting combinations. Moreover, to compare TRIFASE with other clustering techniques, we consider the k‐means algorithm applied on the matrix rows and columns independently (Km) and the FNMTF algorithm proposed by Wang et al. (2011) (W), for a total of eight different clustering methods.

We now detail the data‐generating process we adopted to simulate the synthetic data sets for our tests. In what follows, we first set the true number of row and column clusters to $K^{\mathrm{true}}=3$ and $R^{\mathrm{true}}=4$, respectively. We fixed the total number of rows n equal to 90 while considering two possible settings for the number of columns: a low‐dimensional scenario with p=100 columns and a high‐dimensional scenario with p=1000 columns. This latter case provides a first intuition of how the different methods work when tackling complex, large, real biological data sets. Moreover, we considered unbalanced block structure scenarios in which the clusters' dimensions are randomly generated. In other words, we partitioned the 90 rows into K=3 clusters with random cardinality, ranging between 5 and 53 (with an average of 30). Similarly, we partitioned the 100 (1000 columns) into R=4 clusters. The random sizes we obtained range between 5 and 61 (56 and 582) for columns, with an average of 25 (250). We considered two types of kernel matrices: the first considers $\Sigma^{\mathrm{true}}=\tau^{\mathrm{true}}K\left(S;\phi^{\mathrm{true}}\right)$, that is, an exponential kernel as defined in Step 4 of TRIFASE, with marginal variance $\tau^{\mathrm{true}}=3$ and the scale parameters $\phi^{\mathrm{true}}=10$, and the second considers $\Sigma^{\mathrm{true}}=I_{p}$, representing a scenario without spatial correlation.

The data‐generating scheme is the following. First,  blocks of spatial pixels are assembled in a rectangle. Their position is devised to induce higher spatial correlation to columns in the same cluster. Then, the cluster indicator matrices $F^{\mathrm{true}}$ and $G^{\mathrm{true}}$ are generated as described above, and the centroid matrix $\mu^{\mathrm{true}}={\left\{\mu_{k,r}^{\mathrm{true}}∼N\left(m_{k,r},1\right)\right\}}_{k=1,\cdots ,K;r=1,\cdots ,R}$, where $m_{k,r}$ is set a priori (see Figure S1). Last, the experiment matrix is drawn from

$$X|\mu^{\mathrm{true}},F^{\mathrm{true}},G^{\mathrm{true}}∼N_{n,p}\left\{F^{\mathrm{true}}\mu^{\mathrm{true}}{\left(G^{\mathrm{true}}\right)}^{T},\;I_{n},\;\Sigma^{\mathrm{true}}\right\}.$$

The fact that X also contains negative values is not a limitation as TRIFASE can be applied to this case by simply relaxing the boundaries on the estimation of μ, as mentioned in Section 2.2.

We generated 30 data sets using the exponential kernel and another 30 assuming independence across the columns, both in the cases with p=100 and p=1000. For every data set, to test the impact of different model setups and starting points on our estimation strategy, we ran TRIFASE under all possible combinations of the following model parameters: $K\in \{K^{\mathrm{true}},2K^{\mathrm{true}},3K^{\mathrm{true}}\}$ and $R\in \{R^{\mathrm{true}},2R^{\mathrm{true}},3R^{\mathrm{true}}\}$. Thus, the first two options of K and R represent the case where the model parameters are fixed identically to the ground truth. In comparison, the other options represent cases where the number of row or column clusters is misspecified, as we expect to be the case in real scenarios. We do not consider the cases of $K<K^{\mathrm{true}}$ and $R<R^{\mathrm{true}}$ as, in practice, it is preferable to initiate the algorithm by overestimating the number of clusters and allowing the model to retain only the ones that are needed to fit the data, rather than underestimating the number of clusters in advance. Indeed, when not guided by external, problem‐specific information, one may want to let the algorithm empty some clusters, distinguishing between fitted and estimated ones (i.e., the ones that contain at least an observation). To grant computational efficiency, we decided not to estimate ϕ, but to evaluate our model on a grid of multiple values set a priori. When the spatial dependence is assumed, we considered the grid of values {0.1,10,20}, while for the scenario without spatial correlation, we considered the values {0.001,0.1,10}. To summarize, we investigated six versions of TRIFASE with 27 different parameter combinations on four sets of data sets: two sets of 30 data sets with spatial dependence (for p=100 and p=1000 pixels) and two sets of 30 data sets with independent columns (also for p=100 and p=1000 pixels). Table S1 summarizes the 162 possible configurations of TRIFASE we have performed.

Under each parameter setting, we ran the clustering algorithms 50 times considering different random starting points of F, μ, and G, and we retained the best results in terms of the loss function. The algorithms stop if a step does not lead to a relative variation in the loss function larger than 0.1%. For the approximated algorithm (A), as the loss function is not bound to be monotonically decreasing, we retain the clustering solution that led to the minimum value of the loss in the entire run. All our experiments are performed on a server running an Intel Xeon Gold 6230R CPU with 92 GB of memory. The script developed for the simulation experiments and the real data analysis is available at the GitHub repository andreasottosanti/TRIFASE.

### 3.1 Simulation Results

We begin our discussion of the results presenting the outcomes obtained on the data sets generated with spatial dependence, thus assuming $\Sigma^{\mathrm{true}}=\tau^{\mathrm{true}}K\left(S;\phi^{\mathrm{true}}\right)$. We measured the clustering accuracy with the *classification error rate* (CER). This index quantifies the disagreement between a benchmark and an estimated clustering configuration: the closer CER is to 0, the larger the agreement between the true and the estimated clusters (Witten and Tibshirani 2010). To ease the intuition, we used an index measuring the concordance of clustering to present our results, obtained as 1‐CER. In addition, we also compared the methods in terms of computational time (expressed in seconds), estimated number of nonempty clusters, and estimated τ.

**FIGURE 2:**
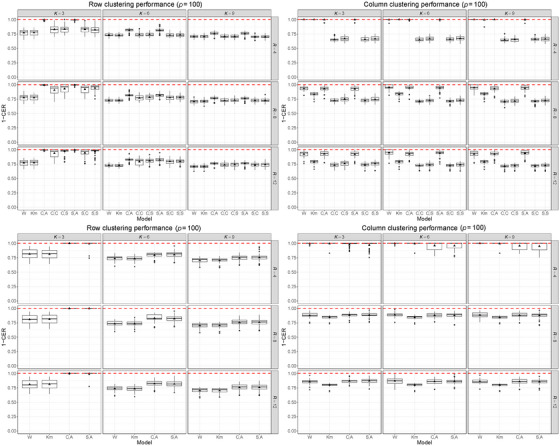
Results obtained on 30 simulated data sets of dimension 90×100 (top row) and on 30 simulated data sets of dimension 90×1000 (bottom row). All the data sets were generated assuming $K^{\mathrm{true}}=3$ and $R^{\mathrm{true}}=4$ and with spatial correlation among columns ($\tau^{\mathrm{true}}=3$, $\phi^{\mathrm{true}}=10$). We employed six different estimation strategies of TRIFASE (C,A; C,C; C,S; S,A; S,C; S,S), k‐means (Km), and FNMTF (W). The graphs display the concordance (in terms of 1‐CER) of the estimated row clustering (left column) and column clustering (right column) with the reference labels, assuming different values of K and R. The red lines denote the perfect matching between true and estimated clusters. For the analysis of the 30 data sets of dimension 90×1000, we restricted our attention only to the versions of TRIFASE that make use of Step 3A (C,A; S,A) to reduce the computation burden. Every version of TRIFASE was estimated setting ϕ=10.

We begin illustrating in Figure 2 the clustering accuracy for the 30 data sets with p=100 (top row) and p=1000 (bottom row). Comparing the results obtained at different values of p allows us to show how the increment of dimensionality impacts the clustering accuracy. The left column of the plot displays the clustering accuracy obtained on the rows, and the right column the clustering accuracy on the columns, obtained with the eight models considered. We used the ground truth value $\phi =\phi^{\mathrm{true}}=10$. Results considering ϕ=0.1 and ϕ=20 are reported in Supporting Information Section 3.1. Due to the large sequential operations required to perform the column clustering, the algorithms that implement the column allocation using the stochastic (S) and classification (C) steps are the slowest. For this reason, we did not consider these implementations in the analysis of the simulated data sets with p=1000, restricting our attention only to (C,A) and (S,A).

Results show that the versions of TRIFASE that implement Step 3A (C,A; S,A) are the ones that achieve the best results in terms of clustering accuracy both on the rows and on the columns, even when the number of clusters is misspecified. We notice that FNMTF and k‐means achieve good classification results when clustering the columns but fail to recover the groups on the rows. Therefore, the classification of the columns appears not to be affected by the spatial correlation, which explains why our Step 3A works well. In other words, results in Figure 2 provide two key messages. First, in the process of row clustering (i.e., clustering the lipids), it is essential to properly account for the spatial correlation across the columns (i.e., the pixels). Neglecting this aspect results in poor clustering performance (see right column of Figure 2). On the other hand, when performing column clustering (i.e., clustering the pixels), not considering the correlation does not lead to a substantial loss in the clustering performance. This is evident from the good clustering performance achieved through the versions of TRIFASE that implement Step 3A, as well as the competing methods FNMTF and k‐means (see left column of Figure 2). The versions of TRIFASE that implement Steps 3 and 3S (C,C; C,S; S,C; S,S) tend to achieve better clustering results on the rows when R is taken larger than the true value $R^{\mathrm{true}}$. In addition, they fail to recover the correct partition of the columns.

**FIGURE 3:**
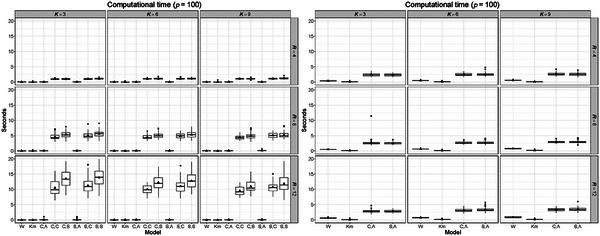
Analysis of the computational cost (in seconds) on 30 simulated data sets of dimension 90×100 (left panel) and on 30 simulated data sets of dimension 90×1000 (right panel), varying the number of row and column clusters. Every version of TRIFASE was estimated setting ϕ=10.

We also evaluated the methods based on their computational cost. Understanding the computational efficiency of each method is crucial, especially when we need to tune some parameters of TRIFASE, like K, R, and ϕ. The two panels of Figure 3 show the distributions of the computation speeds of the compared coclustering methods obtained on the data sets with p=100 pixels and p=1000 pixels. These results are based on different combinations of K and R values, with ϕ set to $\phi^{\mathrm{true}}=10$. Additional results obtained with the models with ϕ=0.1 and ϕ=20 are displayed in Supporting Information Section 2.

The versions (C,A) and (S,A) of TRIFASE are practically equivalent in terms of estimation time and are the most efficient among the multiple implementations proposed in this article. Their estimation time is also comparable to FNMTF and k‐means. For this reason, we further investigated the scalability of the version (C,A) under different experimental conditions, varying the number of rows and columns of the data matrix. Therefore, we generated 30 data sets considering n=150,750,1500 and p=200,1000,2000, while $K^{\mathrm{true}}=3$ and $R^{\mathrm{true}}=4$. Figure S5 displays the estimation time in seconds under the different experimental conditions considered, using $K\in \{K^{\mathrm{true}},3K^{\mathrm{true}}\}$ and $R\in \{R^{\mathrm{true}},3R^{\mathrm{true}}\}$. These results provide an initial idea of the computational cost needed when dealing with real biological data sets (see also Table 1).

**FIGURE 4:**
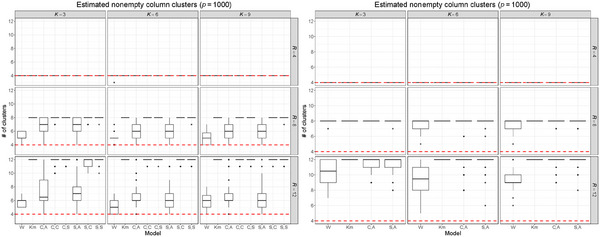
Distribution of the number of nonempty column clusters obtained on 30 simulated data sets of dimension 90×100 (left column) and on 30 simulated data sets of dimension 90×1000 (right column), varying the number of row clusters K and column clusters R used by the models. Red lines denote $R^{\mathrm{true}}$. Every version of TRIFASE was estimated setting ϕ=10.

Another important aspect that we investigated is the ability of the model to eliminate unnecessary clusters and accurately estimate the true number of row and column clusters. This is crucial in practical applications, where the real number of clusters is often unknown. Ideally, an approach that initially overestimates the number of clusters and then selects only the relevant ones would be beneficial, as it avoids the need to estimate the model multiple times with different parameter configurations. Figure 4 shows the distribution of the number of nonempty column clusters obtained under different combinations of K and R from the eight models considered. We conclude that the versions of TRIFASE that employ Step 3A (C,A; S,A) are the most effective at selecting the true number of clusters when p is moderate. FNMTF often appears to select the true number of column clusters. However, this property is not sufficient to correctly recover the row clusters, as we have already shown in the left column of Figure 2. All the algorithms appear to have more difficulties in recovering the truth when p is large. Additional results about the distribution of nonempty row clusters, obtained also with the models with ϕ=0.1 and ϕ=20, are displayed in Supporting Information Section 2.

Furthermore, Figure S9 displays the distribution of the estimated kernel parameter values τ, obtained for every combination of K and R, both in the scenarios with p=100 and p=1000. The versions (C,A) and (S,A) of TRIFASE recover the right value of τ when $\phi =\phi^{\mathrm{true}}$ and return a less biased estimate than the stochastic and classification versions when ϕ is set larger than $\phi^{\mathrm{true}}$.

Last, we investigated two additional aspects of TRIFASE. The first is its performance in a scenario where the columns are not spatially correlated, thus taking $\Sigma^{\mathrm{true}}=I_{p}$. As we expected, when ϕ is set sufficiently small, TRIFASE can retrieve the correct block structure of the data. A more detailed description of the results is reported in Supporting Information Section 3.2. The second aspect is related to the empirical evaluation of the convergence properties of the different versions of TRIFASE to the same local solution. We report in top row of Figure S13 the distribution of the final loss function values obtained by running each version of TRIFASE 50 times on the same data set. In the central and bottom rows of the same figure, we additionally display the distribution of (1‐CER) values comparing row and column partitions at the loss function minimum with those from the remaining 49 runs to provide more evidence about the stability of the estimation of the coclustering partition. The figures clearly show that most of the 50 runs of the versions (C,A) and (S,A) converge to the same local solution, with only a few runs reaching very distant convergence points. In contrast, the remaining four versions reach the minimum level in only a few of the 50 runs, with the majority converging to suboptimal points. We therefore conclude that, based on our simulation studies, not only did (C,A) and (S,A) return the best results in terms of clustering accuracy and computational time, but they also demonstrated a notable stability in terms of convergence to the same solution when the estimation algorithm is initialized from different starting points.

## 4 Application to MALDI‐MSI Data

**FIGURE 5:**
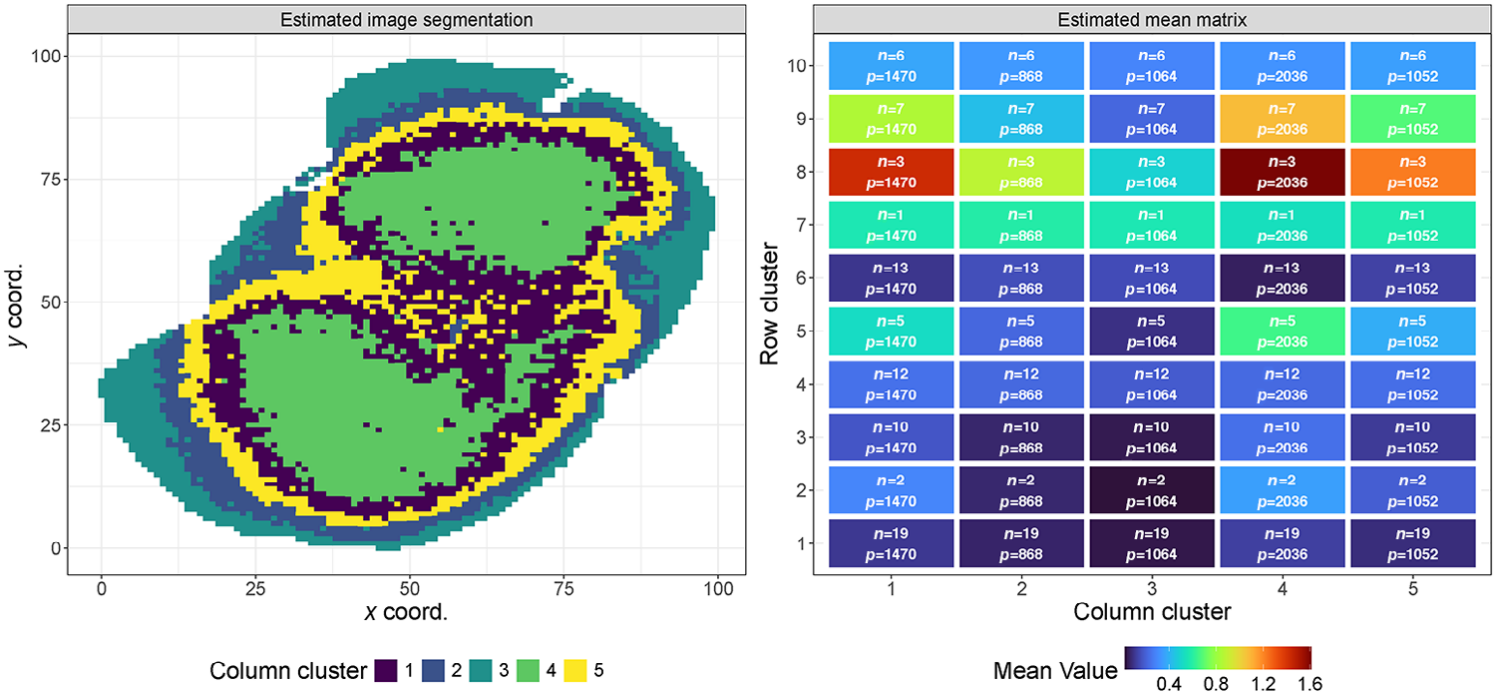
Left panel: estimated segmentation of the pixels that compose the image of the brain slice of interest. Right panel: heatmap of the estimated mean matrix μ, highlighting the detected coclustering patterns. In each block, we report the number of signals (n) and pixels (p) in the corresponding cocluster.

In this section, we present the results obtained fitting TRIFASE to the MALDI‐MSI data set. Recall that the data set is characterized by 78 rows representing the lipid signals observed over approximately 6500 columns representing the spatially related pixels. In particular, we fitted our model after transforming the original data Y as $X=\log(\sqrt{Y}+1)$, mapping the abundances of molecules on the real line R, while inducing symmetry of the overall distribution. As in the simulation study presented in the previous section, we ran the algorithm 50 times assuming K=10, R=5, and different random starting points, retaining the model estimate corresponding to the smallest value of the loss function. To assess the strength of the spatial correlation, we fitted 16 different TRIFASE models characterized by different fixed values of ϕ∈{0.5,0.7,0.9,1,3,5,10,20} and by the two fastest versions of TRIFASE (C,A) and (S,A). The results are displayed in Table 1. The minimum loss value is obtained when setting ϕ=0.9, suggesting the presence of mild spatial correlation. We now discuss the results from that particular run. In the left panel of Figure 5, we show the retrieved image segmentation. Our method captures the critical differences between the interbrain formations, such as hippocampus (cluster 5) and white matter regions (clusters 1 and 4), and the external ones, that is, the gray matter (clusters 2 and 3).

**TABLE 1:** Details of the best runs, out of 50, of the TRIFASE model obtained fixing different values of ϕ, estimated with the two fastest algorithm versions (C,A) and (S,A) on the real data set. Columns from left to right denote: the value of ϕ, the algorithm's version, the minimum value of the loss function reached, the computational time, the number of iterations, the estimate of τ, and the number of nonempty row and column clusters.

ϕ	Algorithm	Min loss	Time (sec.)	Max iter	Est. τ	#Row clusters	#Col. clusters
0.50	S,A	2215.63	363.78	14	0.01	10	5
0.50	C,A	2222.25	405.71	15	0.01	10	5
0.70	S,A	1942.81	238.82	14	0.01	10	5
0.70	C,A	1940.56	308.60	13	0.01	10	5
0.90	S,A	1949.23	258.24	10	0.01	10	5
0.90	C,A	1913.08	266.41	12	0.01	10	5
1.00	S,A	1972.60	261.69	10	0.01	10	5
1.00	C,A	2031.47	193.74	8	0.01	10	5
3.00	S,A	4068.19	260.03	12	0.02	10	5
3.00	C,A	3730.16	303.29	12	0.02	8	5
5.00	S,A	5620.04	251.20	10	0.03	10	5
5.00	C,A	5597.56	247.59	14	0.03	10	5
10.00	S,A	11,697.40	214.24	7	0.07	10	5
10.00	C,A	11,724.49	182.33	6	0.07	10	5
20.00	S,A	23,453.85	201.38	10	0.14	10	5
20.00	C,A	23,522.54	191.06	8	0.14	10	5

The right panel displays the estimated μ matrix, summarizing the main attributes of the coclustering solution. Column clusters 1, 4, and 5 exhibit similar activation patterns and have the highest activation abundance. In contrast, column clusters 2 and 3 contain low‐abundance signals. Examining the row clusters, groups 8 and 9 include m/z values that are the most active in the brain, particularly within the white matter. Another notable aspect is represented by row clusters 1, 4, and 10, which consist of lipid groups equally expressed across the five different column clusters. These clusters with homogeneous molecular expression throughout the tissue are particularly important, as they group nondiscriminatory signals, thus identifying the m/z values not associated with specific biological pathways characteristic of the different brain areas (e.g., hippocampus, white and gray matter regions, and the peripheral ones). In addition, row cluster 7 contains a single lipid signal that mostly characterizes the peripheral cortical regions of the mouse brain tissue sample (column clusters 2, 3, and 5).

**FIGURE 6:**
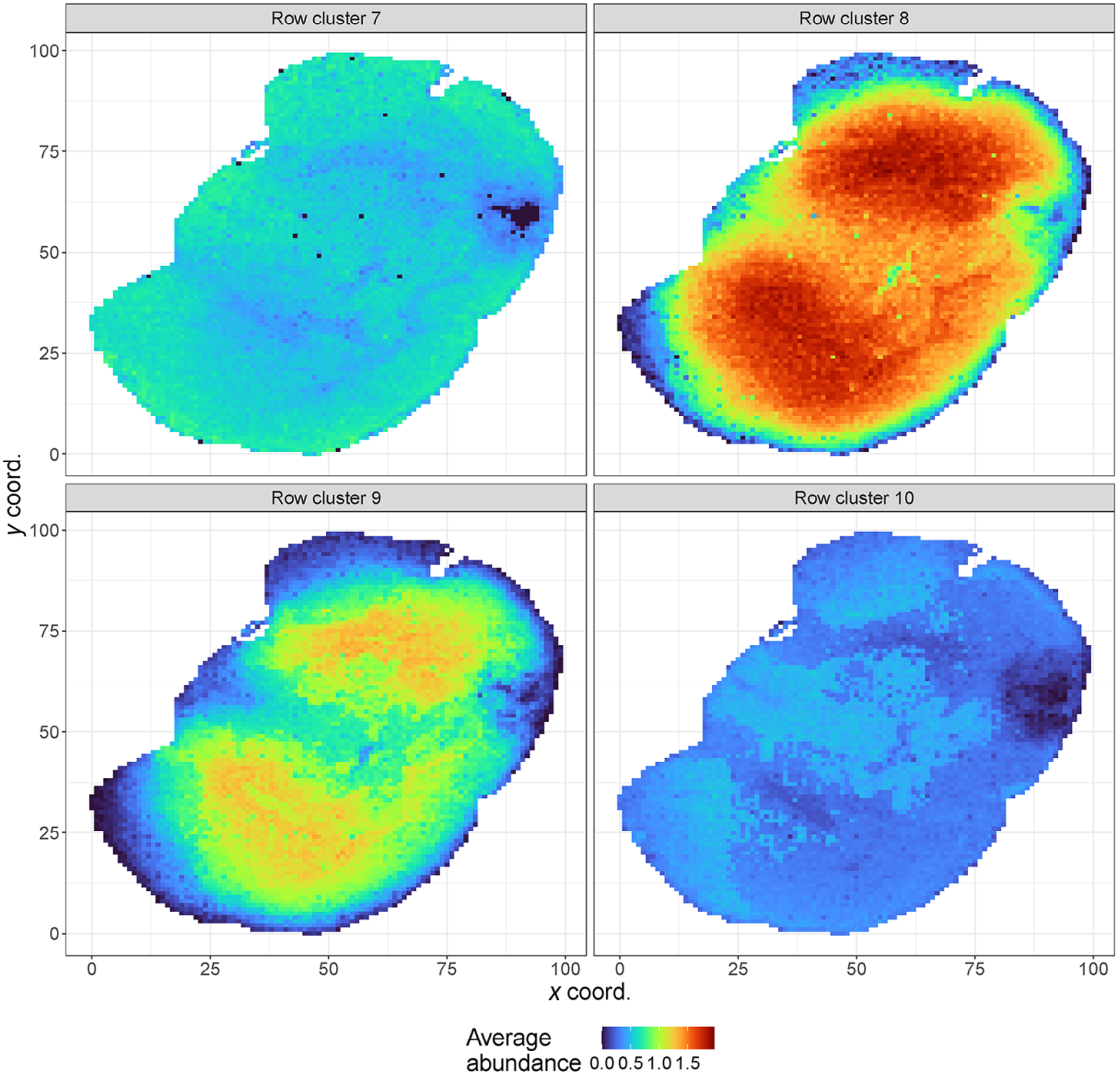
Molecular expressions of the row clusters ranging from 7 to 10 across the entire tissue sample. Pixels are colored according to the mean of the abundance of the m/z assigned to each row cluster.

Last, we inspect in detail the row clusters ranging from 7 to 10. Figure 6 shows that these four clusters are characterized by different patterns of molecular abundance across the analyzed tissue sample. The lipids in row clusters 8 and 9 are mainly associated with the central part of the brain section, while the only protein in the seventh cluster highlights the peripheral regions. Lipids in cluster 9 are less abundant in the central part than those in row cluster 8 but clearly show no activation in the outermost cortical area, consequently characterizing only the white matter regions. Conversely, the 7th and 10th row clusters give a deeper segmentation of the gray matter, which performs the function of selecting and initiating information that travels along the nervous system and also serves as the starting point of motor inputs. The single lipid in row cluster 7 increases its expression in the cortex regions (the outermost area of the encephalon), while row cluster 10 highlights the thalamus and hypothalamus part of the mouse brain. Finally, in Section 3.4 of the Supporting Information, we report additional figures displaying the coclustering, jointly considering the partition of rows and columns.

With this application, we emphasized the opportunity that the TRIFASE method gives us to investigate the biological sample under a different perspective. Unlike simple algorithms like the k‐means method, routinely used to process biological imaging data, TRIFASE allows a simultaneous grouping of rows and columns to detect interesting brain areas and lipid signal subsets. As shown in Figure 6, we can investigate the characterizing molecules of a specific clustering of pixels, thus identifying the signals most expressed in the given cluster. The goodness of the model we present is supported by the biological results already found in the literature. According to the results reported by Denti et al. (2022), we highlighted groups of lipids that showed up well in the brain's white matter, located in the brain slice's central part, and a group of signals characteristic of the cortex. These results confirm that our findings are consistent with the known biochemistry of brain tissues. While this study focuses on known histopathological regions as a proof of concept, it underlines the molecular behavior over the different clusters and lays the foundation for a more comprehensive characterization of the murine brain tissue. The proposed coclustering approach has the potential to be applied in clinical studies, where the molecular data can be integrated with spatial information to help identify  new subgroups of histological areas with different molecular features and possible biological behavior.

## 5 Discussion

We have introduced TRIFASE, a coclustering method based on NMTF, which is able to incorporate spatial information in the estimation process. While detailing our estimation algorithm, we presented three clustering strategies: exact, stochastic, and approximate. We compared the performance of our proposals with extensive simulations and applied our algorithm to a large biomedical imaging data matrix.

Although the primary focus of our study is mass spectrometry data, we underline that TRIFASE has broad applications in other biological contexts, such as spatial transcriptomic experiments (Williams et al. 2022). In these experiments, the expression levels of thousands of genes are measured at multiple spatial locations, effectively yielding an input matrix similar to the one discussed in Section 2. Moreover, as stated in Section 1, Denti et al. (2022) have recently demonstrated the feasibility of performing spatial multiomics analysis with MALDI‐MSI on a single tissue section, encompassing lipids, tryptic peptides, and N‐glycans molecules. Consequently, given the large amount of different omics data, the demand for comprehensive data integration in multiomics studies is expected to rise significantly in the coming years. To address this demand, we will extend our TRIFASE algorithm to jointly model different omics, uncovering hidden molecular patterns resulting from the relationship between these multiple molecular levels. Thus, enhancing our understanding of complex biological processes opens up new avenues for research in fields such as personalized medicine, systems biology, and biomarker discovery.

The optimization problem we explored poses significant challenges, given its nonconvex nature. Therefore, on the methodological side, potential future enhancements to the algorithm would stem from a rigorous formulation of convergence guarantees, which are suggested to be present by our extensive simulation results. Another direction may involve investigating alternative approaches for pixel clustering. This avenue could include deriving multiplicative updating rules for the spatial NMTF problem, similar to what was done by Gu and Zhou (2009) and C̆opar, Zupan, and Zitnik (2019) for the NMTF problem. Alternatively, one could explore other stochastic optimization algorithms in Step 3S, such as the simulated annealing (van Laarhoven and Aarts 1987). Moreover, to increase the efficiency of our proposal, we could resort to approximations of the spatial kernel matrix, borrowing ideas for efficient Gaussian process computation, such as the nearest‐neighbor Gaussian process (Datta et al. 2016). Lastly, the application of shrinkage techniques, model‐based clustering, hypothesis testing, and their combination (as proposed, e.g., in Denti et al. 2021, 2023) constitute potential enhancements to the proposed model that will be explored in the future.

## Conflicts of Interest

The authors declare no conflicts of interest.

### Open Research Badges

This article has earned an Open Data badge for making publicly available the digitally‐shareable data necessary to reproduce the reported results. The data is available in the Supporting Information section.

This article has earned an open data badge “**Reproducible Research**” for making publicly available the code necessary to reproduce the reported results. The results reported in this article were reproduced partially due to data confidentiality issues.

## Supporting information

Supporting Information

Supporting Information

## Data Availability

The data that support the findings of this study originated from Denti et al. (2022) and are available from the corresponding author upon reasonable request.
