# Supplementary material for: Spatially Informed Nonnegative Matrix Trifactorization for Coclustering Mass Spectrometry Data
Source: Biom J. 2025 Mar 19;67(2):e70031. doi: 10.1002/bimj.70031 (PMC11921289; doi:10.1002/bimj.70031)
Supplement: Supplementary file 1 — Supporting Information [file BIMJ-67-e70031-s001.zip › TRIFASE_Code/README.html]

Guide to the code folder


Code 

- Show All Code
- Hide All Code

# Guide to the code folder

#### Andrea Sottosanti, Francesco Denti, Stefania Galimberti, Davide Risso, Giulia Capitoli

- Introduction
- Section 1
  - Pakages required
  - Not on `CRAN`
    packages
- Section 2
  - Section 2.1 - Reproduce data
    generation and simulation studies
  - Section 2.2 - Simulation
    scripts
  - Section 2.3 - Simulation
    results
  - Section 2.4 - Master R
    script
- Section 3
  - Real data
    results
- Section 4

---

This folder contains the scripts to reproduce the results presented
in the main text and supplementary material of

- *Andrea Sottosanti, Francesco Denti, Stefania Galimberti, Davide
  Risso, Giulia Capitoli*;  
  Spatially informed non-negative matrix tri-factorization for
  co-clustering mass spectrometry data,  
  **Biometrical Journal**, 2024

---

# Introduction

- Packages used and version of R are detailed in Section
  1.
- Section 2 contains a guide to fully reproduce the
  results of the simulations, as well as all the graphs and the tables
  included in the article.
- Section 3 contains a guide on real data
  analysis.
- Section 4 contains the remaining files.

# Section 1

The required packages are listed below.

Most of them are available on `CRAN` and can be easily
installed using `install.packages("name-of-package")`. A full
guide to the installation of packages that are not on `CRAN`
can be found here.

### Pakages required

```
library(dplyr)
library(stringr)
library(ggplot2)
library(lattice)
library(Matrix)
library(TRIFASE)
library(Rfast)
library(reshape2)
library(latex2exp)
library(patchwork)
require(scales)
library(fossil)
library(xtable)
library(viridis)
library(kableExtra)
library(formattable)
library(knitr)
```

```
sessionInfo()
```

```
## R version 4.4.0 (2024-04-24)
## Platform: x86_64-apple-darwin20
## Running under: macOS 15.1
## 
## Matrix products: default
## BLAS:   /Library/Frameworks/R.framework/Versions/4.4-x86_64/Resources/lib/libRblas.0.dylib 
## LAPACK: /Library/Frameworks/R.framework/Versions/4.4-x86_64/Resources/lib/libRlapack.dylib;  LAPACK version 3.12.0
## 
## locale:
## [1] en_US.UTF-8/en_US.UTF-8/en_US.UTF-8/C/en_US.UTF-8/en_US.UTF-8
## 
## time zone: Europe/Rome
## tzcode source: internal
## 
## attached base packages:
## [1] stats     graphics  grDevices utils     datasets  methods   base     
## 
## other attached packages:
##  [1] knitr_1.49         formattable_0.2.1  kableExtra_1.4.0   viridis_0.6.5     
##  [5] viridisLite_0.4.2  xtable_1.8-4       fossil_0.4.0       shapefiles_0.7.2  
##  [9] foreign_0.8-87     maps_3.4.2         sp_2.1-4           scales_1.3.0      
## [13] patchwork_1.3.0    latex2exp_0.9.6    reshape2_1.4.4     Rfast_2.1.0       
## [17] RcppParallel_5.1.9 RcppZiggurat_0.1.6 Rcpp_1.0.13-1      TRIFASE_0.0.2     
## [21] Matrix_1.7-1       lattice_0.22-6     ggplot2_3.5.1      stringr_1.5.1     
## [25] dplyr_1.1.4       
## 
## loaded via a namespace (and not attached):
##  [1] sass_0.4.9        utf8_1.2.4        generics_0.1.3    xml2_1.3.6       
##  [5] stringi_1.8.4     digest_0.6.37     magrittr_2.0.3    evaluate_1.0.1   
##  [9] grid_4.4.0        fastmap_1.2.0     plyr_1.8.9        jsonlite_1.8.9   
## [13] gridExtra_2.3     fansi_1.0.6       jquerylib_0.1.4   cli_3.6.3        
## [17] rlang_1.1.4       munsell_0.5.1     withr_3.0.2       cachem_1.1.0     
## [21] yaml_2.3.10       tools_4.4.0       parallel_4.4.0    colorspace_2.1-1 
## [25] vctrs_0.6.5       R6_2.5.1          lifecycle_1.0.4   htmlwidgets_1.6.4
## [29] pkgconfig_2.0.3   pillar_1.9.0      bslib_0.8.0       gtable_0.3.6     
## [33] glue_1.8.0        systemfonts_1.1.0 xfun_0.49         tibble_3.2.1     
## [37] tidyselect_1.2.1  rstudioapi_0.17.1 farver_2.1.2      htmltools_0.5.8.1
## [41] svglite_2.1.3     rmarkdown_2.29    compiler_4.4.0
```

### Not on `CRAN` packages

One package is not available on `CRAN`:
`TRIFASE`. It can be installed from the GitHub repository
`https://github.com/andreasottosanti/TRIFASE` and thus
requires the R package `devtools.` Alternatively, it can be
installed using the R package `TRIFASE_0.0.2.tar.gz`.

```
library(TRIFASE)
install.packages("~/TRIFASE_0.0.2.tar.gz", repos = NULL, type = "source")
```

# Section 2

This section describes the steps to fully reproduce the simulation
results of the study.

The steps described in this section require the installation of
packages listed in Section 1. Computational time
depends on the cores available for parallel computation. To adjust the
number of cores, change the variable `n.cores`.

### Section 2.1 - Reproduce data generation and simulation studies

Set the parent directory `TRIFASE_Code` as the working
directory. All the functions needed for the simulation study can be
imported into the R Environment:

```
source("SIMULATION_STUDIES/AUX/sim_coordinates_matrix.R")
source("SIMULATION_STUDIES/AUX/a_Scenarios.R")
source("SIMULATION_STUDIES/AUX/b_GenerateData.R")
source("SIMULATION_STUDIES/AUX/c_Estimate.R")
source("SIMULATION_STUDIES/AUX/c_Estimate_Scalability.R")
source("SIMULATION_STUDIES/AUX/d_ExtractResults.R")
source("SIMULATION_STUDIES/AUX/e_setParameters.R")
source("SIMULATION_STUDIES/AUX/f_Performance_index.R")
```

After that, you can run the simulation scripts.

### Section 2.2 - Simulation scripts

The user can run one of the R files listed below:

- `run_functions_Simulation_study.R` (operating in
  `SIMULATION_STUDIES/SIMULATION_RESULTS/Simulation_Study`)
- `run_functions_Add_sim_study_1.R` (operating in
  `SIMULATION_STUDIES/SIMULATION_RESULTS/Add_sim_study_1`)
- `run_functions_Add_sim_study_2.R` (operating in
  `SIMULATION_STUDIES/SIMULATION_RESULTS/Add_sim_study_2`)
- `run_functions_Add_sim_study_3.R` (operating in
  `SIMULATION_STUDIES/SIMULATION_RESULTS/Add_sim_study_3`)
- `run_functions_Add_sim_study_4.R` (operating in
  `SIMULATION_STUDIES/SIMULATION_RESULTS/Add_sim_study_4`)

Note that the order in which the scripts are executed is not
relevant. These codes have a common structure, as they:

- 1. save the parameters used to generate the data under each scenario
     (stored in `/SCENARIOS`),
- 2. generate the datasets (stored in `/GENERATED_DATA`),
- 3. save the model parameters’ combinations (stored in
     `/SET_PARAMETERS`)
- 4. fit the models (complete outputs saved in
     `/ESTIMATED_RUNS`) and
- 5. extract the best runs and save the corresponding results in
     `.RDS` files (stored in `/EXTRACT_RESULTS`)

Running only one of the scripts listed in
reconstructs the simulation study only partially. All five scripts must
be run to reproduce all the simulation results reported in the main
*manuscript*.

```
source("SIMULATION_STUDIES/run_functions_Simulation_study.R")
source("SIMULATION_STUDIES/run_functions_Add_sim_study_1.R")
source("SIMULATION_STUDIES/run_functions_Add_sim_study_2.R")
source("SIMULATION_STUDIES/run_functions_Add_sim_study_3.R")
source("SIMULATION_STUDIES/run_functions_Add_sim_study_4.R")
```

### Section 2.3 - Simulation results

Use the R scripts `plot_*.R` to create the images reported
in the paper. The plots are saved in /GRAPHS. In particular,

- `plot_Simulation_graphs_Spatial.R` recreates all the
  figures related to the TRIFASE clustering performances under different
  experimental conditions;
- `plot_Simulation_graphs_noSpatial.R` recreates all the
  figures related to the TRIFASE clustering performances under the
  scenario without spatial correlation;
- `plot_Simulation_graphs_Scalability.R` investigates the
  scalability of the TRIFASE algorithm under different experimental
  conditions;
- `plot_Simulation_graphs_Loss.R` investigates the results
  in terms of the loss function of the TRIFASE algorithm;

In detail, Table 1 maps all the results
reported in the Manuscript with the corresponding generation code.

```
source("SIMULATION_STUDIES/plot_Simulation_graphs_Spatial.R")
source("SIMULATION_STUDIES/plot_Simulation_graphs_noSpatial.R")
source("SIMULATION_STUDIES/plot_Simulation_graphs_Scalability.R")
source("SIMULATION_STUDIES/plot_Simulation_graphs_Loss.R")
```

#### Table 1

| ID | Position | Object Directory | Original Script | Line in Original Script |
| --- | --- | --- | --- | --- |
| Figure2 | Main Manuscript | SIMULATION\_STUDIES/GRAPHS/ | plot\_Simulation\_graphs\_Spatial.R | 997 |
| Figure3 | Main Manuscript | SIMULATION\_STUDIES/GRAPHS/ | plot\_Simulation\_graphs\_Spatial.R | 988 |
| Figure4 | Main Manuscript | SIMULATION\_STUDIES/GRAPHS/ | plot\_Simulation\_graphs\_Spatial.R | 971 |
| Figure5 | Main Manuscript | REAL\_DATA/GRAPHS/ | Real\_Data\_graph.R | 65 |
| Figure6 | Main Manuscript | REAL\_DATA/GRAPHS/ | Real\_Data\_graph.R | 172 |
| Table1 | Main Manuscript | REAL\_DATA/GRAPHS/ | Real\_Data\_analysis.R | 95 |
| Figure2Suppl | Supporting Information | SIMULATION\_STUDIES/GRAPHS/ | plot\_Simulation\_graphs\_Spatial.R | 1001 |
| Figure3Suppl | Supporting Information | SIMULATION\_STUDIES/GRAPHS/ | plot\_Simulation\_graphs\_Spatial.R | 1005 |
| Figure4Suppl | Supporting Information | SIMULATION\_STUDIES/GRAPHS/ | plot\_Simulation\_graphs\_Spatial.R | 992 |
| Figure5Suppl | Supporting Information | SIMULATION\_STUDIES/GRAPHS/ | plot\_Simulation\_graphs\_Scalability.R | 89 |
| Figure6Suppl | Supporting Information | SIMULATION\_STUDIES/GRAPHS/ | plot\_Simulation\_graphs\_Spatial.R | 975 |
| Figure7Suppl | Supporting Information | SIMULATION\_STUDIES/GRAPHS/ | plot\_Simulation\_graphs\_Spatial.R | 979 |
| Figure8Suppl | Supporting Information | SIMULATION\_STUDIES/GRAPHS/ | plot\_Simulation\_graphs\_Spatial.R | 983 |
| Figure9Suppl | Supporting Information | SIMULATION\_STUDIES/GRAPHS/ | plot\_Simulation\_graphs\_Spatial.R | 966 |
| Figure10Suppl | Supporting Information | SIMULATION\_STUDIES/GRAPHS/ | plot\_Simulation\_graphs\_noSpatial.R | 351 |
| Figure11Suppl | Supporting Information | SIMULATION\_STUDIES/GRAPHS/ | plot\_Simulation\_graphs\_noSpatial.R | 355 |
| Figure12Suppl | Supporting Information | SIMULATION\_STUDIES/GRAPHS/ | plot\_Simulation\_graphs\_noSpatial.R | 359 |
| Figure13Suppl | Supporting Information | SIMULATION\_STUDIES/GRAPHS/ | plot\_Simulation\_graphs\_Loss.R | 210 |
| Figure14Suppl | Supporting Information | REAL\_DATA/GRAPHS/ | Real\_Data\_graph.R | 132 |
| Figure15Suppl | Supporting Information | REAL\_DATA/GRAPHS/ | Real\_Data\_graph.R | 146 |

### Section 2.4 - Master R script

All the scripts described in Section 2 - subsection
2 and Section 2 - subsection 3 are reported in
a unique R file called `master.R`. This script will reproduce
all the results related to the simulations. Be sure to set the parent
directory `TRIFASE_Code` as the working directory before
running `master.R`.

For each Figure and Table generated through `master.R`,
the object names in the R script match the exact labels in the
Manuscript. E.g., Figure 1 corresponds to the `Figure1`
object in the `R script`, and Supplementary Figure 1 is
labeled as `Figure1Suppl`. Users can locate the line numbers
in `master.R` where Figures and Tables are saved by referring
to Table 1.

By default, the script utilizes 60 cores. To adjust the number of
cores, modify `n.cores` (line 30).

# Section 3

The data that support the findings of this study originated from:

- *V. Denti, G. Capitoli, I. Piga, F. Clerici, L. Pagani, L.
  Criscuolo, G. Bindi, L. Principi, C. Chinello, G. Paglia, F. Magni, A.
  Smith*  
  Spatial Multiomics of Lipids, N-Glycans, and Tryptic Peptides on a
  Single FFPE Tissue Section  
  **Journal of Proteome Research**, 21(11):2798–2809, 2022.
  ISSN 1535-3907. doi: 10.1021/acs.jproteome.2c00601.571

### Real data results

The scripts stored in the folder `TRIFASE_Code` will
reproduce all the results related to the real data (i.e., the mouse
brain tissue sample). To map Tables and Figures from
`Real Data codes` to the Manuscript text we refer to Table 1.

To reproduce the Real Data results, users need to request the
`X.RDS` and the `S.RDS` from the corresponding
author. The dataset (`X.RDS`) comprises 78 rows representing
the lipid signals observed over approximately 6500 columns representing
the spatially related pixels. Spatial locations are stored in the
`S.RDS` file. Data are available from the corresponding
author upon reasonable request.

Once data are available, set the parent directory
`TRIFASE_Code` as the working directory. Make sure to save
the `X.RDS` and the `S.RDS` in the
`DATASET` folder.  
The function needed for the real data study can be imported into the R
Environment:

```
source("REAL_DATA/c_Estimate_RealData.R")
```

Without manipulations the script `Real_Data_analysis.R`
fits TRIFASE to the real dataset. The model is fitted under different
random starting points, retaining the model estimate corresponding to
the smallest value of the loss function. To assess the strength of the
spatial correlation, the script fits sixteen different TRIFASE models
characterized by different fixed values of \(\phi \in \{0.5,0.7,0.9,1,3,5,10,20\}\) and
by the two fastest versions of TRIFASE (C,A) and (S,A).

```
source("REAL_DATA/Real_Data_analysis.R")
```

The different conditions under which the estimation algorithms of
TRIFASE are run are displayed in Table 1 of the main Manuscript (to map
the Table from code to the Manuscript, we refer to Table 1).

The script `Real_Data_graph.R` reproduces all the Main and
Supplementary text figures using the model estimate corresponding to the
smallest loss function value. We refer to Table 1
to map Figures from the code to the text.

```
source("REAL_DATA/Real_Data_graph.R")
```

# Section 4

Another file in the folder `TRIFASE_Code`:

- `README.Rmd` produce this `.html`.
