# Supplementary material for: Spatially Informed Nonnegative Matrix Trifactorization for Coclustering Mass Spectrometry Data
Source: Biom J. 2025 Mar 19;67(2):e70031. doi: 10.1002/bimj.70031 (PMC11921289; doi:10.1002/bimj.70031)

Estimated non−empty row clusters (p=100)

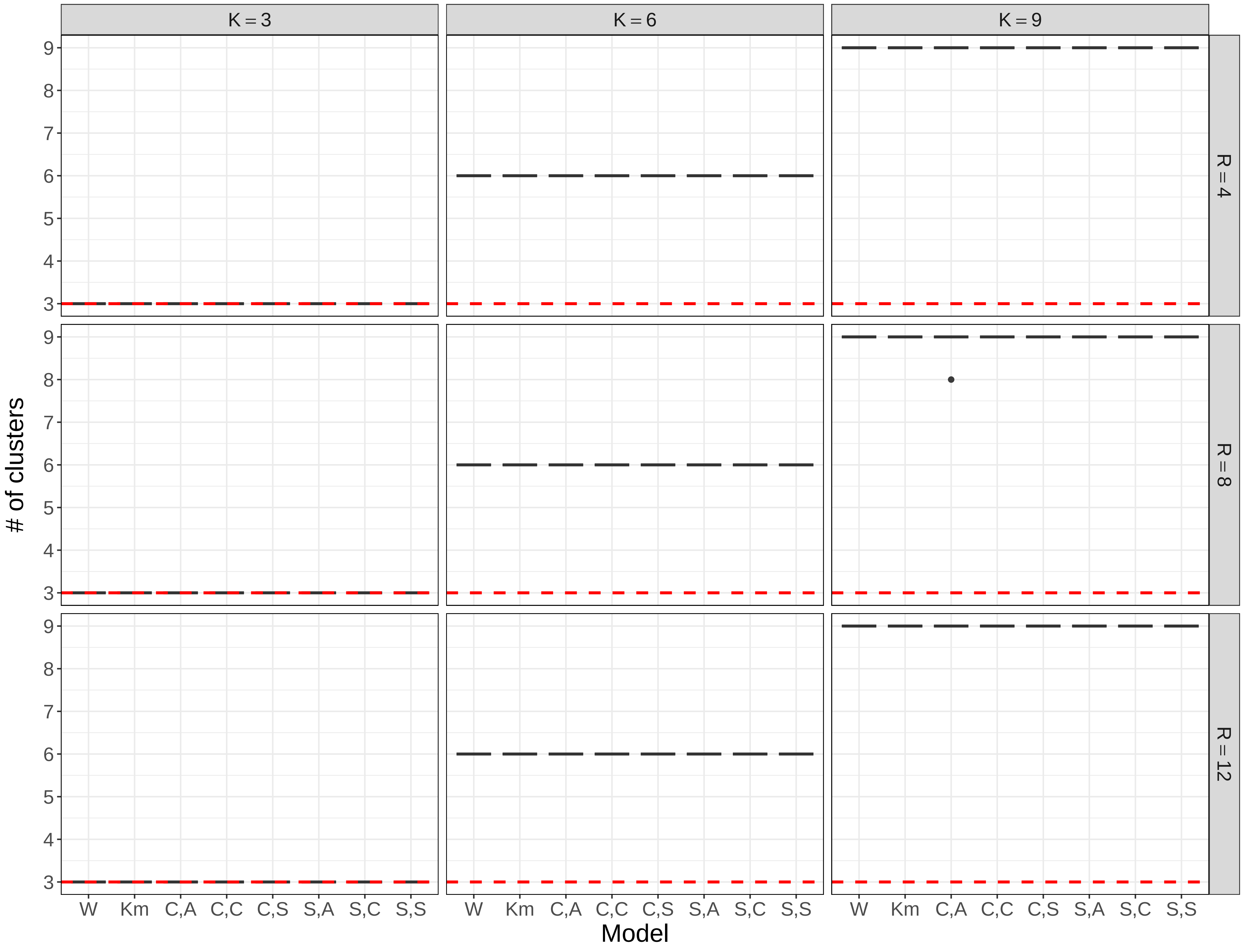

Estimated non−empty row clusters (p=1000)

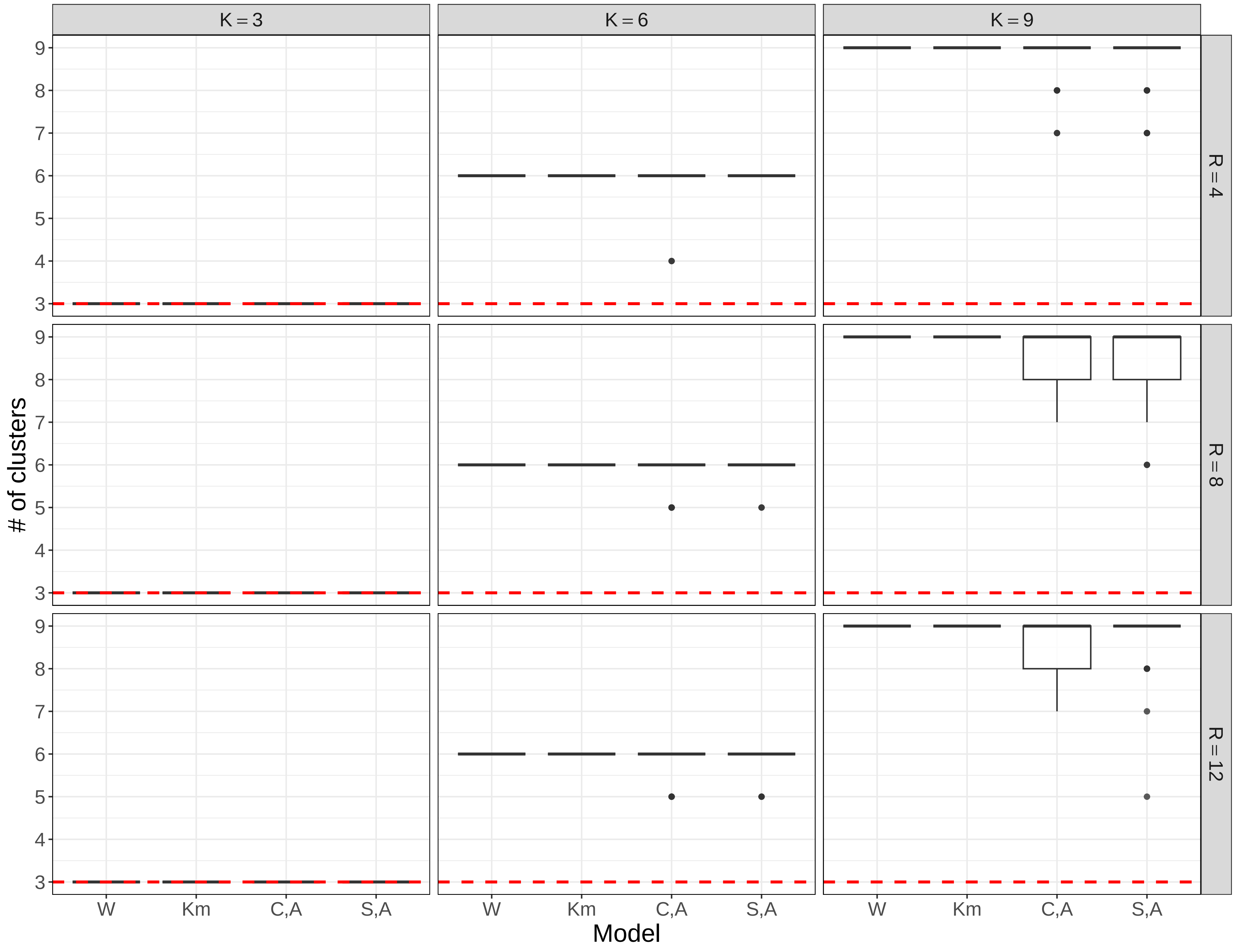

Estimated non−empty column clusters (p=100)

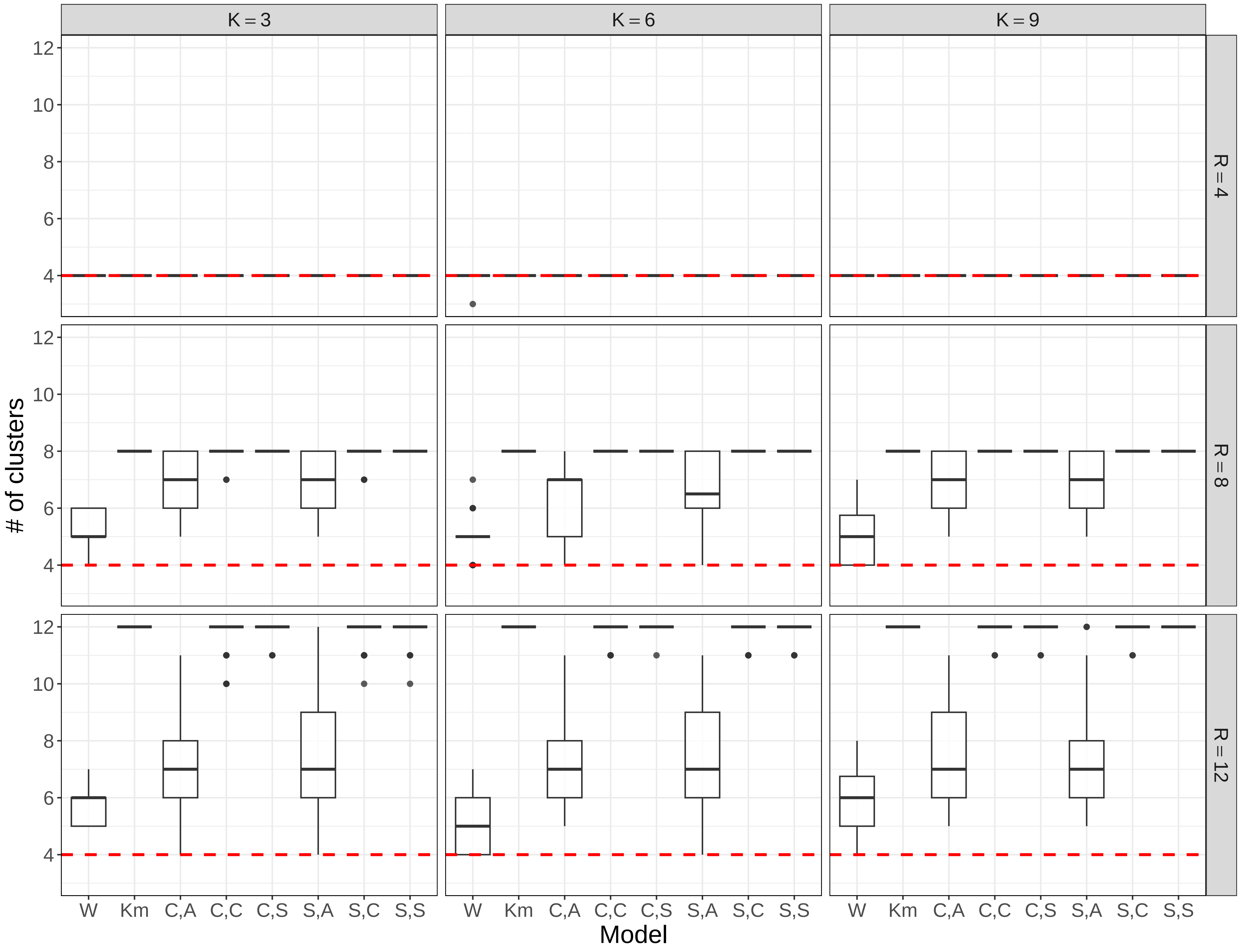

Estimated non−empty column clusters (p=1000)

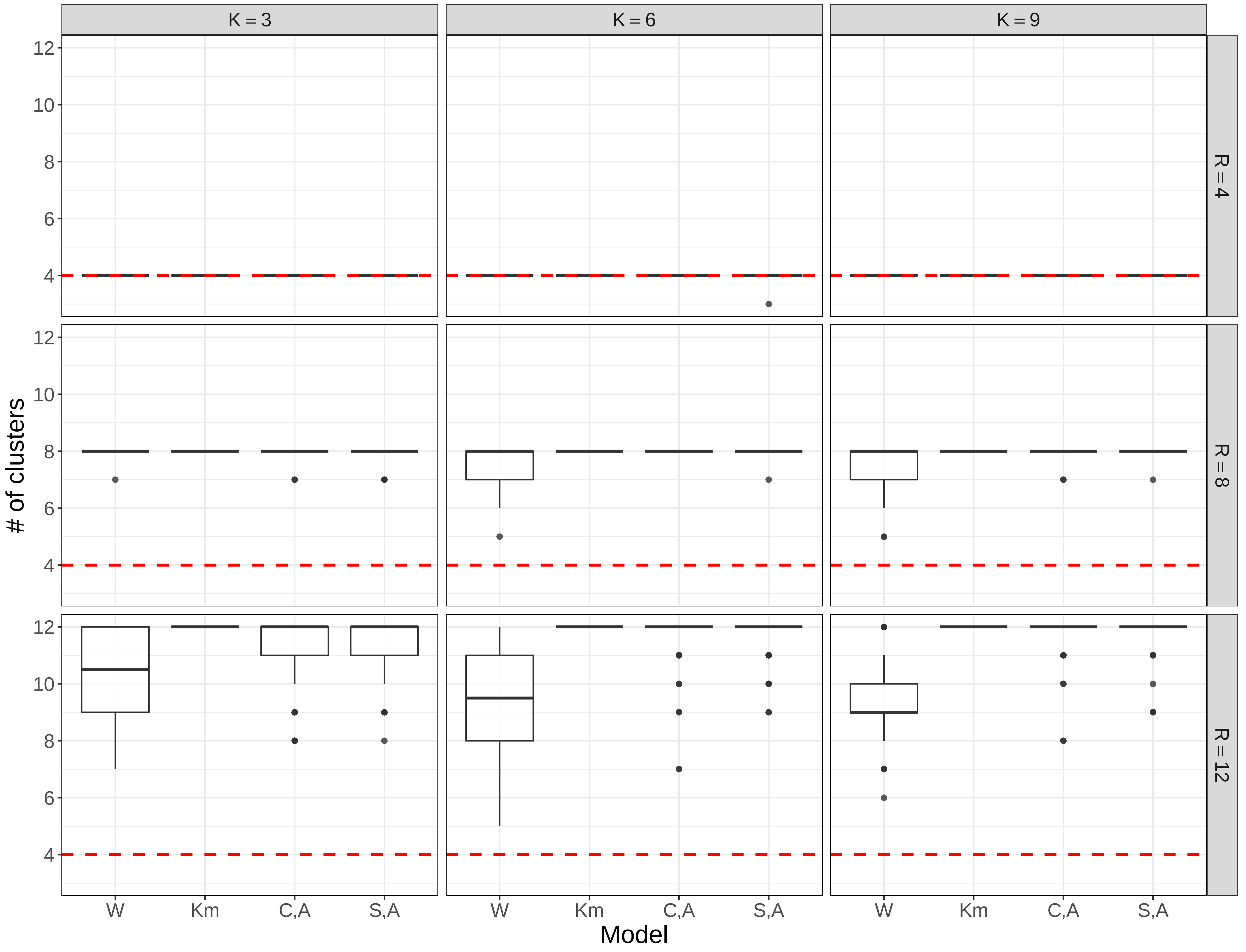

Supplement: Supplementary file 1 — Supporting Information [file BIMJ-67-e70031-s001.zip › TRIFASE_Code/SIMULATION_STUDIES/GRAPHS/Figure8Suppl.pdf]
