# Supplementary material for: Spatially Informed Nonnegative Matrix Trifactorization for Coclustering Mass Spectrometry Data
Source: Biom J. 2025 Mar 19;67(2):e70031. doi: 10.1002/bimj.70031 (PMC11921289; doi:10.1002/bimj.70031)
Supplement: Supplementary file 1 — Supporting Information [file BIMJ-67-e70031-s001.zip › TRIFASE_Code/SIMULATION_STUDIES/GRAPHS/Figure3Suppl.pdf]

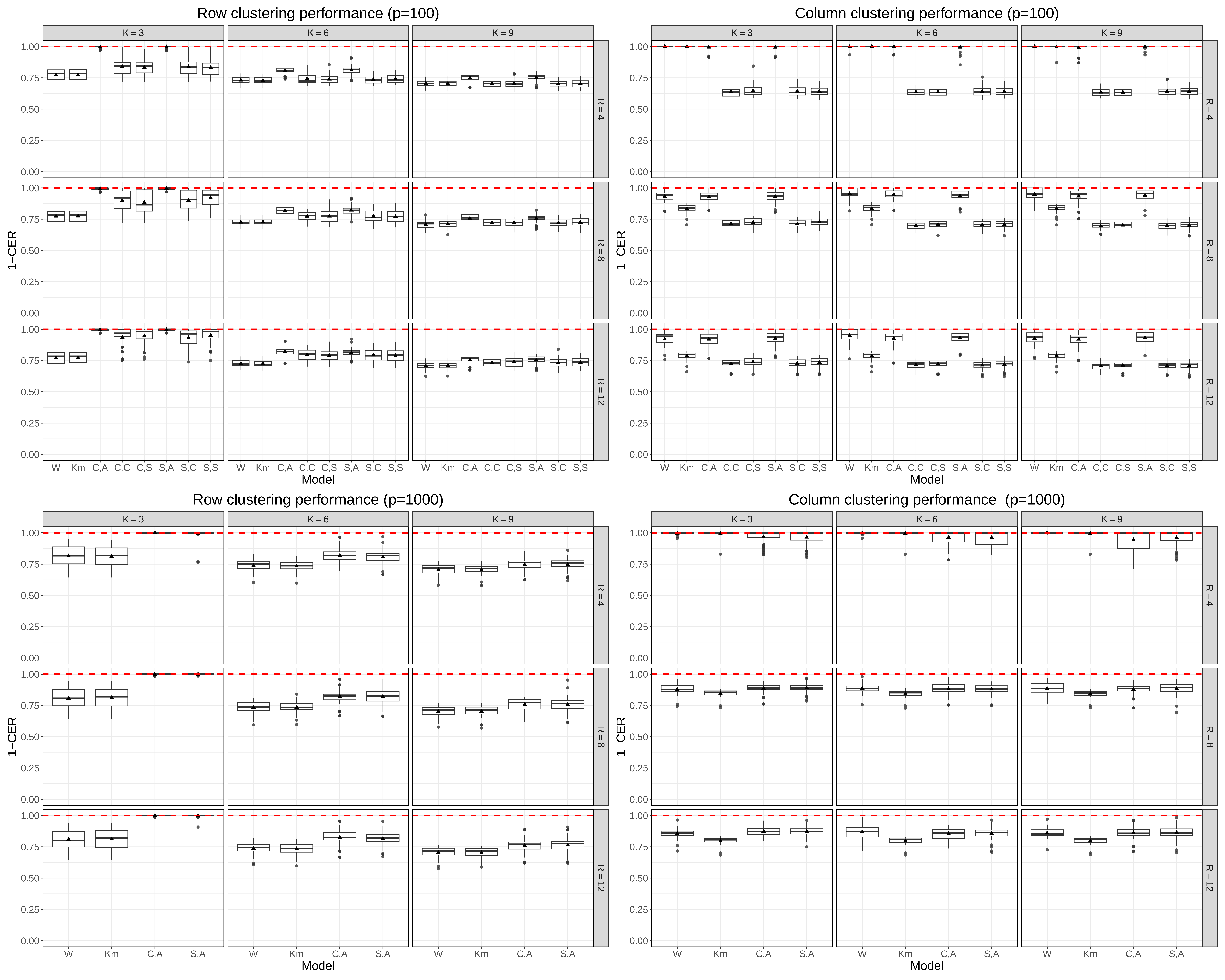

Row clustering performance (p=100)
Column clustering performance (p=100)
Row clustering performance (p=1000)
Column clustering performance (p=1000)
K = 3
K = 6
K = 9
R = 4
R = 8
R = 12
1−CER
1.00
0.75
0.50
0.25
0.00
W
Km
C,A
C,C
C,S
S,A
S,C
S,S
Model
