# Supplementary figures and images for: Spatially Informed Nonnegative Matrix Trifactorization for Coclustering Mass Spectrometry Data
Source: Biom J. 2025 Mar 19;67(2):e70031. doi: 10.1002/bimj.70031 (PMC11921289; doi:10.1002/bimj.70031)

Estimated non-empty row clusters (p=100)

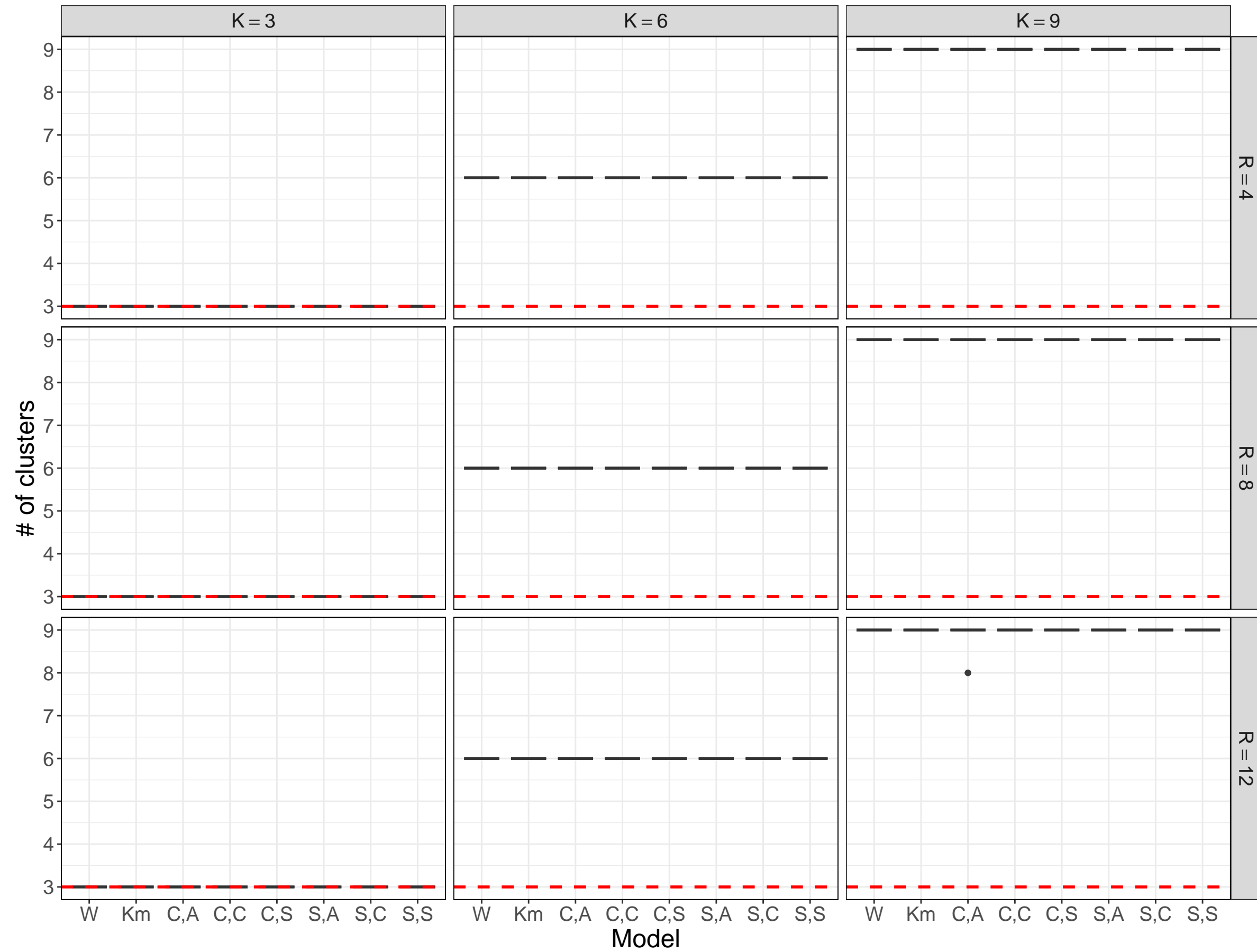

Estimated non-empty row clusters (p=1000)

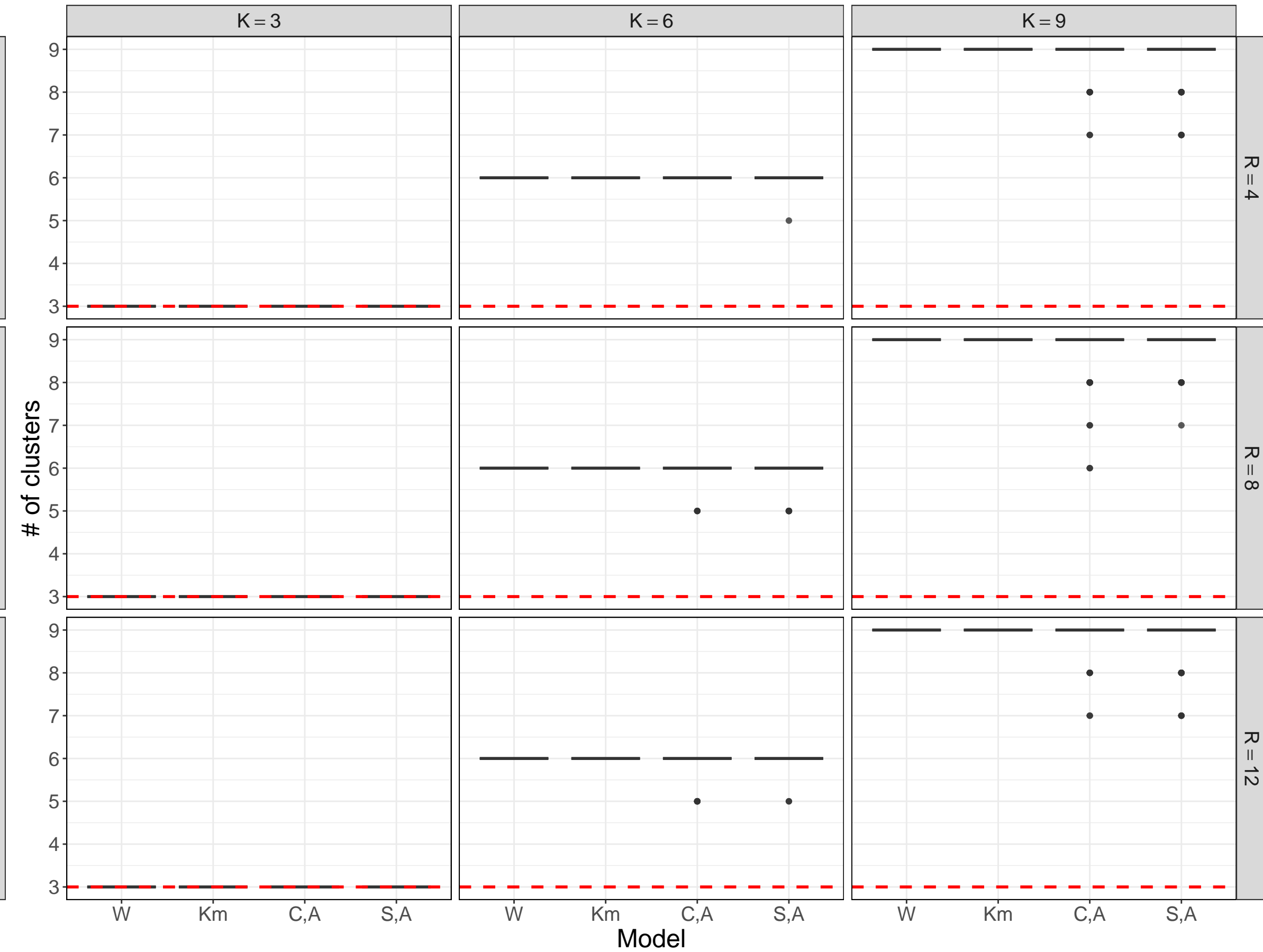

Supplement: Supplementary file 1 — Supporting Information [file BIMJ-67-e70031-s001.zip › TRIFASE_Code/SIMULATION_STUDIES/GRAPHS/Figure6Suppl.pdf]

Loss function values across 50 runs (p=100)

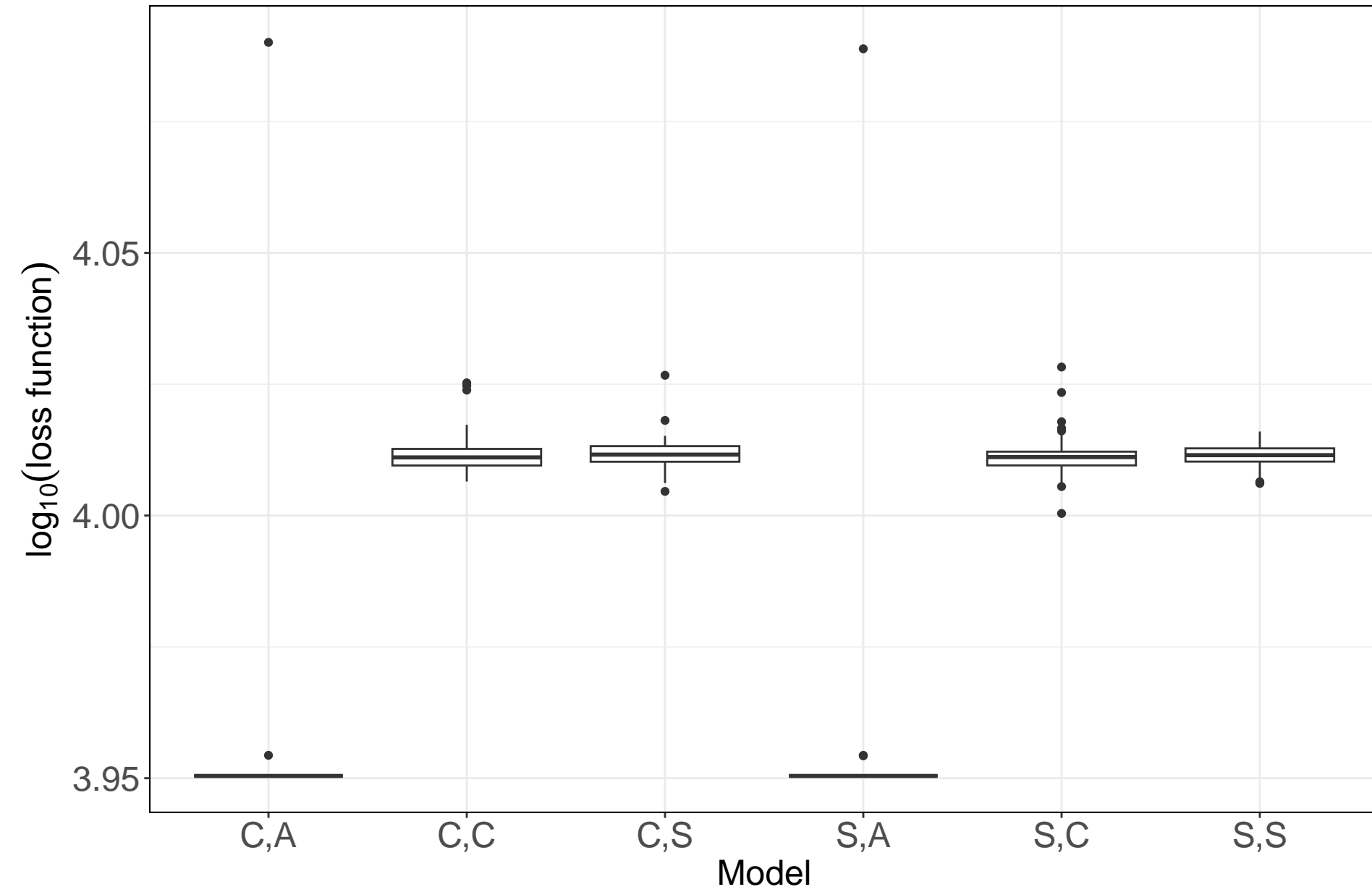

Loss function values across 50 runs (p=1000)

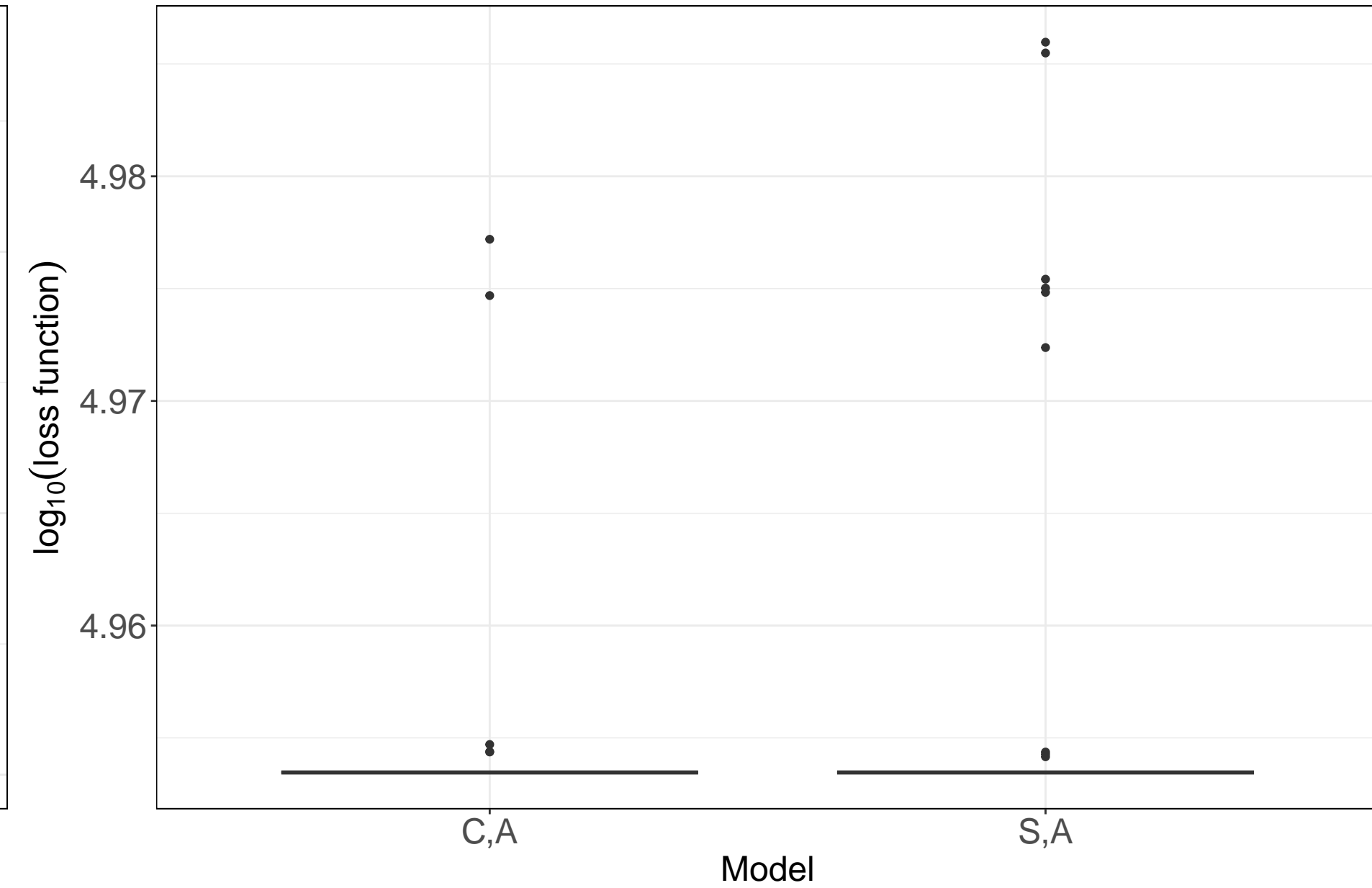

Supplement: Supplementary file 1 — Supporting Information [file BIMJ-67-e70031-s001.zip › TRIFASE_Code/SIMULATION_STUDIES/GRAPHS/Figure13ASuppl.pdf]

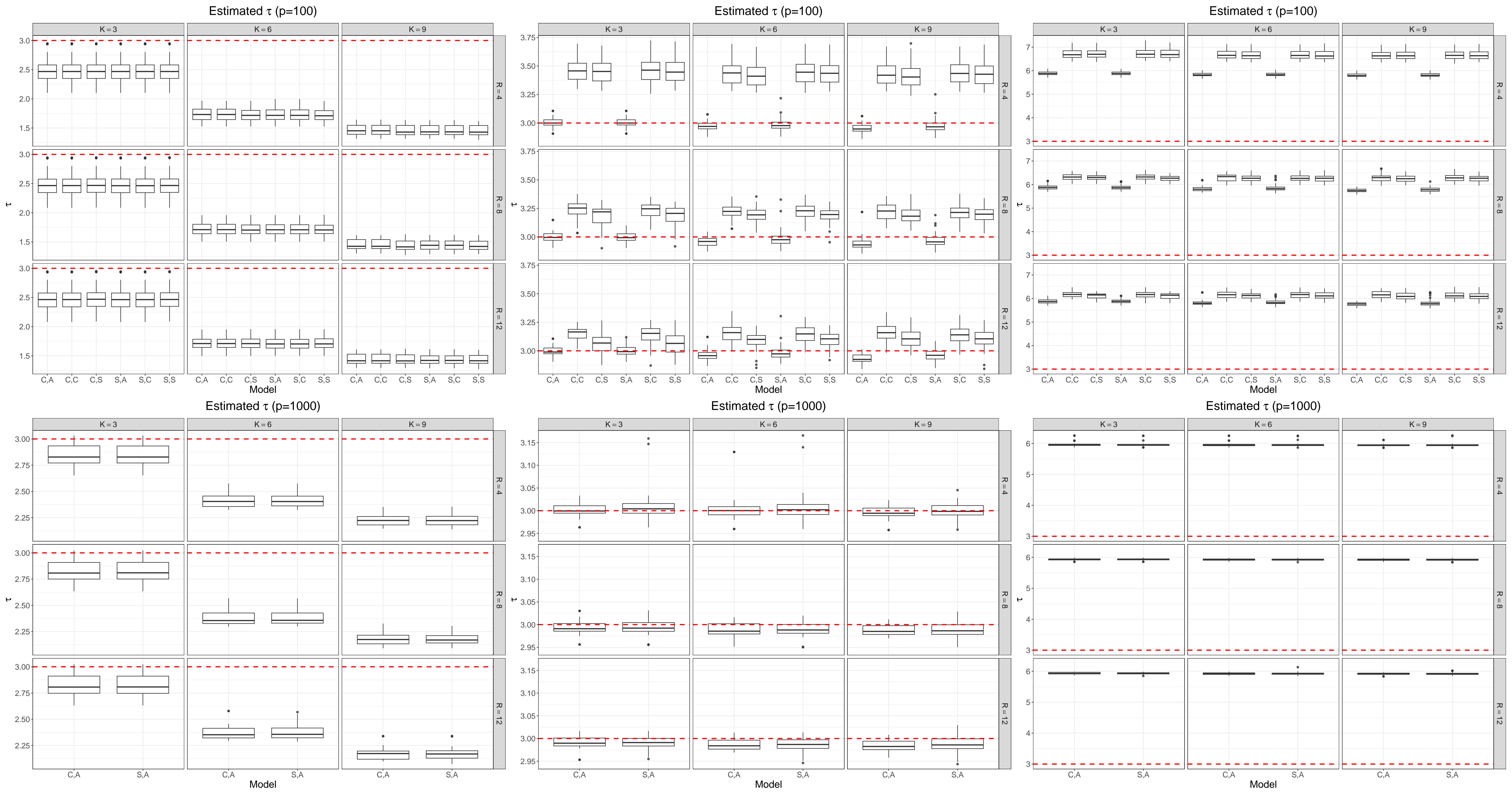

Supplement: Supplementary file 1 — Supporting Information [file BIMJ-67-e70031-s001.zip › TRIFASE_Code/SIMULATION_STUDIES/GRAPHS/Figure9Suppl.pdf]

Row clustering comparison over 50 runs (p=1000)

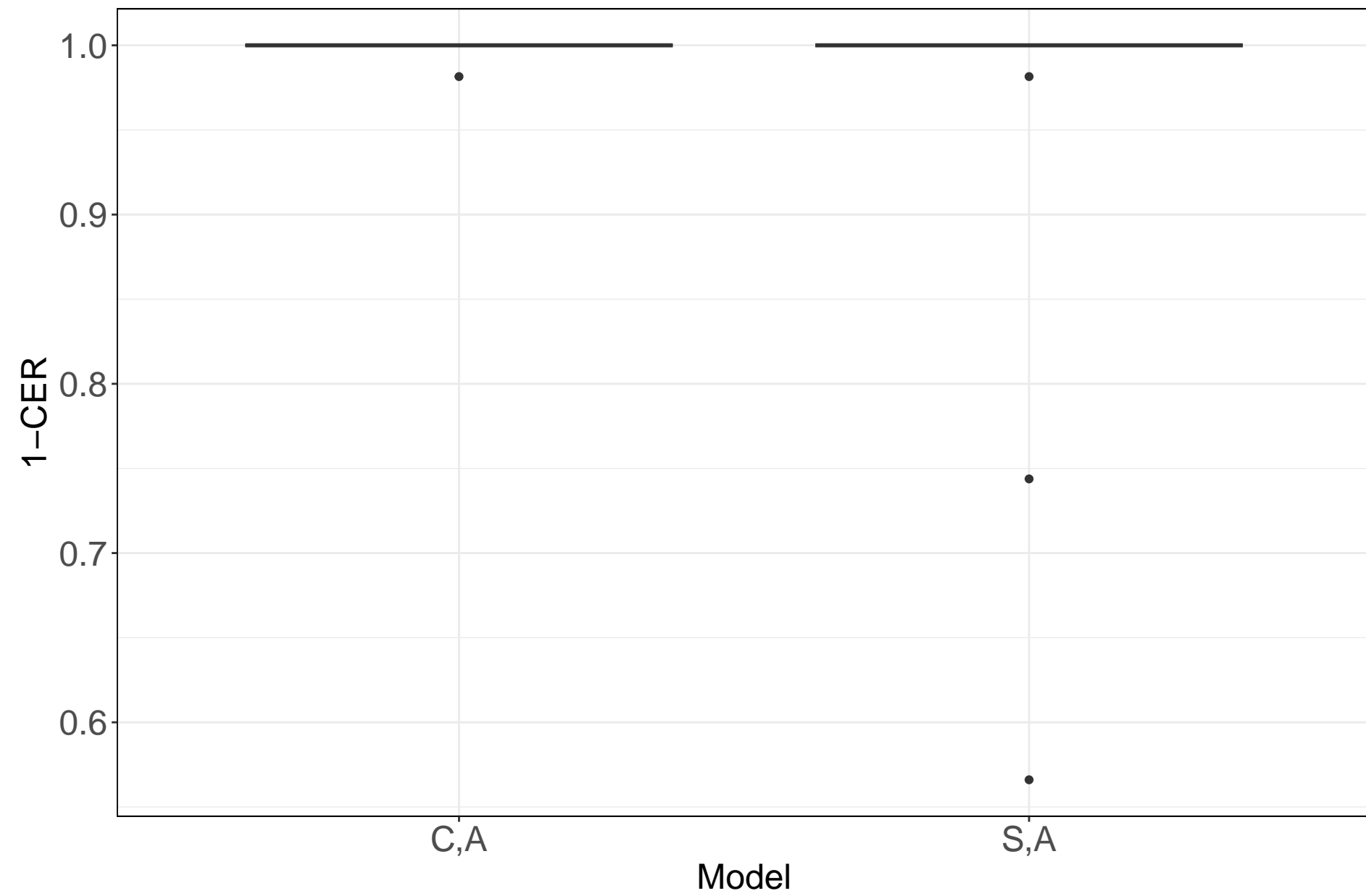

Column clustering comparison over 50 runs (p=1000)

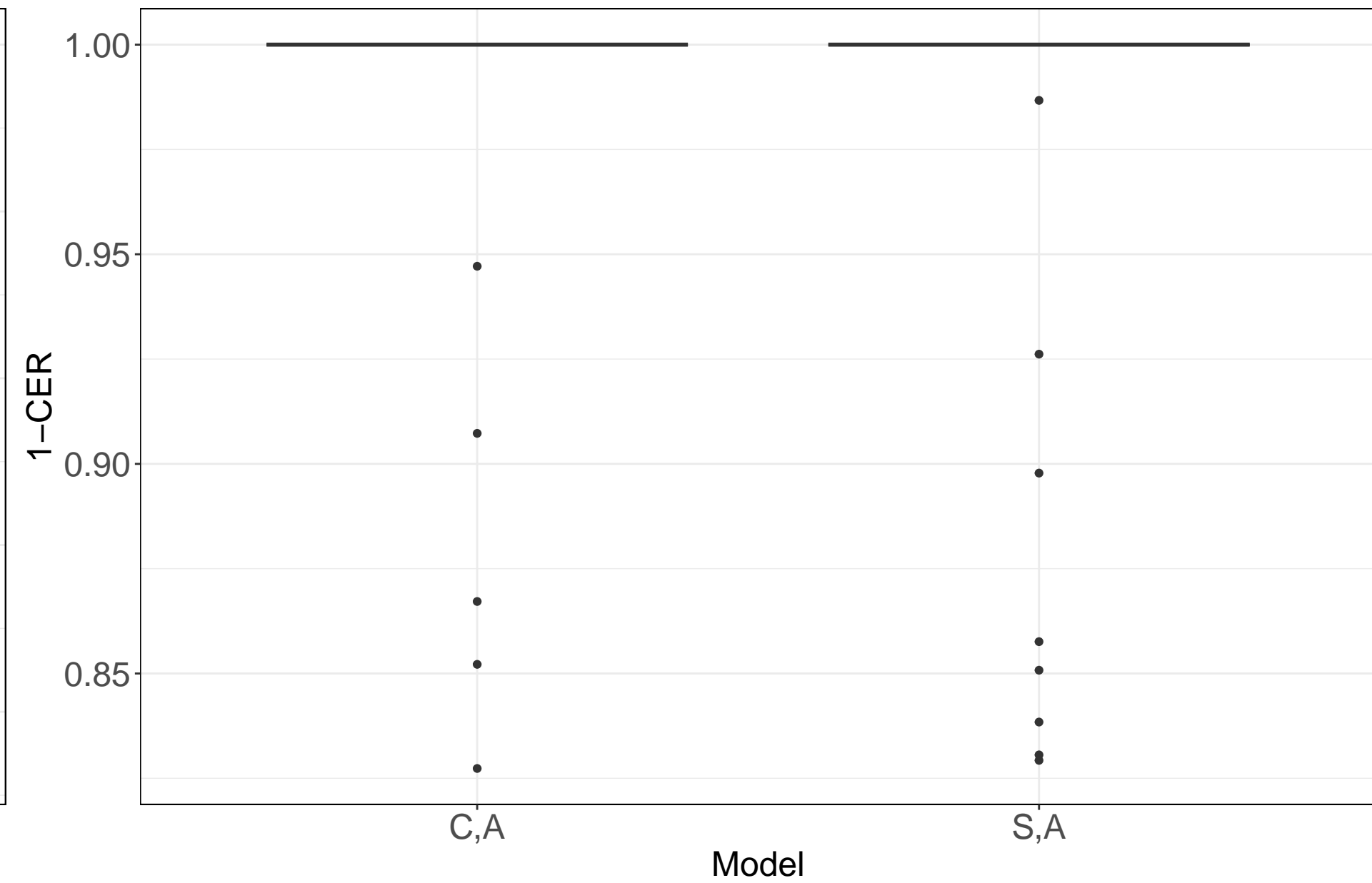

Supplement: Supplementary file 1 — Supporting Information [file BIMJ-67-e70031-s001.zip › TRIFASE_Code/SIMULATION_STUDIES/GRAPHS/Figure13CSuppl.pdf]

Row clustering comparison over 50 runs (p=100)

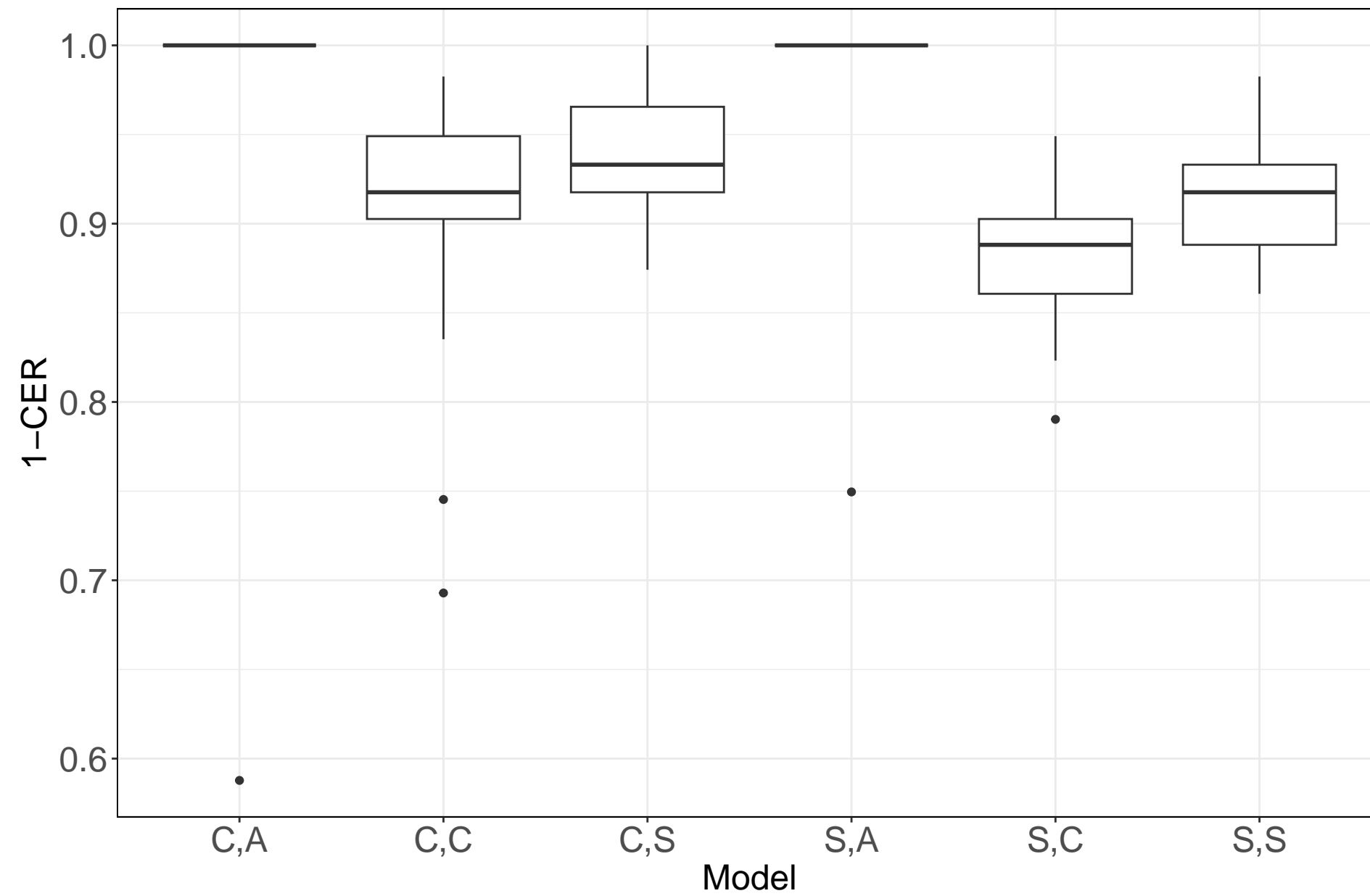

Column clustering comparison over 50 runs (p=100)

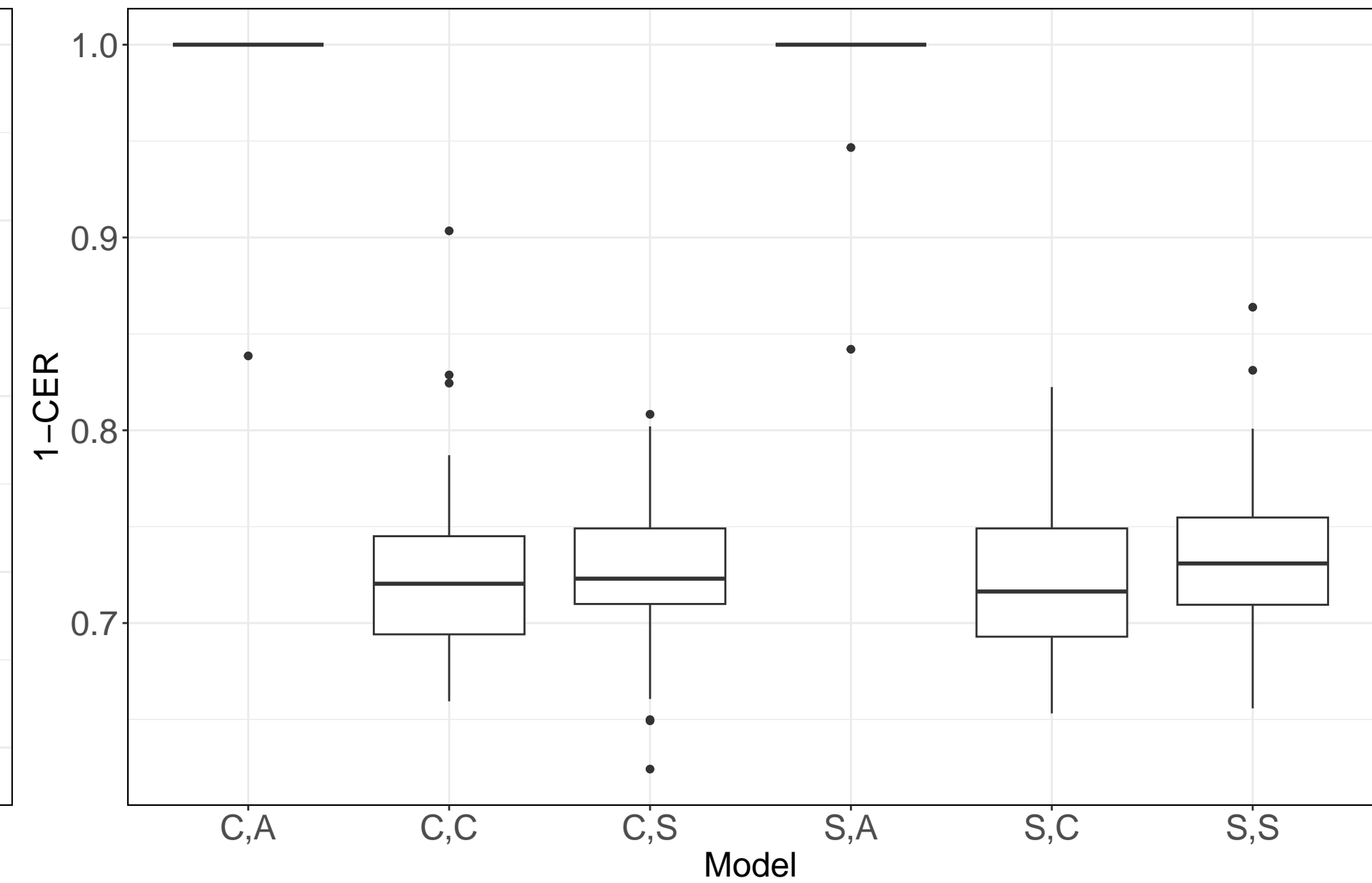

Supplement: Supplementary file 1 — Supporting Information [file BIMJ-67-e70031-s001.zip › TRIFASE_Code/SIMULATION_STUDIES/GRAPHS/Figure13BSuppl.pdf]

Seconds

K = 3

K = 9

R = 4

R = 12

Number of columns

Number of rows — 150 — 750 — 1500

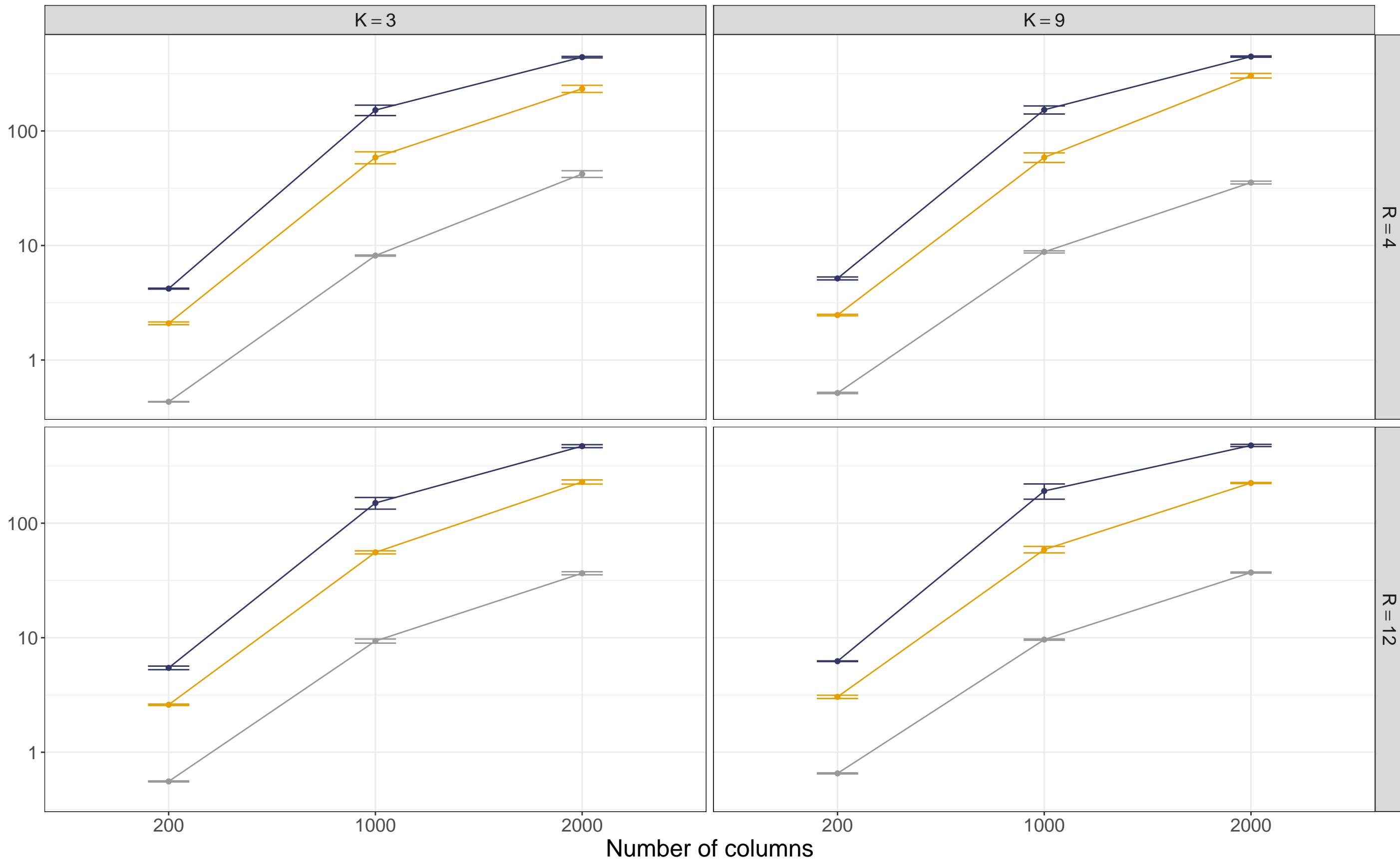

Supplement: Supplementary file 1 — Supporting Information [file BIMJ-67-e70031-s001.zip › TRIFASE_Code/SIMULATION_STUDIES/GRAPHS/Figure5Suppl.pdf]

Computational time (p=100)

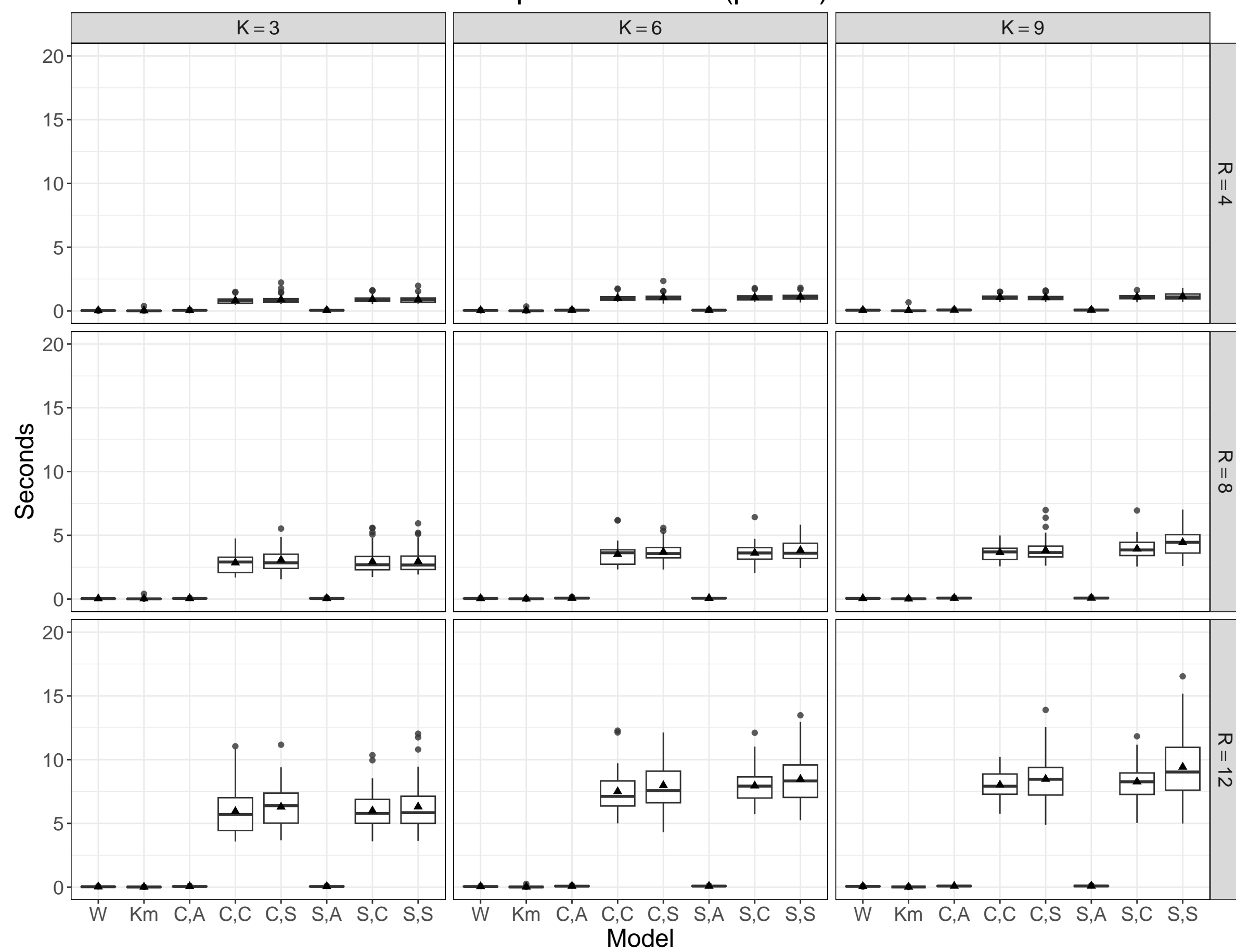

Computational time (p=1000)

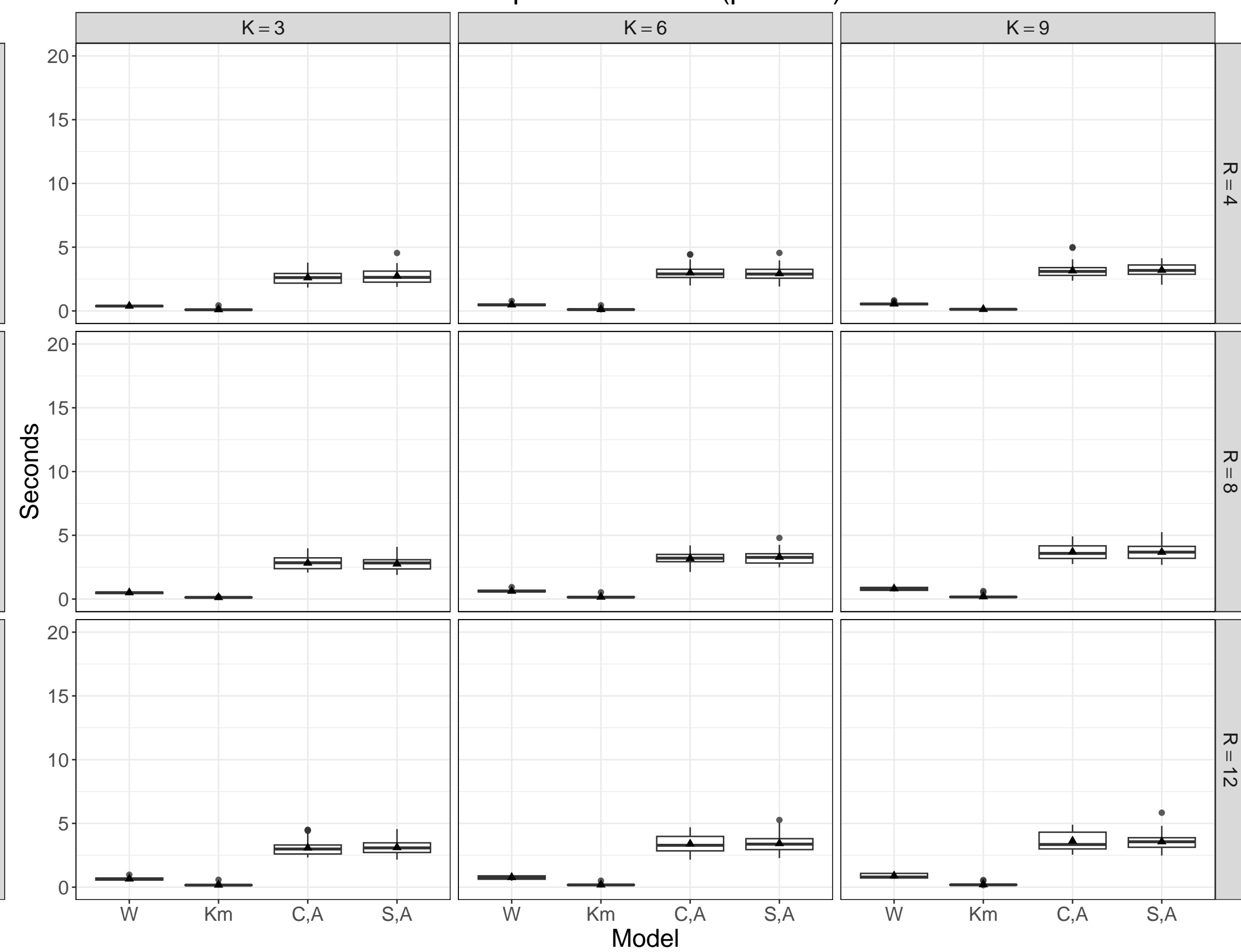

Computational time (p=100)

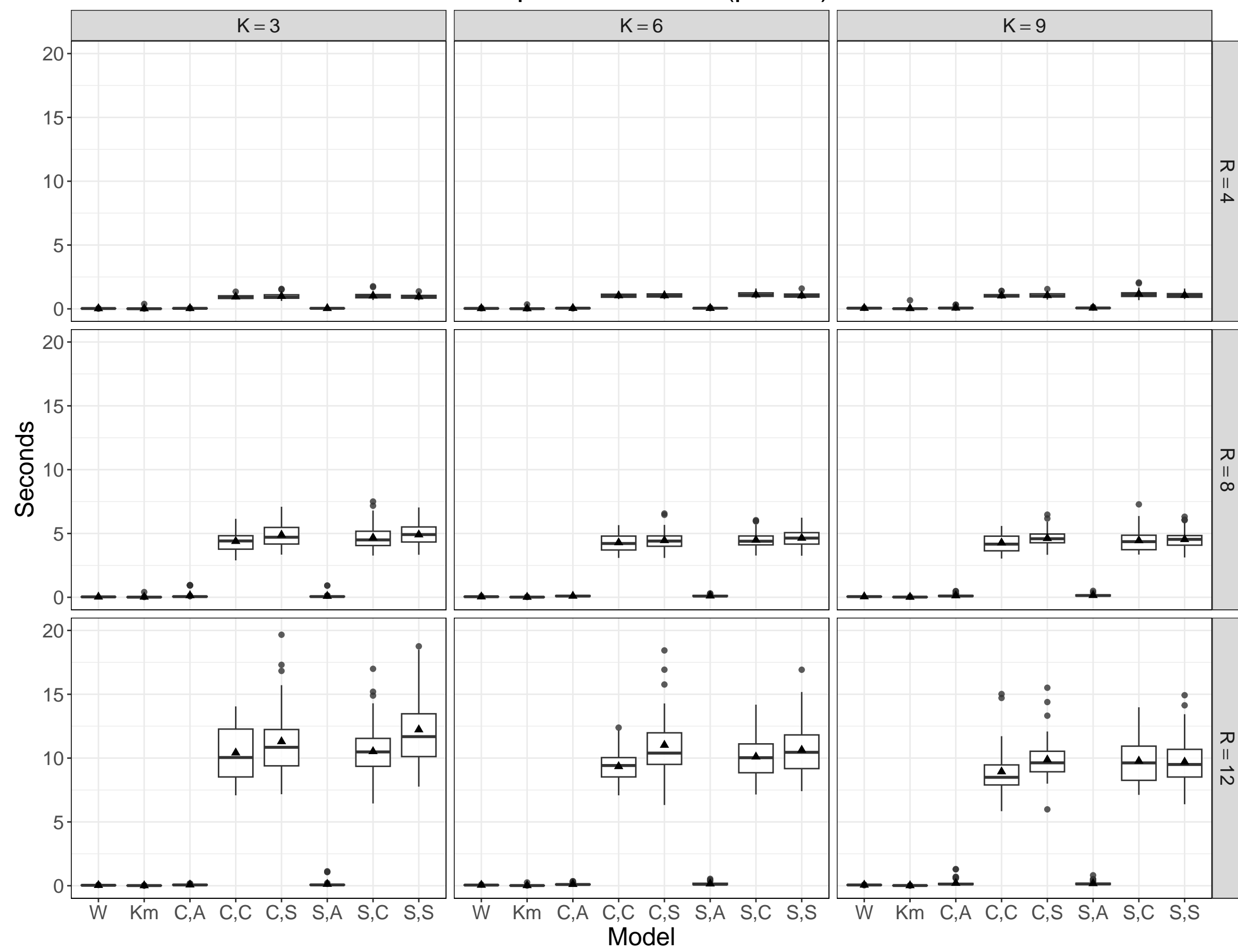

Computational time (p=1000)

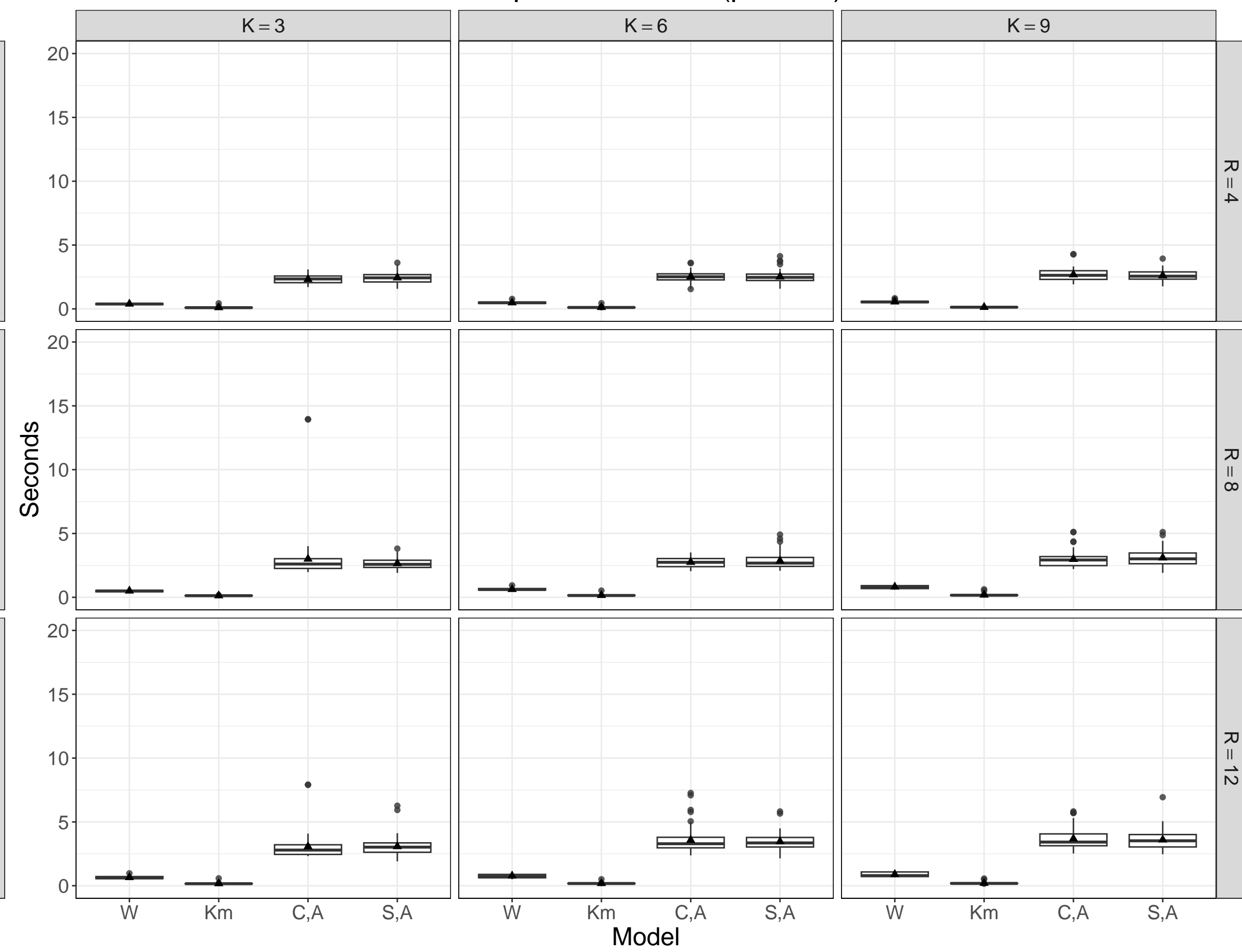

Supplement: Supplementary file 1 — Supporting Information [file BIMJ-67-e70031-s001.zip › TRIFASE_Code/SIMULATION_STUDIES/GRAPHS/Figure4Suppl.pdf]

### Computational time (p=100)

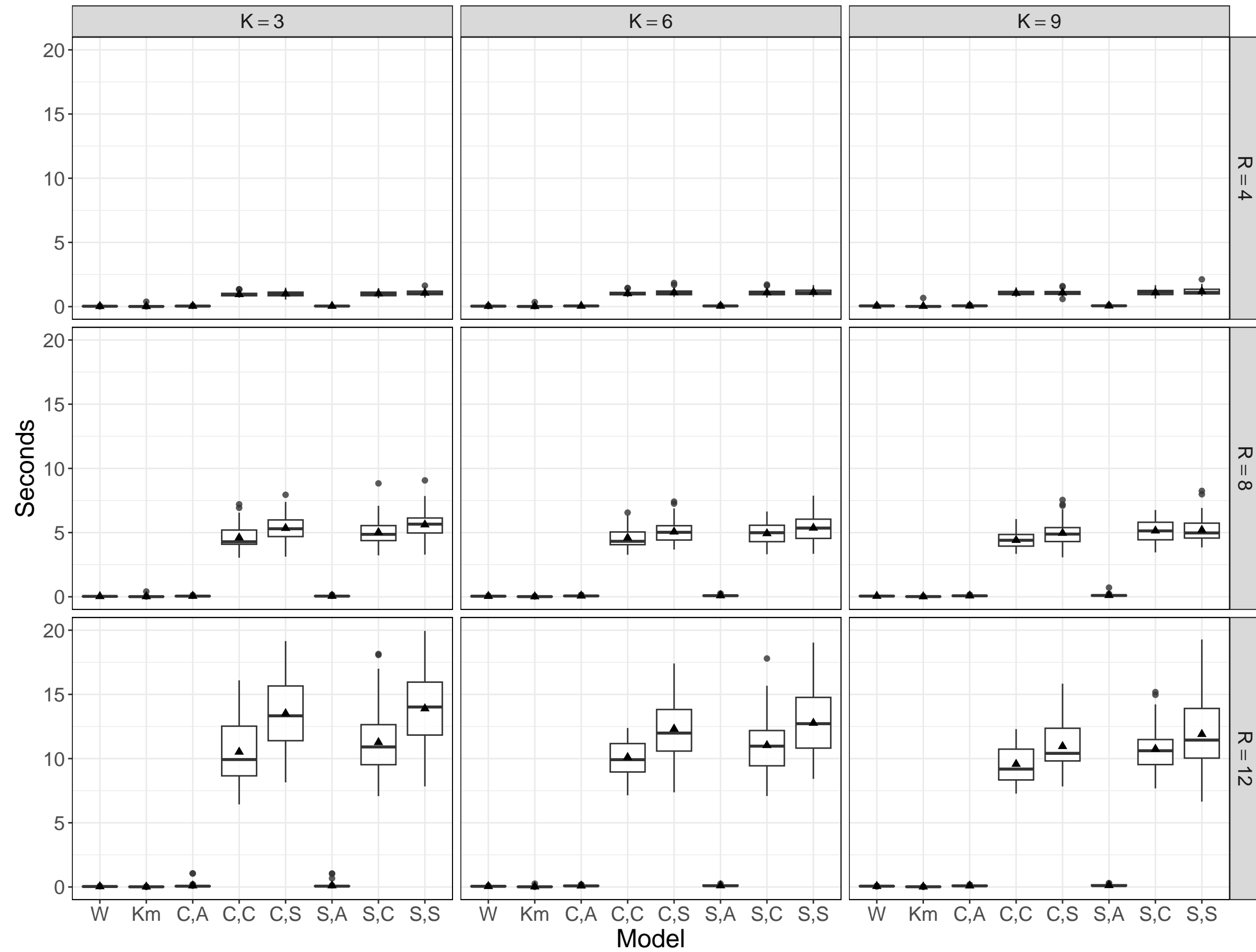

### Computational time (p=1000)

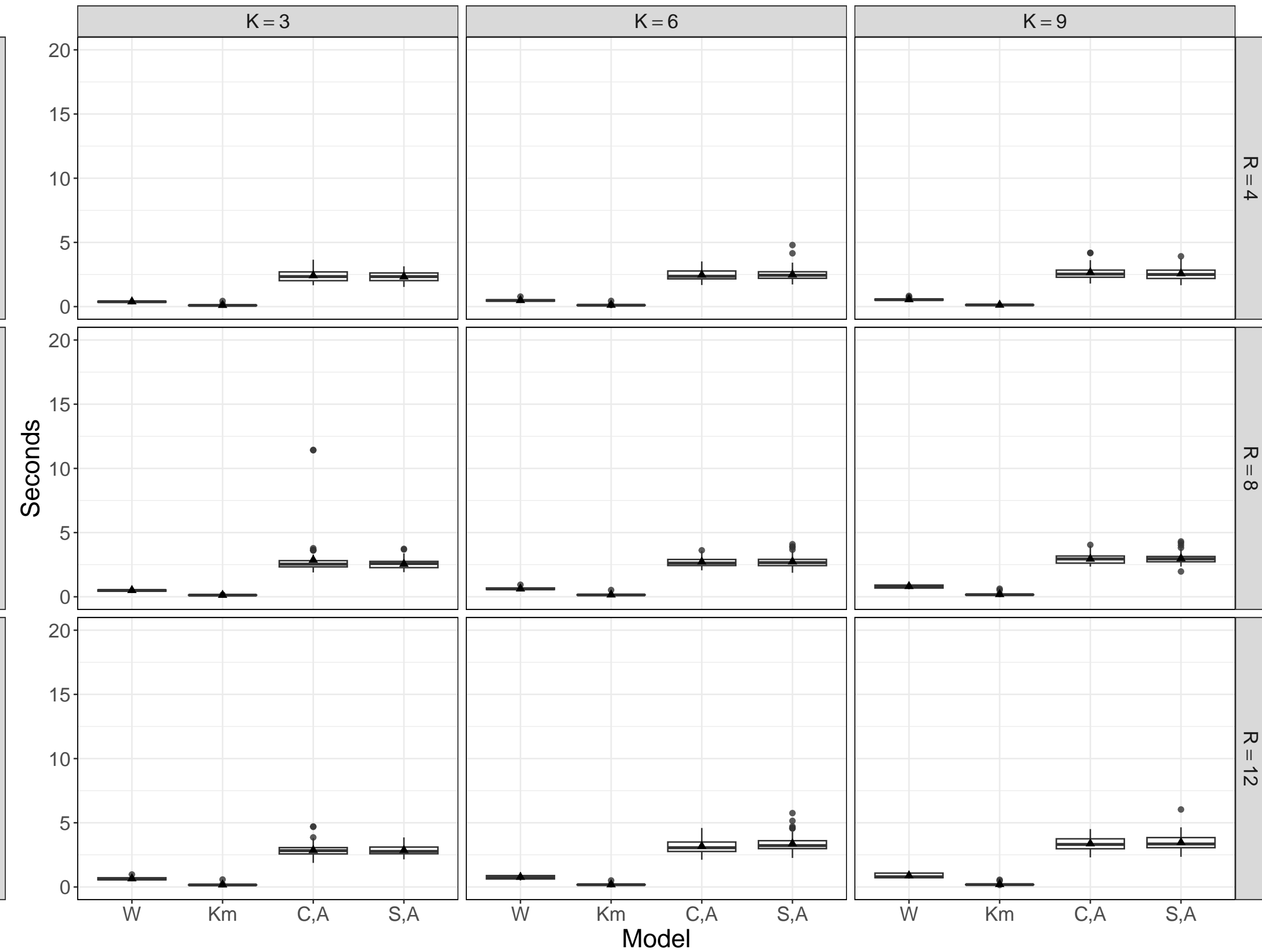

Supplement: Supplementary file 1 — Supporting Information [file BIMJ-67-e70031-s001.zip › TRIFASE_Code/SIMULATION_STUDIES/GRAPHS/Figure3.pdf]

Estimated non-empty column clusters (p=100)

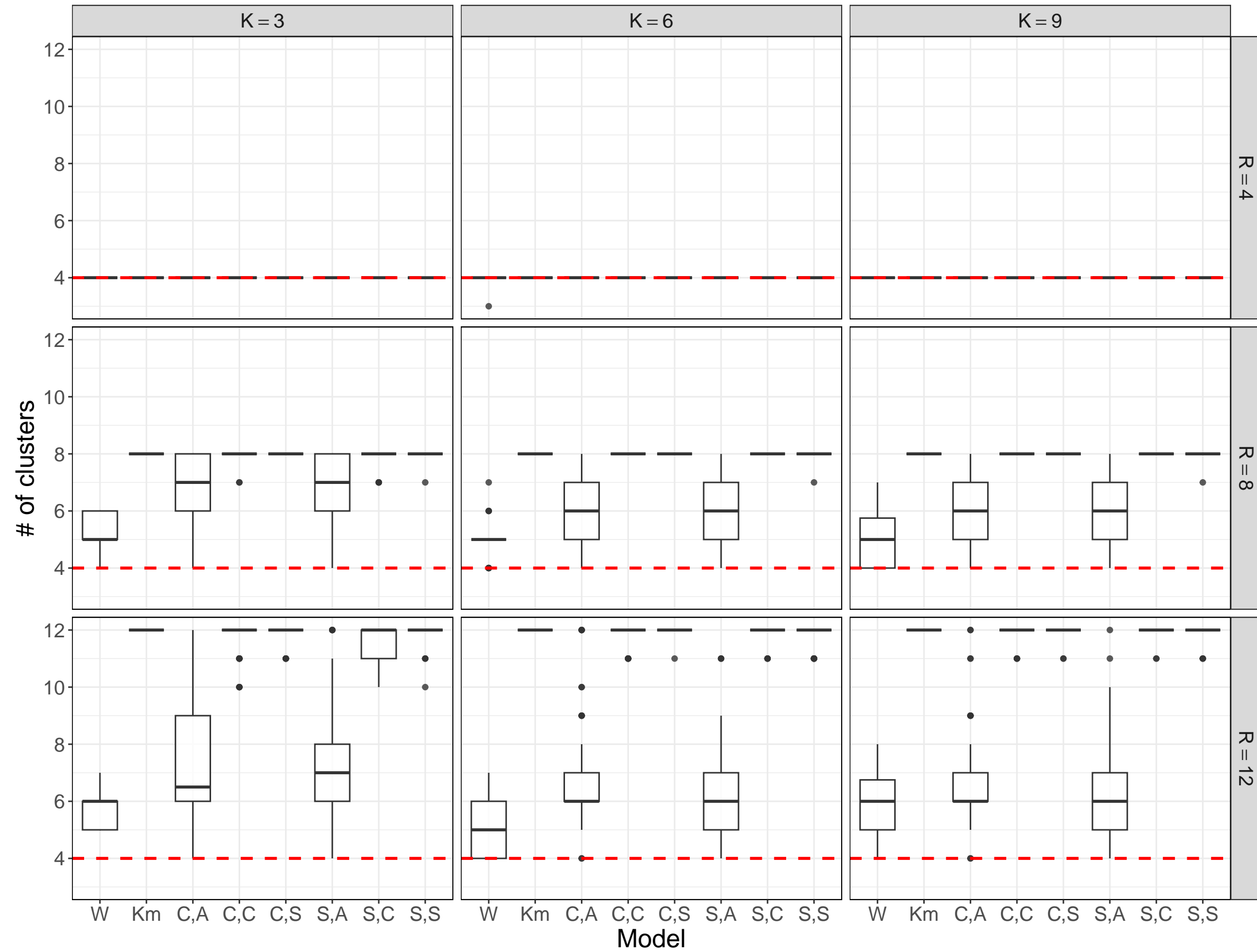

Estimated non-empty column clusters (p=1000)

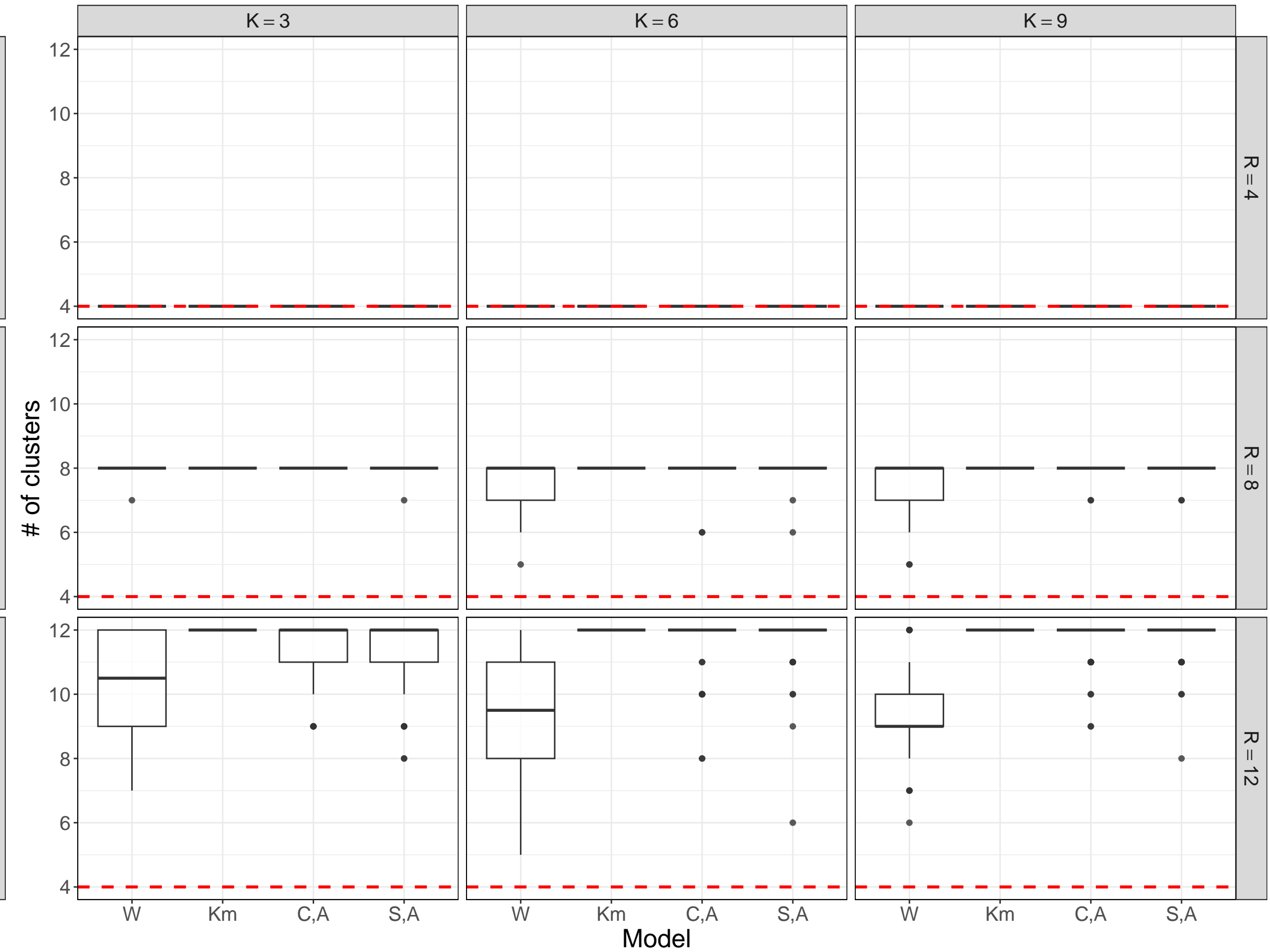

Supplement: Supplementary file 1 — Supporting Information [file BIMJ-67-e70031-s001.zip › TRIFASE_Code/SIMULATION_STUDIES/GRAPHS/Figure4.pdf]

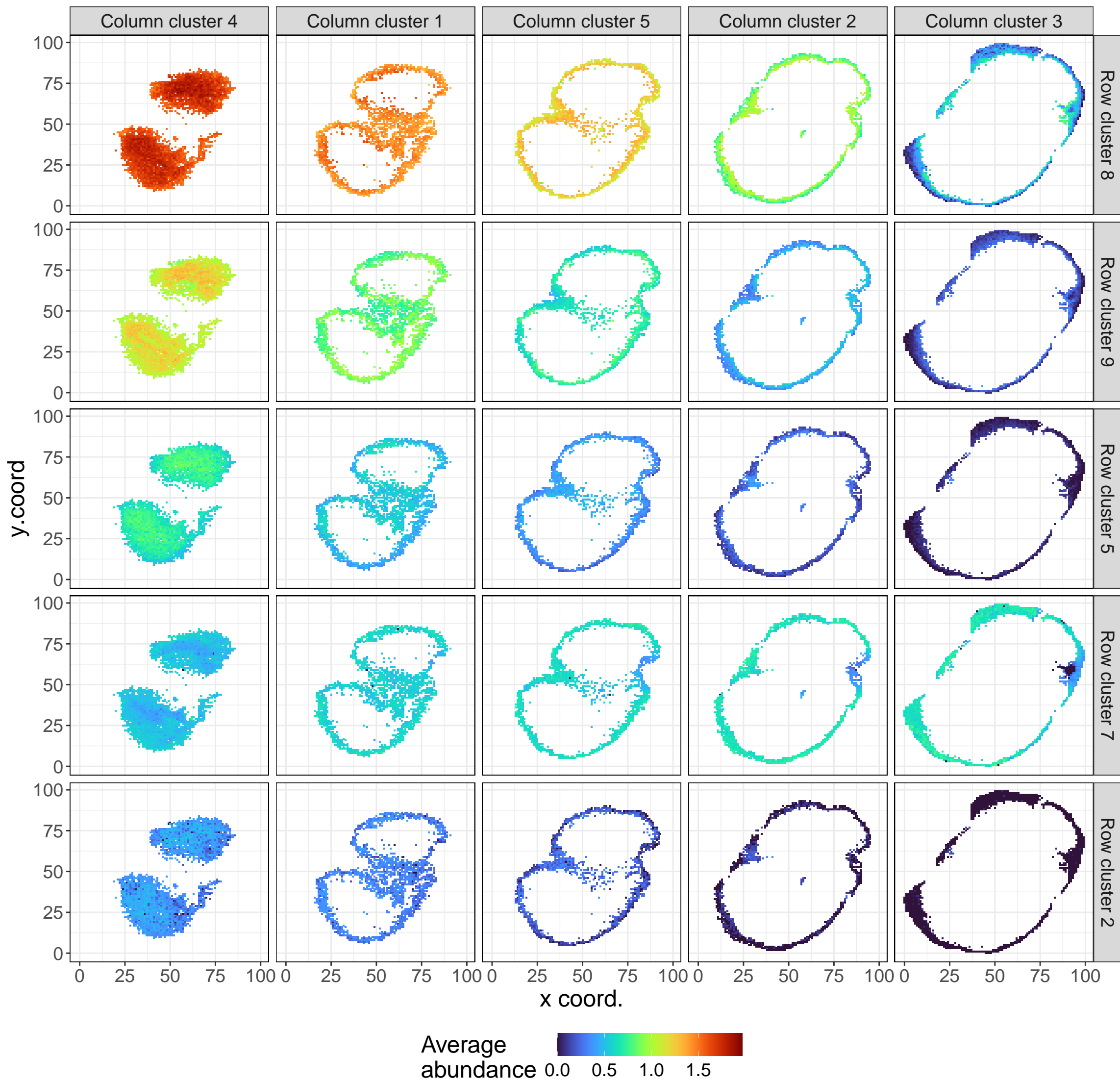

Supplement: Supplementary file 1 — Supporting Information [file BIMJ-67-e70031-s001.zip › TRIFASE_Code/REAL_DATA/GRAPHS/Figure14Suppl.pdf]

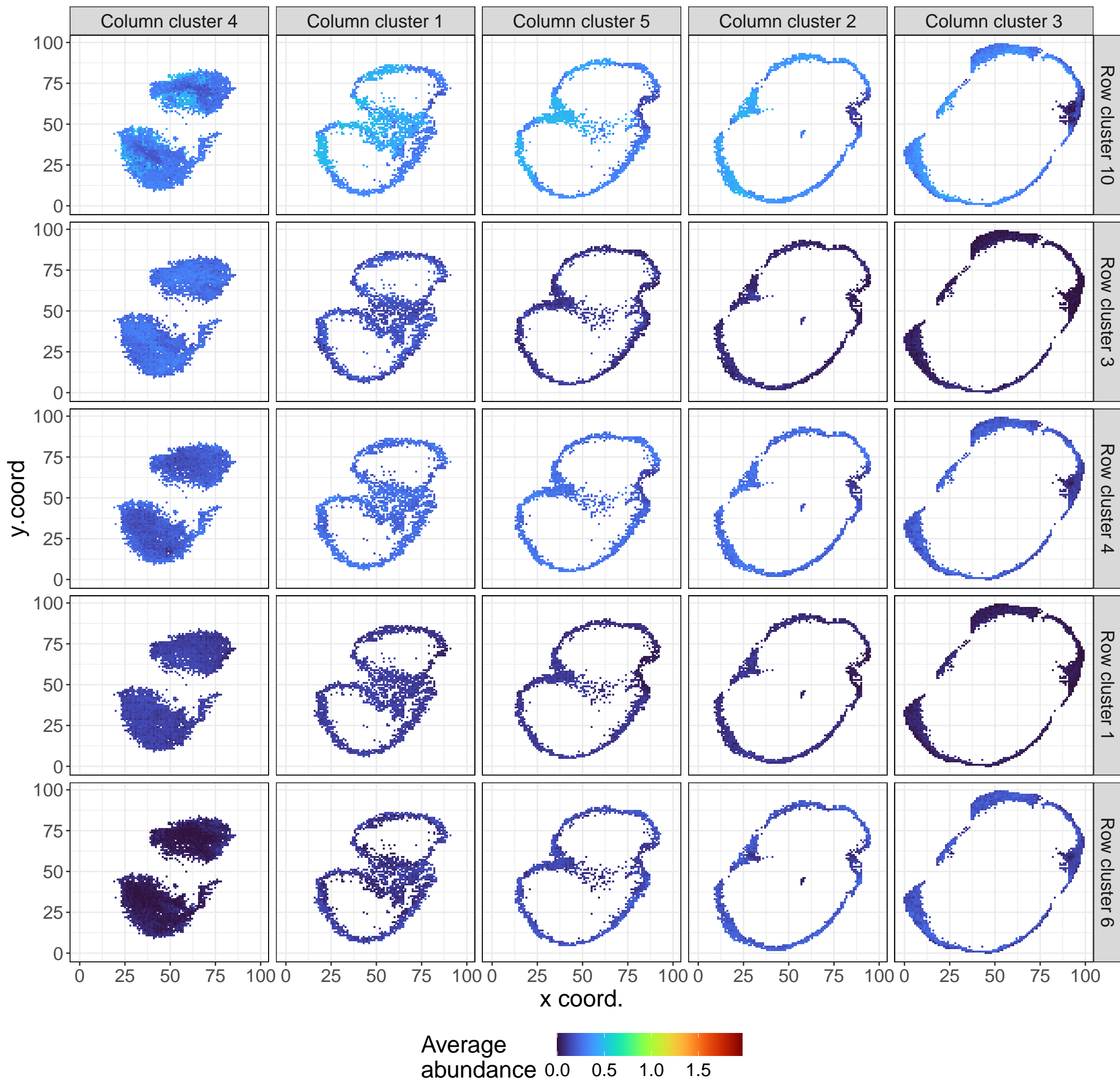

Supplement: Supplementary file 1 — Supporting Information [file BIMJ-67-e70031-s001.zip › TRIFASE_Code/REAL_DATA/GRAPHS/Figure15Suppl.pdf]

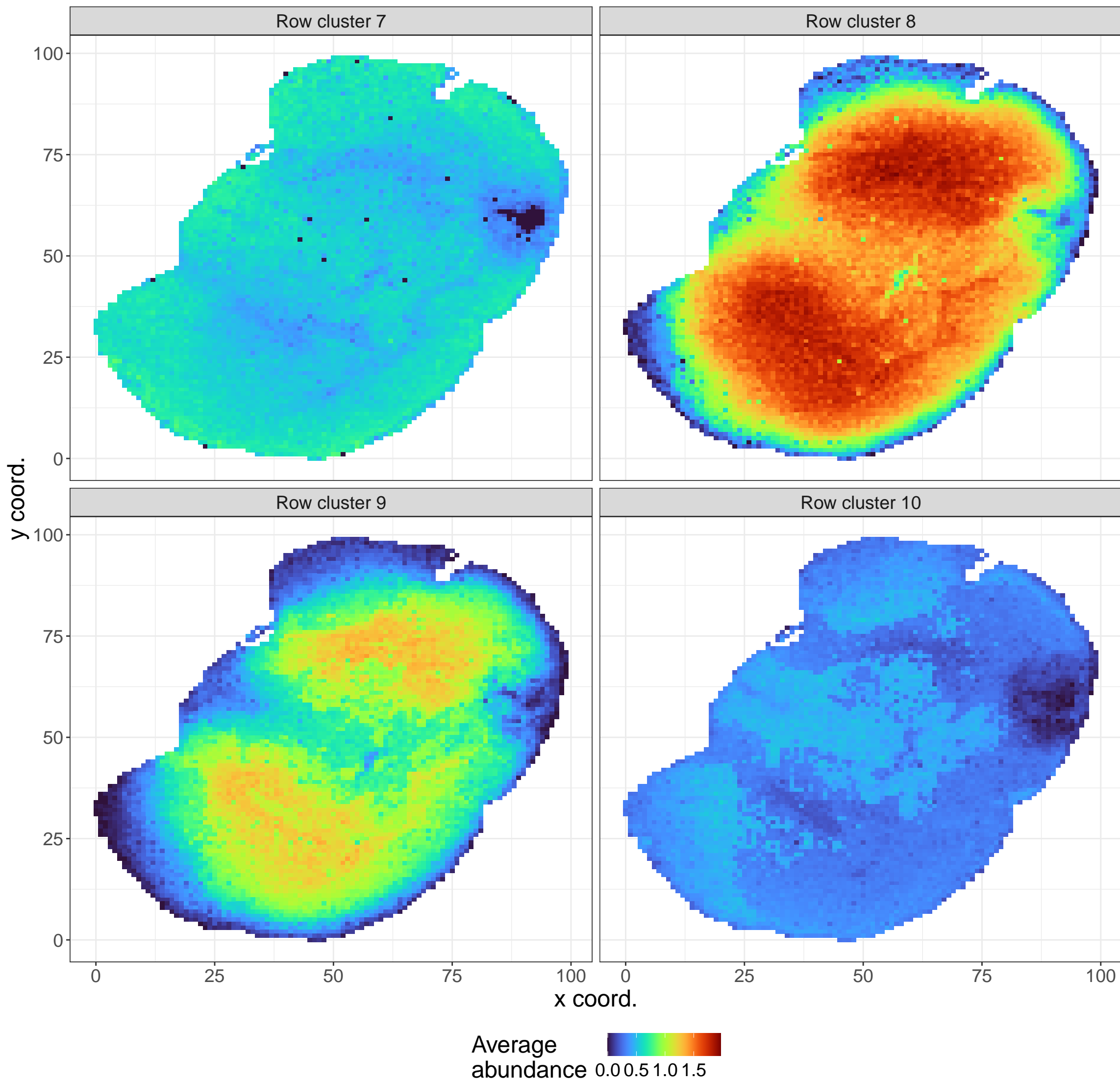

Supplement: Supplementary file 1 — Supporting Information [file BIMJ-67-e70031-s001.zip › TRIFASE_Code/REAL_DATA/GRAPHS/Figure6.pdf]

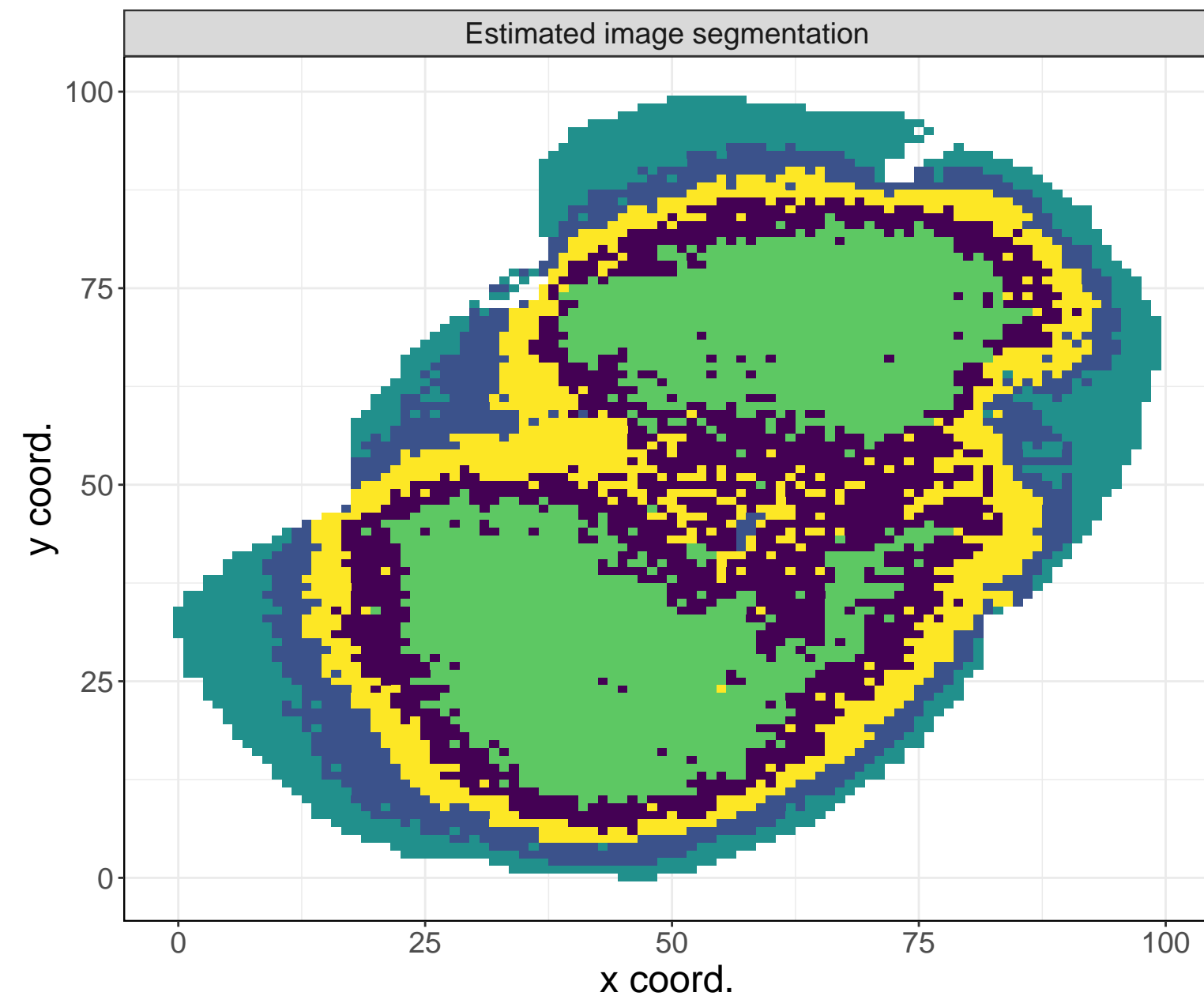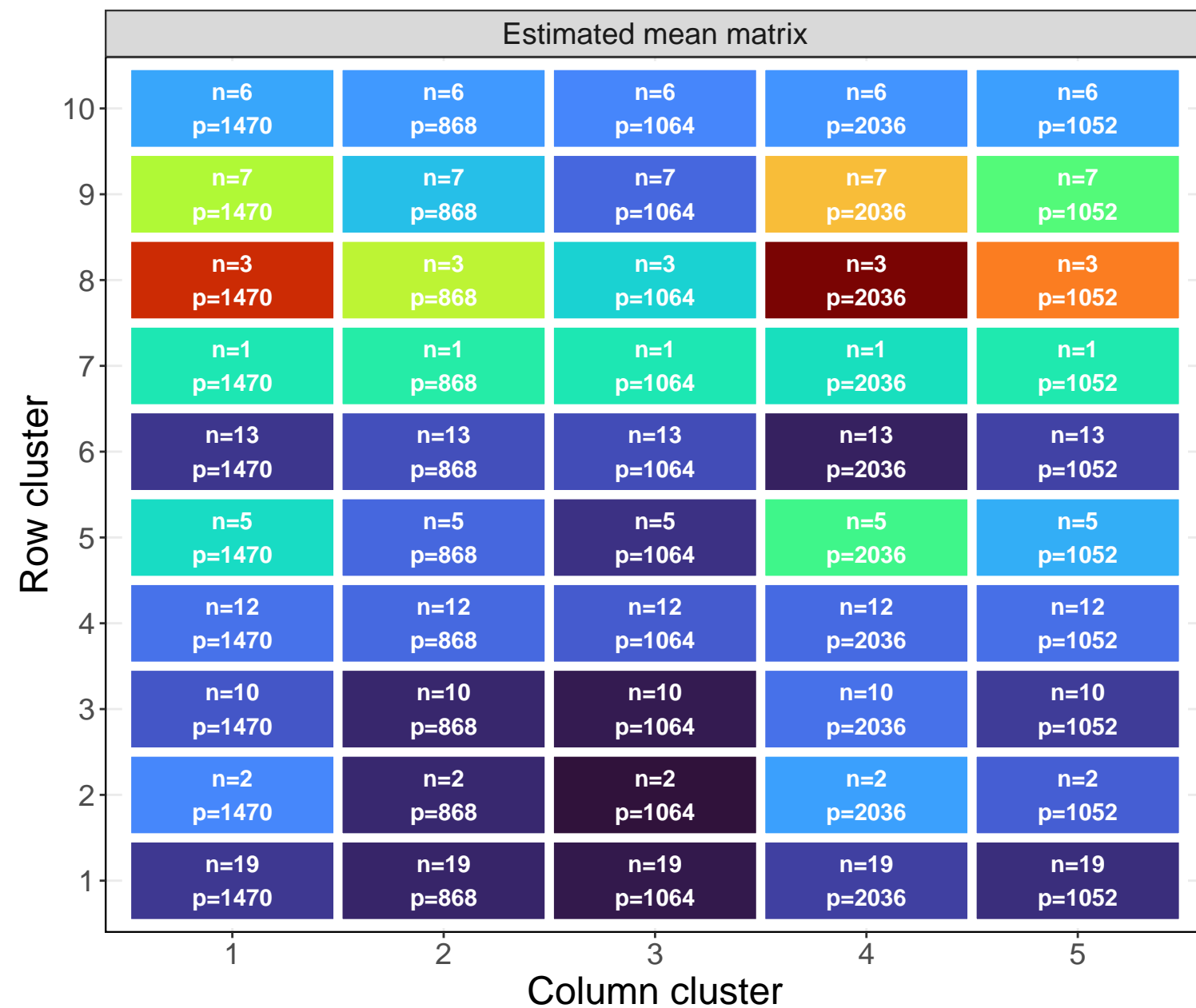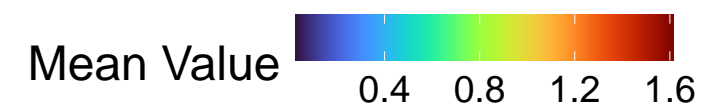

Supplement: Supplementary file 1 — Supporting Information [file BIMJ-67-e70031-s001.zip › TRIFASE_Code/REAL_DATA/GRAPHS/Figure5.pdf]
