# Supplementary material for: Spatially Informed Nonnegative Matrix Trifactorization for Coclustering Mass Spectrometry Data
Source: Biom J. 2025 Mar 19;67(2):e70031. doi: 10.1002/bimj.70031 (PMC11921289; doi:10.1002/bimj.70031)
Supplement: Supplementary file 2 — Supporting Information [file BIMJ-67-e70031-s002.pdf]

Supplementary Material to: "Spatially informed non-negative matrix tri-factorization for co-clustering mass spectrometry data"

Andrea Sottosanti, Francesco Denti, Stefania Galimberti, Davide Risso, and Giulia Capitoli

1 On the LP-FNMTF algorithm of Wang et al. (2011)

In this section, we recall the proposition underlying the LP-FNMTF algorithm introduced by Wang et al. (2011), and we emphasize a specific aspect of the dissertation that, in our view, appears to cast doubt on the validation of the preposition itself and, consequently, of the LP-FNMTF algorithm.

Proposition 1 (Wang et al., 2011)
Given a symmetric matrix $\mathbf{A}$ *and its eigen-decomposition* $\mathbf{A} = \mathbf{P}\boldsymbol{\Sigma}\mathbf{P}^T$, *where* $\boldsymbol{\Sigma} \in \mathbb{R}^{c\times c}$ *is a diagonal matrix with diagonal elements as the* c *largest eigenvalues, and* $\mathbf{P}$ *is the corresponding eigenvector matrix, the following two optimization problems are equivalent:*

$$(P1): \min_{\mathbf{C}\in\Psi} \mathrm{tr}[\mathbf{C}^T(\mathbf{I}-\mathbf{A})\mathbf{C}],$$
$$(P2): \min_{\mathbf{C}\in\Psi, \mathbf{Q}^T\mathbf{Q}=\mathbf{I}} ||\mathbf{C}-\mathbf{B}\mathbf{Q}||^2,$$

where $\mathbf{C}$ *is the clustering matrix of size* $p \times R$, $\mathbf{Q}$ *is an arbitrary orthonormal matrix and*

$$\mathbf{B} = \mathbf{P}\boldsymbol{\Sigma}^{1/2}.$$

In Proposition 1, $\mathbf{C}$ corresponds to a clustering indicator matrix, thus this result serves either for $\mathbf{F}$ and $\mathbf{G}$ in the LP-FNMTF algorithm. In addition, $\mathbf{I}-\mathbf{A}$ represents the Laplacian matrix of an undirected graph.

The proof of Proposition 1 starts with the following statement: "*(P1) is equivalent to* $\max_{\mathbf{C}\in\Psi} \mathrm{tr}[\mathbf{C}^T\mathbf{A}\mathbf{C}]$ *that is further equivalent to* $\min_{\mathbf{C}\in\Psi} ||\mathbf{C}\mathbf{C}^T - \mathbf{A}||^2$" . However, we notice that

$$\begin{aligned}\min_{\mathbf{C}\in\Psi} ||\mathbf{C}\mathbf{C}^T - \mathbf{A}||^2 &= \min_{\mathbf{C}\in\Psi} \mathrm{tr}[(\mathbf{C}\mathbf{C}^T - \mathbf{A})^T(\mathbf{C}\mathbf{C}^T - \mathbf{A})] \\ &= \min_{\mathbf{C}\in\Psi} \mathrm{tr}[\mathbf{C}\mathbf{C}^T\mathbf{C}\mathbf{C}^T] - 2\mathrm{tr}[\mathbf{A}\mathbf{C}\mathbf{C}^T] + \mathrm{tr}[\mathbf{A}^T\mathbf{A}],\end{aligned}$$

which is further equivalent to

$$\max_{\mathbf{C}\in\Psi} \mathrm{tr}[\mathbf{C}^T\mathbf{A}\mathbf{C}] - \mathrm{tr}[\mathbf{C}^T\mathbf{C}\mathbf{C}^T\mathbf{C}]/2.$$

Remembering that $\mathbf{C}$ is a cluster indicator matrix, $\mathrm{tr}[\mathbf{C}^T\mathbf{C}\mathbf{C}^T\mathbf{C}]$ is equal to the square of the cluster sizes, thus it is not constant. For this reason, $\max_{\mathbf{C}\in\Psi} \mathrm{tr}[\mathbf{C}^T\mathbf{A}\mathbf{C}]$ is not equivalent to $\min_{\mathbf{C}\in\Psi} ||\mathbf{C}\mathbf{C}^T - \mathbf{A}||^2$.

2 Derivation of the spatial non-negative matrix tri-factorization updating rules

2.1 Updating rule of $\boldsymbol{\mu}$ in Section 2.2

We consider the following minimization problem:

$$\min_{\boldsymbol{\mu}\in\mathbb{R}^{K\times R}} ||(\mathbf{X} - \mathbf{F}\boldsymbol{\mu}\mathbf{G}^T)(\mathbf{L}^{-1})^T||^2.$$

Given that the minimization is performed with respect to $\boldsymbol{\mu}$, using the cyclic property of matrix traces we rewrite the loss function as

$$||(\mathbf{X} - \mathbf{F}\boldsymbol{\mu}\mathbf{G}^T)(\mathbf{L}^{-1})^T||^2 \equiv -2\mathrm{tr}(\boldsymbol{\mu}\mathbf{G}^T\boldsymbol{\Sigma}^{-1}\mathbf{X}^T\mathbf{F}) + \mathrm{tr}(\boldsymbol{\mu}\mathbf{G}^T\boldsymbol{\Sigma}^{-1}\mathbf{G}\boldsymbol{\mu}^T\mathbf{F}^T\mathbf{F}).$$

We obtain the derivative of the two elements separately, using the results presented by Magnus and Neudecker (2019).

- Let $\tilde{\mathbf{X}} = \mathbf{G}^T\boldsymbol{\Sigma}^{-1}\mathbf{X}^T\mathbf{F}$. Then,

$$\frac{\partial}{\partial\boldsymbol{\mu}}\mathrm{tr}(\boldsymbol{\mu}\tilde{\mathbf{X}}) = \tilde{\mathbf{X}}^T = \mathbf{F}^T\mathbf{X}\boldsymbol{\Sigma}^{-1}\mathbf{G}.$$

- Let $\mathbf{A} = \mathbf{G}^T\boldsymbol{\Sigma}^{-1}\mathbf{G}$ and $\mathbf{B} = \mathbf{F}^T\mathbf{F}$. Then,

$$\frac{\partial}{\partial\boldsymbol{\mu}}\mathrm{tr}(\boldsymbol{\mu}\mathbf{A}\boldsymbol{\mu}^T\mathbf{B}) = \mathbf{B}\boldsymbol{\mu}\mathbf{A} + \mathbf{B}^T\boldsymbol{\mu}\mathbf{A}^T = 2\mathbf{F}^T\mathbf{F}\boldsymbol{\mu}\mathbf{G}^T\boldsymbol{\Sigma}^{-1}\mathbf{G}.$$

By setting

$$-2\mathbf{F}^T\mathbf{X}\boldsymbol{\Sigma}^{-1}\mathbf{G} + 2\mathbf{F}^T\mathbf{F}\boldsymbol{\mu}\mathbf{G}^T\boldsymbol{\Sigma}^{-1}\mathbf{G} = 0$$

and solving for $\boldsymbol{\mu}$, we obtain the TRIFASE updating rule of $\boldsymbol{\mu}$.

□

2.2 Updating rule of F in Section 2.2

We consider the following minimization problem:

$$\min_{\mathbf{F}\in\boldsymbol{\Psi}} ||\mathbf{X} - \mathbf{F}\boldsymbol{\mu}\mathbf{G}^T||^2. \tag{1}$$

Given that the minimization is performed with respect to $\mathbf{F}$, we rewrite the loss function as

$$||(\mathbf{X} - \mathbf{F}\boldsymbol{\mu}\mathbf{G}^T)||^2 \equiv -2\mathrm{tr}(\mathbf{X}\mathbf{G}\boldsymbol{\mu}^T\mathbf{F}^T) + \mathrm{tr}(\mathbf{F}\boldsymbol{\mu}\mathbf{G}^T\mathbf{G}\boldsymbol{\mu}^T\mathbf{F}^T).$$

We work on the two elements separately.

- Let $\mathbf{Q} = \mathbf{X}\mathbf{G}\boldsymbol{\mu}^T$. Then

$$\min_{\mathbf{F}\in\boldsymbol{\Psi}} \mathrm{tr}(\mathbf{Q}\mathbf{F}^T) = \min_{\mathbf{F}\in\boldsymbol{\Psi}} \sum_{i=1}^{n}\sum_{k=1}^{K} \mathrm{Q}_{i,k}\mathrm{F}_{i,k}. \tag{2}$$

  Since $\mathbf{F}_{i,\cdot} = (\mathrm{F}_{i,1}, \ldots, \mathrm{F}_{i,K})$ is a selection vector (i.e., all but one elements are null), then (2) is minimized by taking $\mathrm{F}_{i,k} = 1$ if $\mathrm{Q}_{i,k}$ is the minimum value across $\mathrm{Q}_{i,1}, \ldots, \mathrm{Q}_{i,K}$. Notice that

$$\mathrm{Q}_{i,k} = \mathbf{X}_{i,\cdot}\mathbf{G}\boldsymbol{\mu}_{k,\cdot}^T.$$

- Let $\mathbf{W} = \boldsymbol{\mu}\mathbf{G}^T\mathbf{G}\boldsymbol{\mu}^T$. It follows that

$$\min_{\mathbf{F}\in\boldsymbol{\Psi}} \mathrm{tr}(\mathbf{F}\mathbf{W}\mathbf{F}^T) = \min_{\mathbf{F}\in\boldsymbol{\Psi}} \sum_{i=1}^{n} \mathbf{F}_{i,\cdot}\mathbf{W}\mathbf{F}_{i,\cdot}^T.$$

  Consequently, $\mathbf{F}_{i,\cdot}\mathbf{W}\mathbf{F}_{i,\cdot}^T$ is minimized by taking $\mathrm{F}_{i,k} = 1$ if $\mathrm{W}_{k,k}$ is the minimum value across $\mathrm{W}_{1,1}, \ldots, \mathrm{W}_{K,K}$.

Then, for $i = 1, \ldots, n$, (1) is minimized by setting $\mathrm{F}_{i,k} = 1$ if

$$-2\mathbf{X}_{i,\cdot}\mathbf{G}\boldsymbol{\mu}_{k,\cdot}^T + \boldsymbol{\mu}_{k,\cdot}\mathbf{G}^T\mathbf{G}\boldsymbol{\mu}_{k,\cdot}^T \equiv ||\mathbf{X}_{i,\cdot} - \boldsymbol{\mu}_{k,\cdot}\mathbf{G}^T||^2$$

is minimum. It is straightforward to show that, replacing $\mathbf{X}$ with $\tilde{\mathbf{X}} = \mathbf{X}(\mathbf{L}^{-1})^T$ and $\mathbf{G}$ with $\tilde{\mathbf{G}} = \mathbf{L}^{-1}\mathbf{G}$ in (1), the updating rule of $\mathbf{F}$ remains unchanged.

□

2.3 Updating rule of G in Section 2.2

Let $\mathbf{G}_{j,\cdot}$ be the j-th row of $\mathbf{G}$, with $j = 1, \ldots, p$. We consider the following minimization problem:

$$\min_{\mathbf{G}_{j,\cdot}\in\{0,1\}^R} ||(\mathbf{X} - \mathbf{F}\boldsymbol{\mu}\mathbf{G}^T)(\mathbf{L}^{-1})^T||^2. \tag{3}$$

We first rewrite the loss function keeping only the elements containing $\mathbf{G}$:

$$\min_{\mathbf{G}_{j,.}\in\{0,1\}^R} ||(\mathbf{X}-\mathbf{F}\boldsymbol{\mu}\mathbf{G}^T)(\mathbf{L}^{-1})^T||^2$$
$$\equiv \min_{\mathbf{G}_{j,.}\in\{0,1\}^R} -2\text{tr}(\mathbf{G}\boldsymbol{\mu}^T\mathbf{F}^T\mathbf{X}\boldsymbol{\Sigma}^{-1}) + \text{tr}(\boldsymbol{\Sigma}^{-1}\mathbf{G}\boldsymbol{\mu}^T\mathbf{F}^T\mathbf{F}\boldsymbol{\mu}\mathbf{G}^T).$$

We now work on the two matrix traces separately. Notice that we adopt the notation $\mathbf{A}_{-j,.}$ to denote the matrix $\mathbf{A}$ without the j-th row, and $\mathbf{A}_{.,-j}$ to denote the matrix $\mathbf{A}$ without the j-th column. Therefore, $\mathbf{A}_{i,-j}$ denotes the vector corresponding to the i-th row of $\mathbf{A}$, without the j-th element.

- Let $\mathbf{Q}=\boldsymbol{\Sigma}^{-1}\mathbf{X}^T\mathbf{F}\boldsymbol{\mu}$. Then

$$\min_{\mathbf{G}_{j,.}\in\{0,1\}^R} \text{tr}(\mathbf{Q}\mathbf{G}^T) = \min_{\mathbf{G}_{j,.}\in\{0,1\}^R} \sum_{r=1}^{R} \text{Q}_{j,r}\text{G}_{j,r},$$

  which is minimized by taking $\text{G}_{j,r}=1$ if $\text{Q}_{j,r}$ is the minimum value across $\text{Q}_{j,1},\ldots,\text{Q}_{j,R}$.

- Let $\mathbf{W}=\boldsymbol{\mu}^T\mathbf{F}^T\mathbf{F}\boldsymbol{\mu}$. Then

$$\min_{\mathbf{G}_{j,.}\in\{0,1\}^R} \text{tr}(\boldsymbol{\Sigma}^{-1}\mathbf{G}\mathbf{W}\mathbf{G}^T) = \min_{\mathbf{G}_{j,.}\in\{0,1\}^R} \boldsymbol{\Sigma}^{-1}_{j,.}\mathbf{G}\mathbf{W}\mathbf{G}^T_{j,.} + \sum_{l\neq j}\boldsymbol{\Sigma}^{-1}_{l,.}\mathbf{G}\mathbf{W}\mathbf{G}^T_{l,.}.$$

  The first quantity is minimized by taking $\text{G}_{jr}=1$ when

$$\boldsymbol{\Sigma}^{-1}_{j,-j}\mathbf{G}_{-j,.}\mathbf{W}_{.,r} + \Sigma^{-1}_{j,j}\text{W}_{r,r}$$

  is minimum. Each element $\boldsymbol{\Sigma}^{-1}_{l,.}\mathbf{G}\mathbf{W}\mathbf{G}^T_{l,.}$ of the second quantity is minimized by $\text{G}_{j,r}=1$ if

$$\Sigma^{-1}_{l,j}\mathbf{W}_{r,.}\mathbf{G}^T_{l,.}$$

  is minimum. Consequently, the quantity $\text{tr}(\boldsymbol{\Sigma}^{-1}\mathbf{G}\mathbf{W}\mathbf{G}^T)$ is minimized with respect to $\mathbf{G}_{j,.}$ by taking $\text{G}_{j,r}=1$ if

$$\boldsymbol{\Sigma}^{-1}_{j,-j}\mathbf{G}_{-j,.}\mathbf{W}_{.,r} + \Sigma^{-1}_{j,j}\text{W}_{r,r} + \sum_{l\neq j}\Sigma^{-1}_{l,j}\mathbf{W}_{r,.}\mathbf{G}^T_{l,.}$$

  is minimum.

Then, for $j=1,\ldots,p$, the minimum of (3) is reached taking $\text{G}_{j,r}=1$ if

$$\ell_{jr} = -2\text{Q}_{j,r} + \boldsymbol{\Sigma}^{-1}_{j,-j}\mathbf{G}_{-j,.}\mathbf{W}_{.,r} + \Sigma^{-1}_{j,j}\text{W}_{r,r} + \sum_{l\neq j}\Sigma^{-1}_{l,j}\mathbf{W}_{r,.}\mathbf{G}^T_{l,.}$$

is minimum across $\ell_{j1},\ldots,\ell_{jR}$.

□

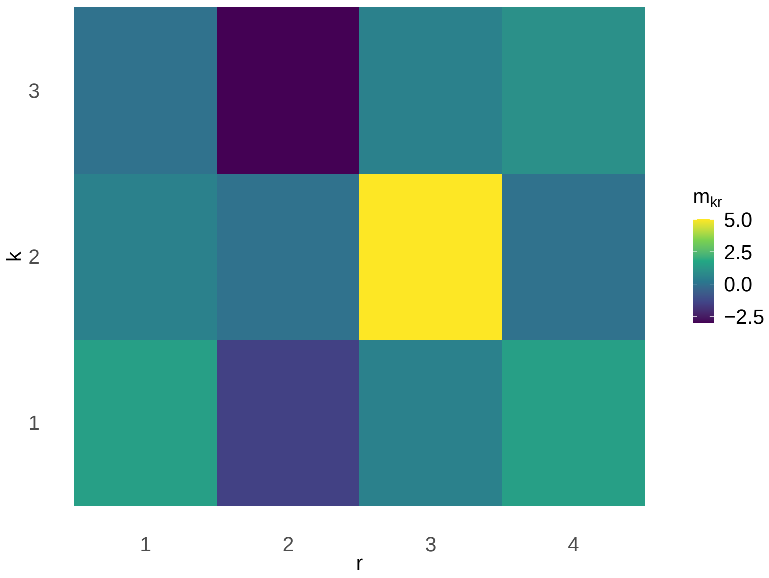

Figure 1: Mean values used to generate the centroid matrices in the simulation study.

2.4 Updating rule of τ in Section 2.3

The penalized loss function considered for estimating the parameter $\boldsymbol{\varphi} = (\tau, \phi)$ is

$$\ell_p(\tau, \phi) = \frac{1}{\tau}\mathrm{tr}[(\mathbf{X} - \mathbf{F}\boldsymbol{\mu}\mathbf{G}^T)\mathbf{K}(\mathbf{S};\phi)^{-1}(\mathbf{X} - \mathbf{F}\boldsymbol{\mu}\mathbf{G}^T)^T] + np\log\tau + n\log|\mathbf{K}(\mathbf{S};\phi)|.$$

The derivative of the penalized loss function with respect to τ is

$$\frac{\partial \ell_p(\tau,\phi)}{\partial\tau} := \ell_p^*(\tau) = -\frac{1}{\tau^2}\mathrm{tr}[(\mathbf{X} - \mathbf{F}\boldsymbol{\mu}\mathbf{G}^T)\mathbf{K}(\mathbf{S};\phi)^{-1}(\mathbf{X} - \mathbf{F}\boldsymbol{\mu}\mathbf{G}^T)^T] + \frac{np}{\tau},$$

whose unique solution is the updating rule reported in Section 2.3, which we denote here with $\hat{\tau}$. Furthermore, $\ell_p^*(\tau) > 0$ when $\tau > \hat{\tau}$, and $\ell_p^*(\tau) < 0$ when $\tau < \hat{\tau}$. Therefore, the stationary point $\hat{\tau}$ is a minimum.

□

3 Additional simulation studies and results

In this section, we report additional figures related to the simulation study proposed in Section 3 of the article. Figure 1 represents the mean values used to generate the co-cluster centroids. In particular, the kr-th block of the figure is m_{kr} that is used to draw $\mu_{k,r}^{\text{true}} \sim \mathcal{N}(m_{k,r}, 1)$.

K	R	ϕ	Row clustering strategy	Column clustering strategy
3, 6, 9	4, 8, 12	0.1, 10, 20	C, S	C, A, S

Table 1: Configurations of TRIFASE used in the simulation studies. We ran the model considering all the possible combinations of setups shown in the table, resulting in 162 possible configurations. Additionally, we also ran the methods FNMTF and k-means, considering the 9 possible combinations of K and R that appear in this table.

3.1 Case1: Spatially correlated data

We present additional outcomes obtained on the datasets generated assuming spatial dependence considering $p = 100$ and $p = 1000$ columns. We report in Table 1 all the possible combinations of the model parameters used to configure TRIFASE.

Figures 2 and 3 display the row and column clustering accuracy obtained by the versions of TRIFASE that consider $\phi = 0.1$ and $\phi = 20$, respectively. Results show that FNMTF, k-means, and the approximate versions of TRIFASE (C,A; S,A) are the ones that achieve the best results in terms of column clustering accuracy, even when the number of clusters (K, R) is misspecified. Conversely, when clustering the rows, FNMTF and k-means fail to recover the true clusters. TRIFASE (C,A; S,A) achieves the best results when the number of row clusters is set equal to the ground truth ($K = K^{\text{true}} = 3$) and ϕ is sufficiently large (see Figure 3).

We also compare the methods in terms of computational time (expressed in seconds). We display in Figure 4 the results obtained using TRIFASE with $\phi = 0.1$ and $\phi = 20$. We see that the fastest versions of TRIFASE are (C,A) and (S,A), while the remaining four versions (C,C; C,S; S,C; S,S) tend to be more expensive in terms of computational time.

The versions (C, A) and (S, A) of TRIFASE are practically equivalent in terms of estimation time and are the most efficient among the multiple implementations proposed in this article. Their estimation time is also comparable to FNMTF and k-means. For this reason, we further investigated the scalability of the version (C, A) under different experimental conditions, varying the number of rows and columns of the data matrix. Therefore, we generated 30 datasets considering $n = 150, 750, 1500$ and $p = 200, 1000, 2000$, while $K^{\text{true}} = 3$ and $R^{\text{true}} = 4$. Results are displayed in Figure 5.

We analyze the distribution of the number of non-empty clusters under different setups of TRIFASE and the competing models. In Figure 6, we display how the number of non-empty row clusters varies based on 30 simulation experiments with $p = 100$ columns (left panel) and $p = 1000$ columns (right panel). All versions of TRIFASE consider $\phi = 10$. The results show no differences in the number of row clusters detected by the models. Additionally, we present the same type of analysis on the row clusters and column clusters in Figures 7 and 8, considering $\phi = 0.1$ and $\phi = 20$. The results confirm that the TRIFASE versions (C,A) and (S,A) perform better than the other versions in terms of selecting the true number of clusters. Moreover, all versions of TRIFASE with $\phi = 0.1$

perform similarly to FNMTF because the spatial range considered is very narrow. It is worth noting that these results must also be interpreted in conjunction with clustering accuracy. Particularly for the rows, FNMTF is capable of detecting the correct number of clusters but fails to recover the correct partition of the data.

In Figure 9, we report the distributions of the estimated values of the kernel parameter τ, varying K, R and ϕ. The versions of TRIFASE that well recover the correct value of τ^{true} are (C,A) and (S,A) when ϕ is set equal to ϕ^{true}.

3.2 Case 2: independent data

We also investigate the performance of TRIFASE, FNMTF, and k-means when the columns of the dataset are not spatially correlated. The setups of TRIFASE considered are still 162, but in this framework, we considered the values 0.001, 0.1, and 10 for ϕ.

We report here the row and column clustering accuracy obtained on the 30 simulated datasets with $p = 100$ columns and $p = 1000$ columns. Figures 10, 11, and 12 show the results with $\phi = 0.001$, $\phi = 0.1$, and $\phi = 10$, respectively. When the kernel scale ϕ is sufficiently small, the kernel matrix $\mathbf{K}$ becomes similar to the diagonal matrix. It follows that TRIFASE works very similarly to FNMTF, and both methods generally perform better than k-means. When the spatial scale of the model is increased, the versions of TRIFASE that work better are (C,A) and (S,A), both when the number of clusters is correctly specified or misspecified. In clustering the rows, FNMTF and k-means can recover the true clusters both when the number of clusters is correctly specified or misspecified, while they cannot retrieve the column clusters when R is misspecified.

3.3 Convergence diagnostic

We report in Figure 13 the analysis of convergence of the six versions of TRIFASE proposed in this work. We run each version 50 times on the same dataset, initializing the estimation from different starting points. Overall, the figure shows that most runs of the (C, A) and (S, A) versions converge to the same local solution, with few exceptions. In contrast, the other four versions mostly converge to sub-optimal points.

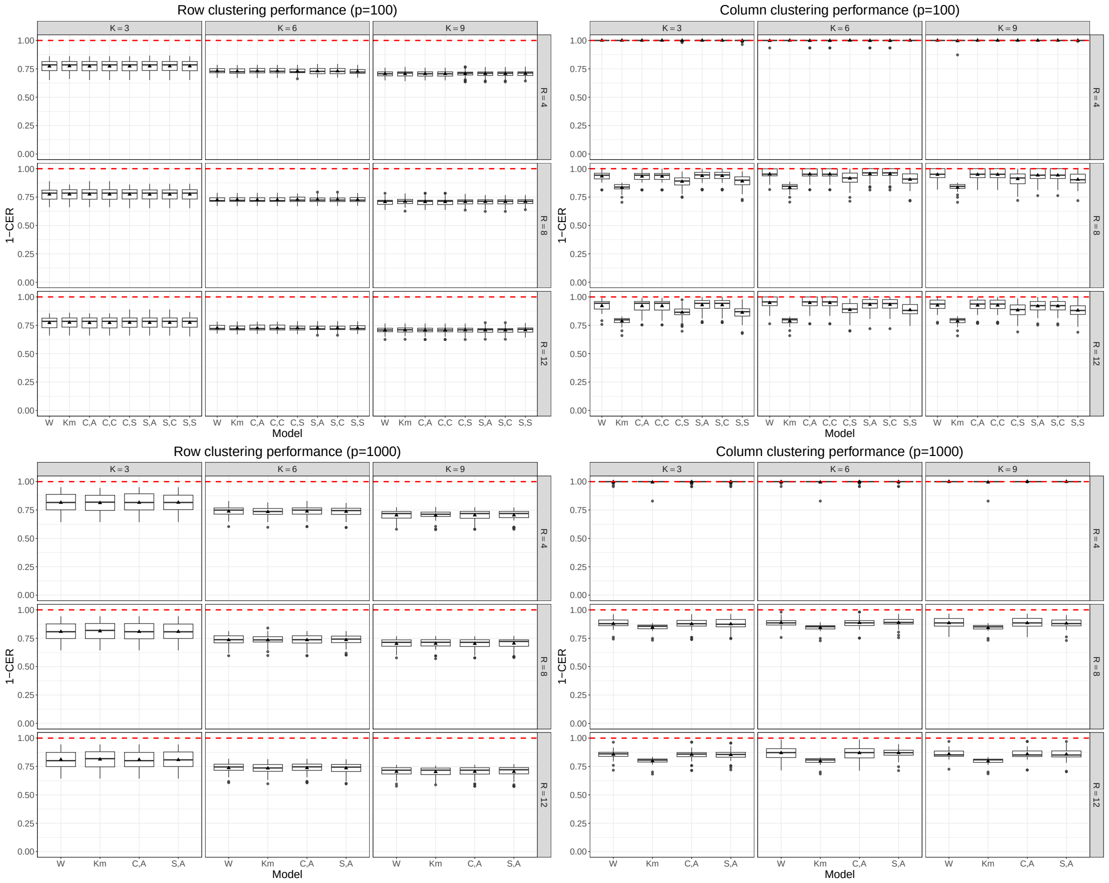

Figure 2: Results obtained on 30 simulated datasets of dimension 90 × 100 (top row) and on 30 simulated datasets of dimension 90 × 1000 (bottom row). All the datasets were generated assuming $K^{\text{true}} = 3$ and $R^{\text{true}} = 4$ and with spatial correlation among columns ($\tau^{\text{true}} = 3$, $\phi^{\text{true}} = 10$). We employed six different estimation strategies of TRIFASE (C,A; C,C; C,S; S,A; S,C; S,S), k-means (Km), and FNMTF (W). The graphs display the concordance of the estimated row clustering (left column) and column clustering (right column) with the reference labels, assuming different values of K and R. For the analysis of the 30 datasets of dimension 90 × 1000, we restricted our attention only to the versions of TRIFASE that make use of Step 3A (C,A; S,A) to reduce the computation burden. Every version of TRIFASE has been run setting $\phi = 0.1$.

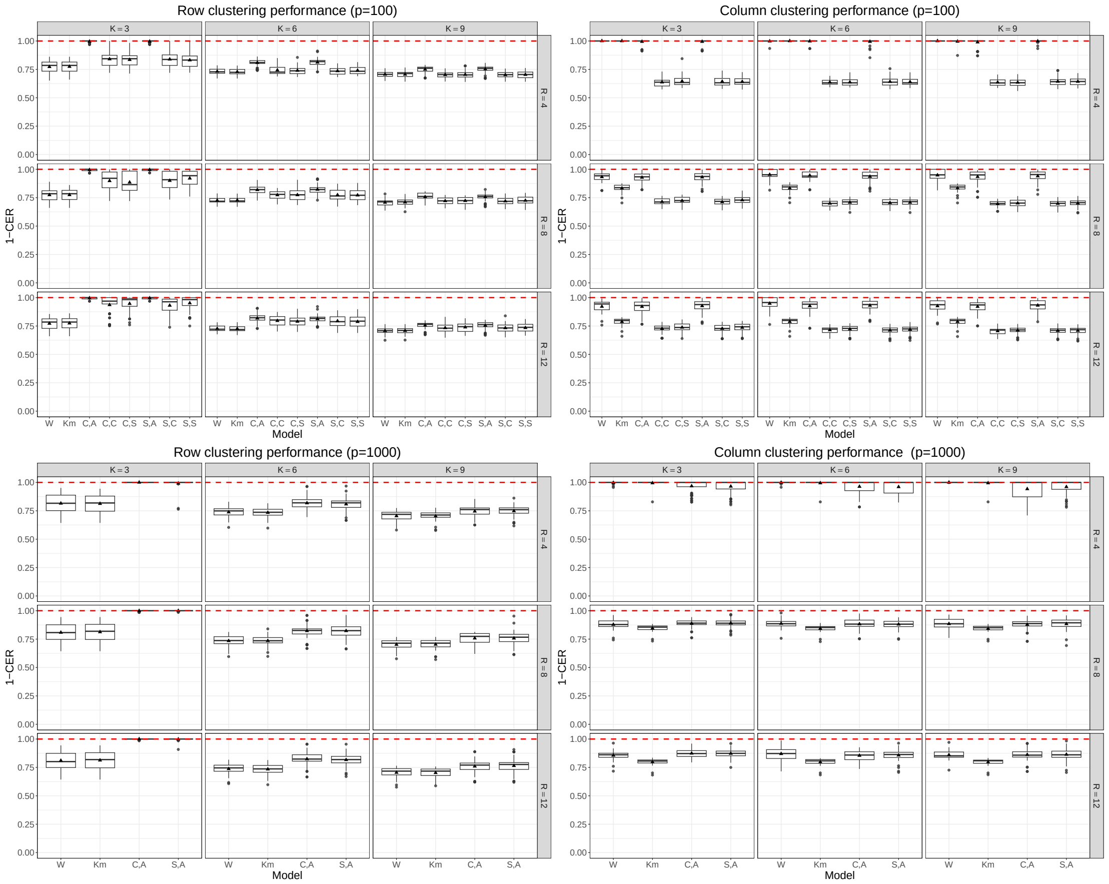

Figure 3: Results obtained on 30 simulated datasets of dimension 90 × 100 (top row) and on 30 simulated datasets of dimension 90 × 1000 (bottom row). All the datasets were generated assuming $K^{\text{true}} = 3$ and $R^{\text{true}} = 4$ and with spatial correlation among columns ($\tau^{\text{true}} = 3$, $\phi^{\text{true}} = 10$). We employed six different estimation strategies of TRIFASE (C,A; C,C; C,S; S,A; S,C; S,S), k-means (Km), and FNMTF (W). The graphs display the concordance of the estimated row clustering (left column) and column clustering (right column) with the reference labels, assuming different values of K and R. For the analysis of the 30 datasets of dimension 90 × 1000, we restricted our attention only to the versions of TRIFASE that make use of Step 3A (C,A; S,A) to reduce the computation burden. Every version of TRIFASE has been run setting $\phi = 20$.

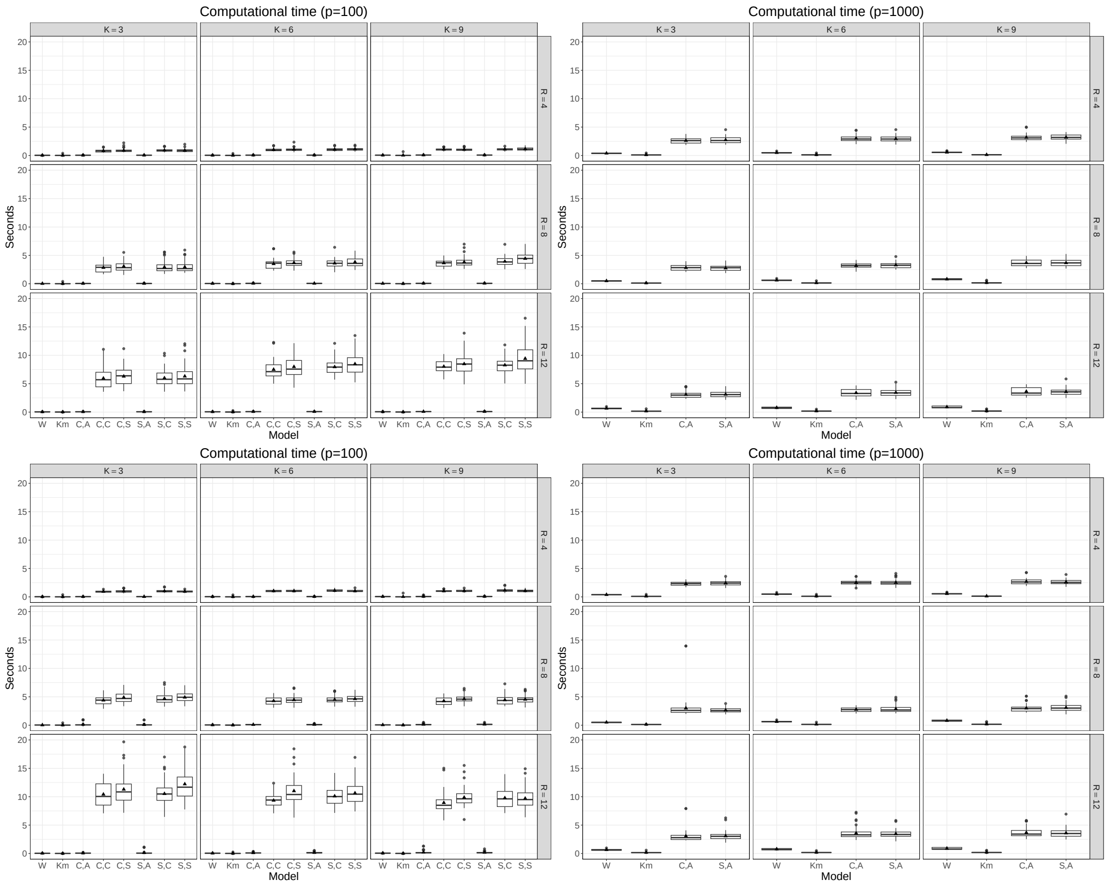

Figure 4: Analysis of the computational cost (in seconds) on 30 simulated datasets of dimension 90×100 (left column) and on 30 simulated datasets of dimension 90×1000 (right column), varying the number of row and column clusters. In the top row, we display the results obtained running TRIFASE with $\phi = 0.1$, and on the bottom row running TRIFASE with $\phi = 20$.

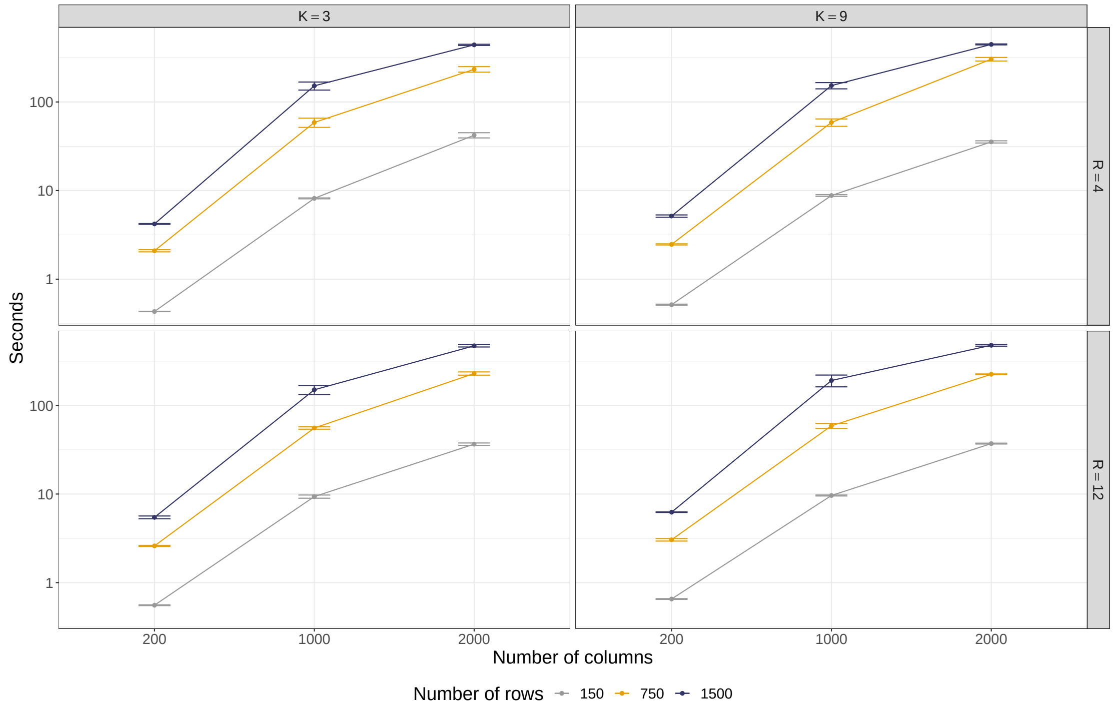

Figure 5: Distribution of the computational cost (in seconds) of the version (C,A) of TRIFASE under different experimental conditions. For every combination of the number of rows and columns considered, we generated 30 simulated datasets using $K^{\text{true}} = 3$ and $R^{\text{true}} = 4$. Then, we estimated our model using $K \in \{K^{\text{true}}, 3K^{\text{true}}\}$ and $R \in \{R^{\text{true}}, 3R^{\text{true}}\}$. Confidence bars denote the minimum and the maximum estimation time recorded for each combination of parameters.

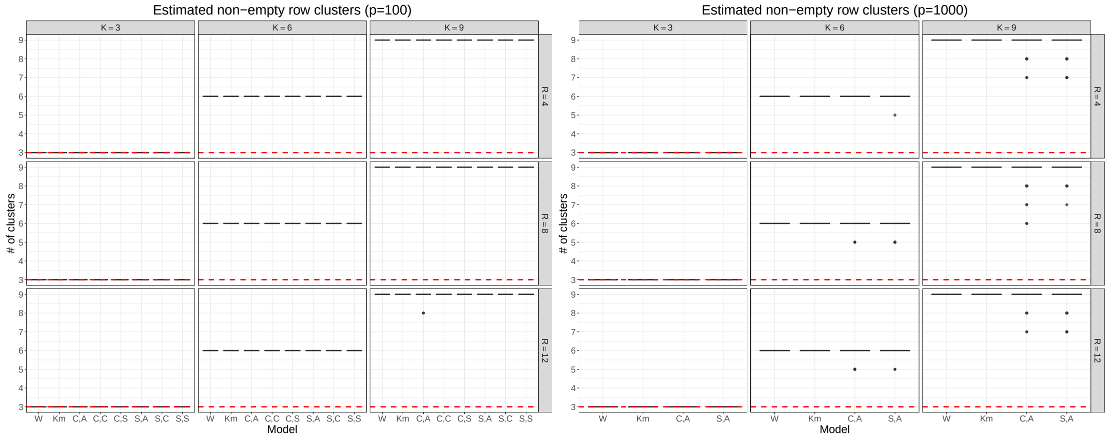

Figure 6: Distribution of the non-empty row clusters obtained on 30 simulated datasets of dimension 90×100 (left panel) and on 30 simulated datasets of dimension 90×1000 (right), varying the number of row clusters K and column clusters R. Red lines denote R^{true}. Every version of TRIFASE has been run setting $\phi = 10$.

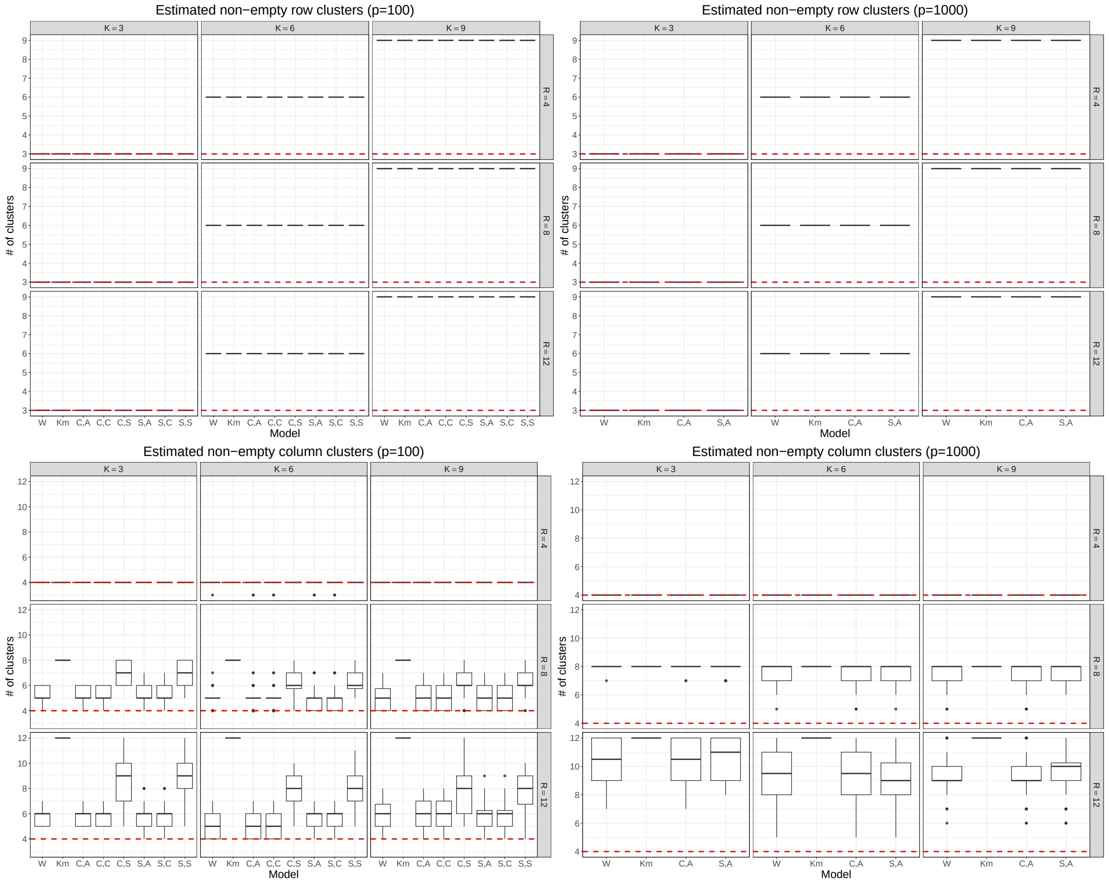

Figure 7: Distribution of the non-empty row clusters (top line) and column clusters (bottom line) obtained on 30 simulated datasets of dimension 90×100 (left column) and on 30 simulated datasets of dimension 90×1000 (right column), varying the number of row clusters K and column clusters R. Red lines denote R^{true}. Every version of TRIFASE has been run setting $\phi = 0.1$.

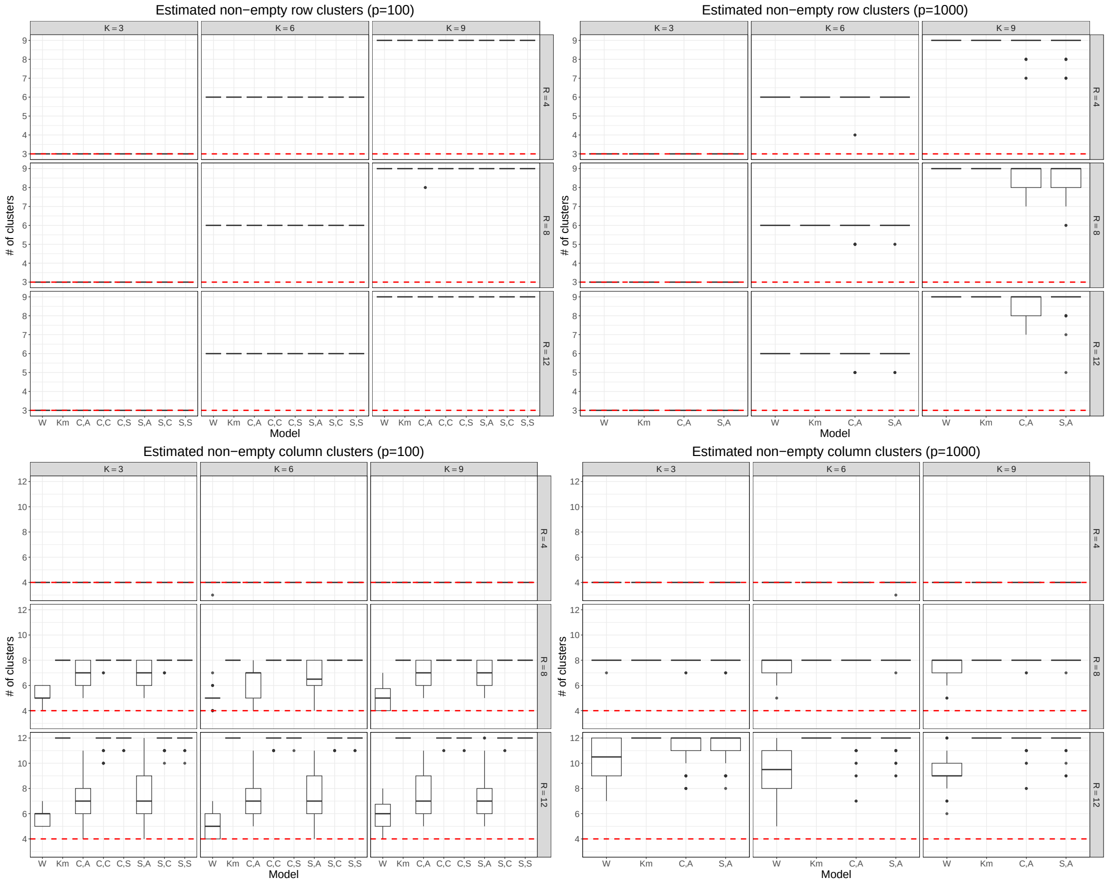

Figure 8: Distribution of the non-empty row clusters (top line) and column clusters (bottom line) obtained on 30 simulated datasets of dimension 90×100 (left column) and on 30 simulated datasets of dimension 90×1000 (right column), varying the number of row clusters K and column clusters R. Red lines denote R^{true}. Every version of TRIFASE has been run setting $\phi = 20$.

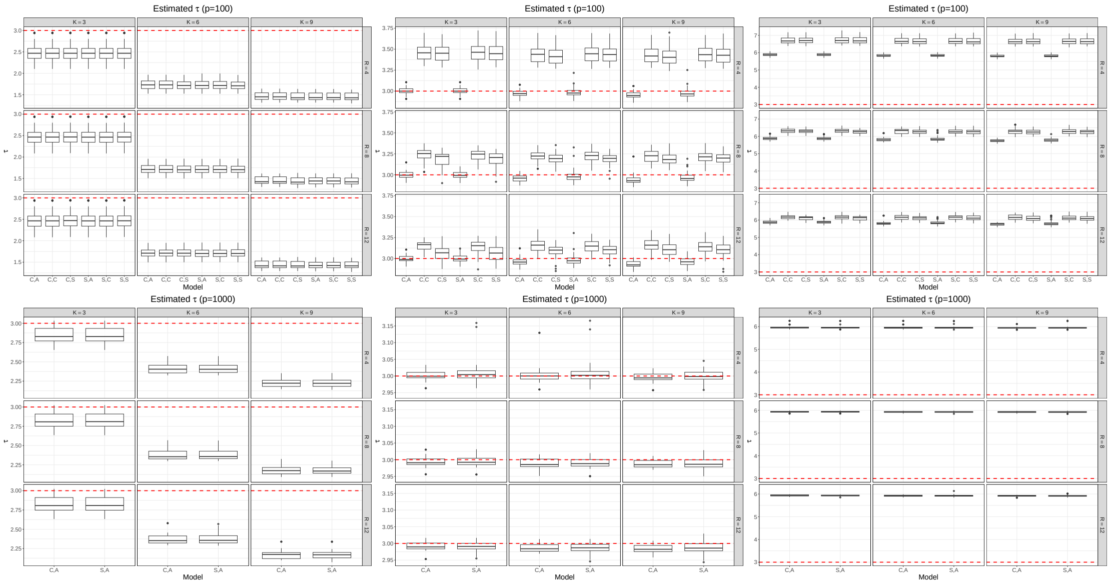

Figure 9: Distributions of the estimates of τ obtained on the 30 datasets of dimension 90×100 (top row) and on the 30 datasets of dimension 90×1000 (bottom row), varying the number of row clusters K and column clusters R, and setting $\phi = 0.1$ (left column), $\phi = 10$ (central column) and $\phi = 20$ (right column). The red lines represent τ^{true}.

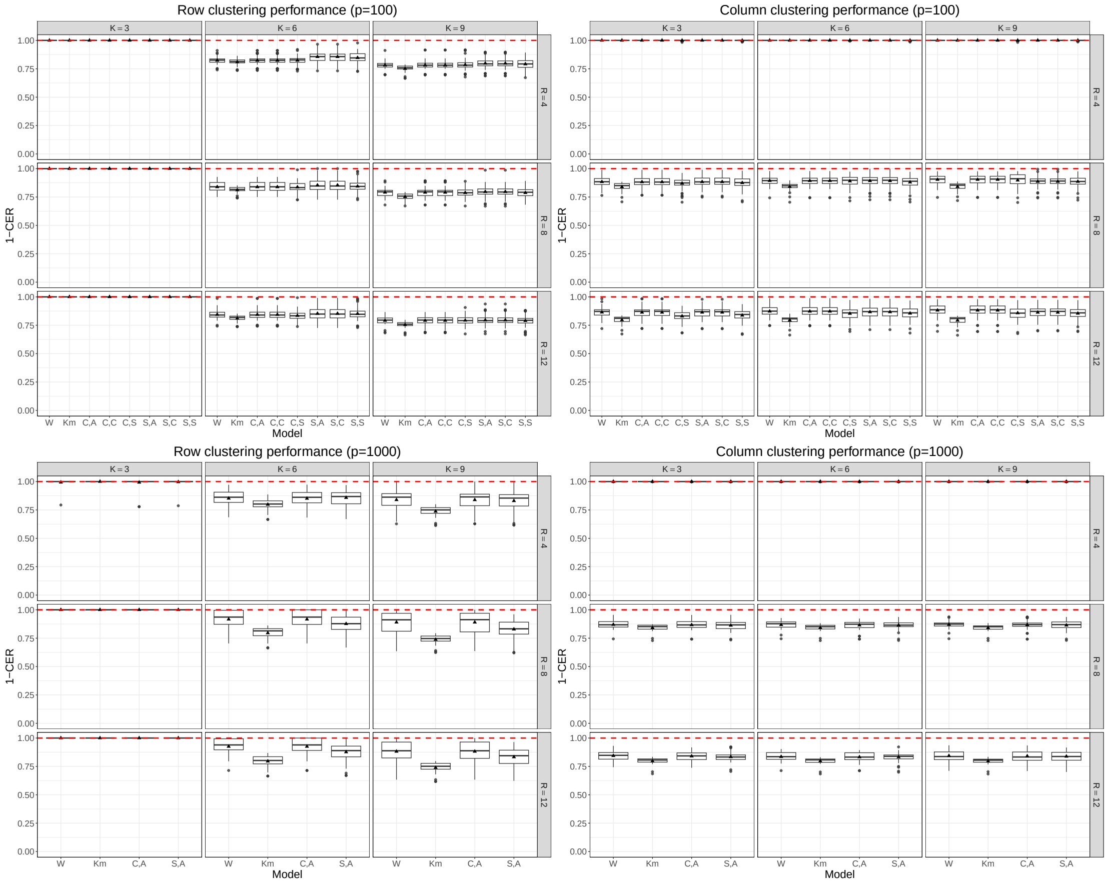

Figure 10: Results obtained on 30 simulated datasets of dimension 90×100 (top row) and on 30 simulated datasets of dimension 90×1000 (bottom row), generated without spatial correlation among columns. All the datasets were generated assuming $K^{\text{true}} = 3$ and $R^{\text{true}} = 4$ and with spatial correlation among columns ($\tau^{\text{true}} = 3$, $\phi^{\text{true}} = 10$). We employed six different estimation strategies of TRIFASE (C,A; C,C; C,S; S,A; S,C; S,S), k-means (Km), and FNMTF (W). The graphs display the concordance of the estimated row clustering (left column) and column clustering (right column) with the reference labels, assuming different values of K and R. For the analysis of the 30 datasets of dimension 90×1000, we restricted our attention only to the versions of TRIFASE that make use of Step 3A (C,A; S,A) to reduce the computation burden. Every version of TRIFASE has been run setting $\phi = 0.001$.

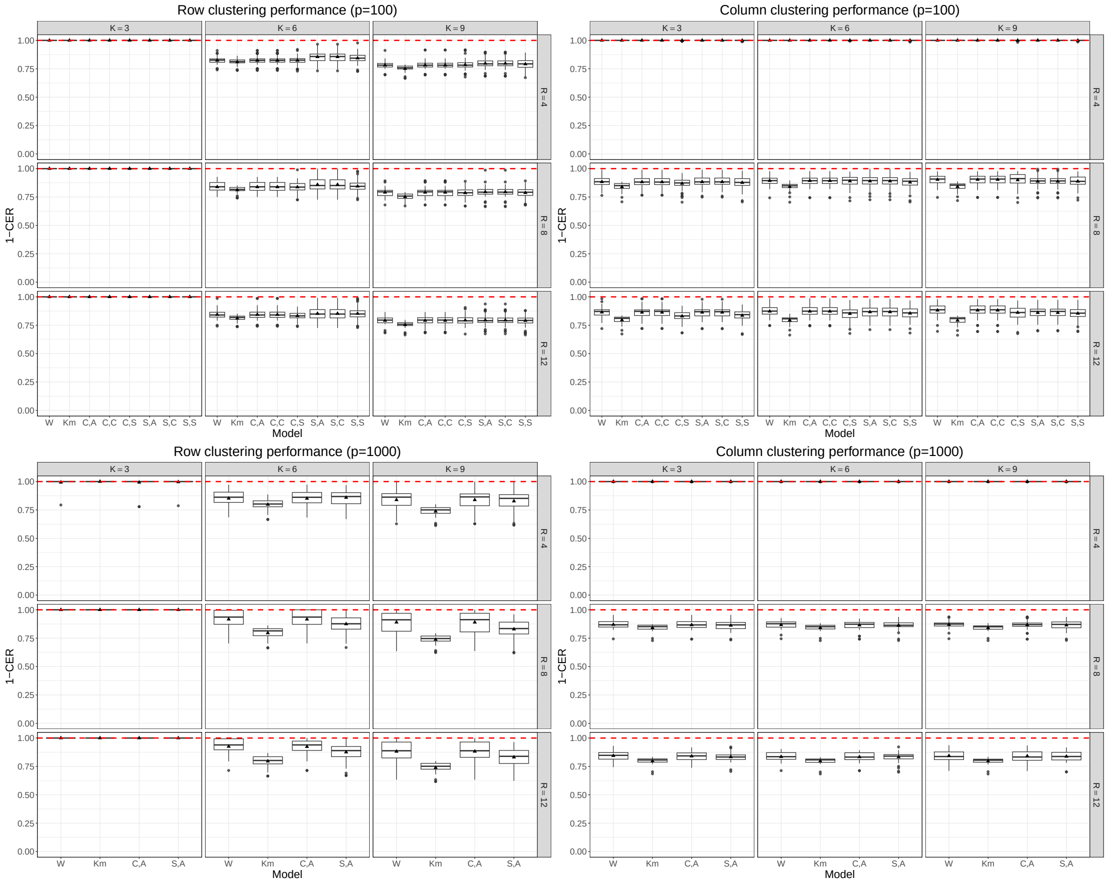

Figure 11: Results obtained on 30 simulated datasets of dimension 90 × 100 (top row) and on 30 simulated datasets of dimension 90 × 1000 (bottom row), generated without spatial correlation among columns. All the datasets were generated assuming $K^{\text{true}} = 3$ and $R^{\text{true}} = 4$ and with spatial correlation among columns ($\tau^{\text{true}} = 3$, $\phi^{\text{true}} = 10$). We employed six different estimation strategies of TRIFASE (C,A; C,C; C,S; S,A; S,C; S,S), k-means (Km), and FNMTF (W). The graphs display the concordance of the estimated row clustering (left column) and column clustering (right column) with the reference labels, assuming different values of K and R. For the analysis of the 30 datasets of dimension 90 × 1000, we restricted our attention only to the versions of TRIFASE that make use of Step 3A (C,A; S,A) to reduce the computation burden. Every version of TRIFASE has been run setting $\phi = 0.1$.

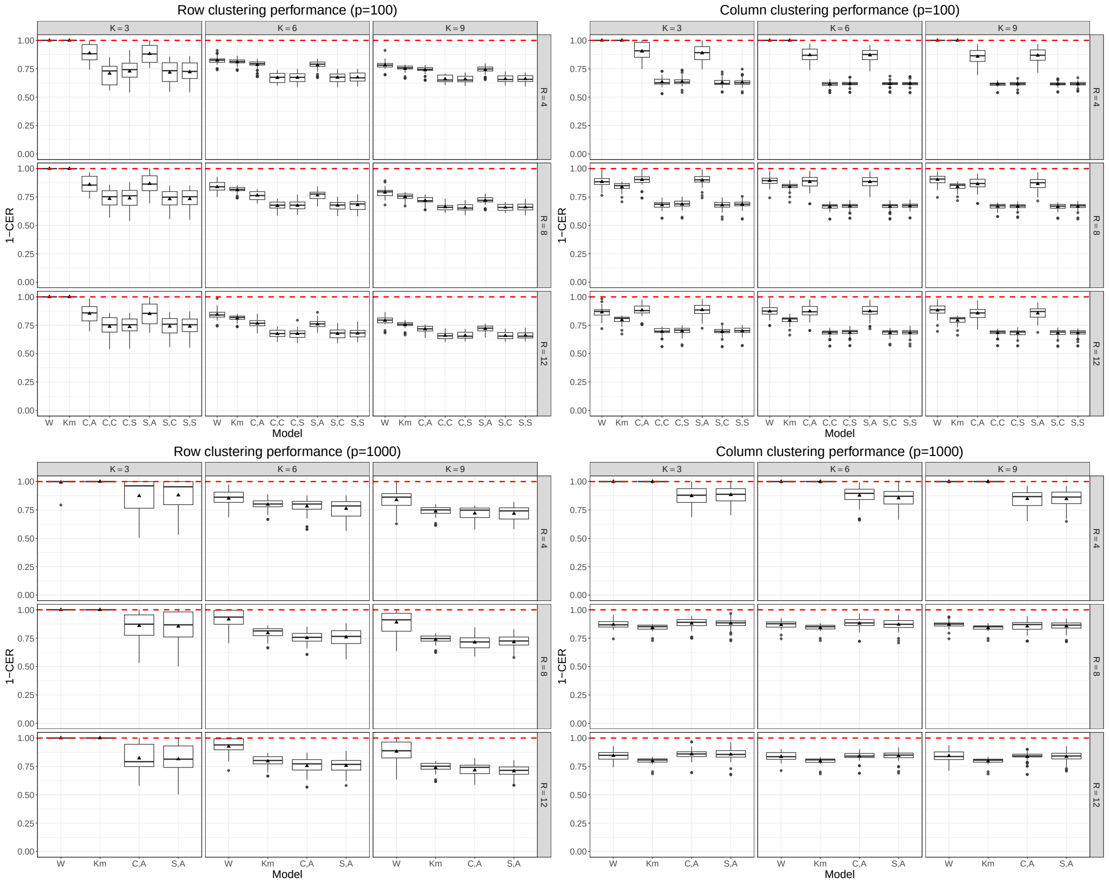

Figure 12: Results obtained on 30 simulated datasets of dimension 90×100 (top row) and on 30 simulated datasets of dimension 90×1000 (bottom row), generated without spatial correlation among columns. All the datasets were generated assuming $K^{\text{true}} = 3$ and $R^{\text{true}} = 4$ and with spatial correlation among columns ($\tau^{\text{true}} = 3$, $\phi^{\text{true}} = 10$). We employed six different estimation strategies of TRIFASE (C,A; C,C; C,S; S,A; S,C; S,S), k-means (Km), and FNMTF (W). The graphs display the concordance of the estimated row clustering (left column) and column clustering (right column) with the reference labels, assuming different values of K and R. For the analysis of the 30 datasets of dimension 90×1000, we restricted our attention only to the versions of TRIFASE that make use of Step 3A (C,A; S,A) to reduce the computation burden. Every version of TRIFASE has been run setting $\phi = 10$.

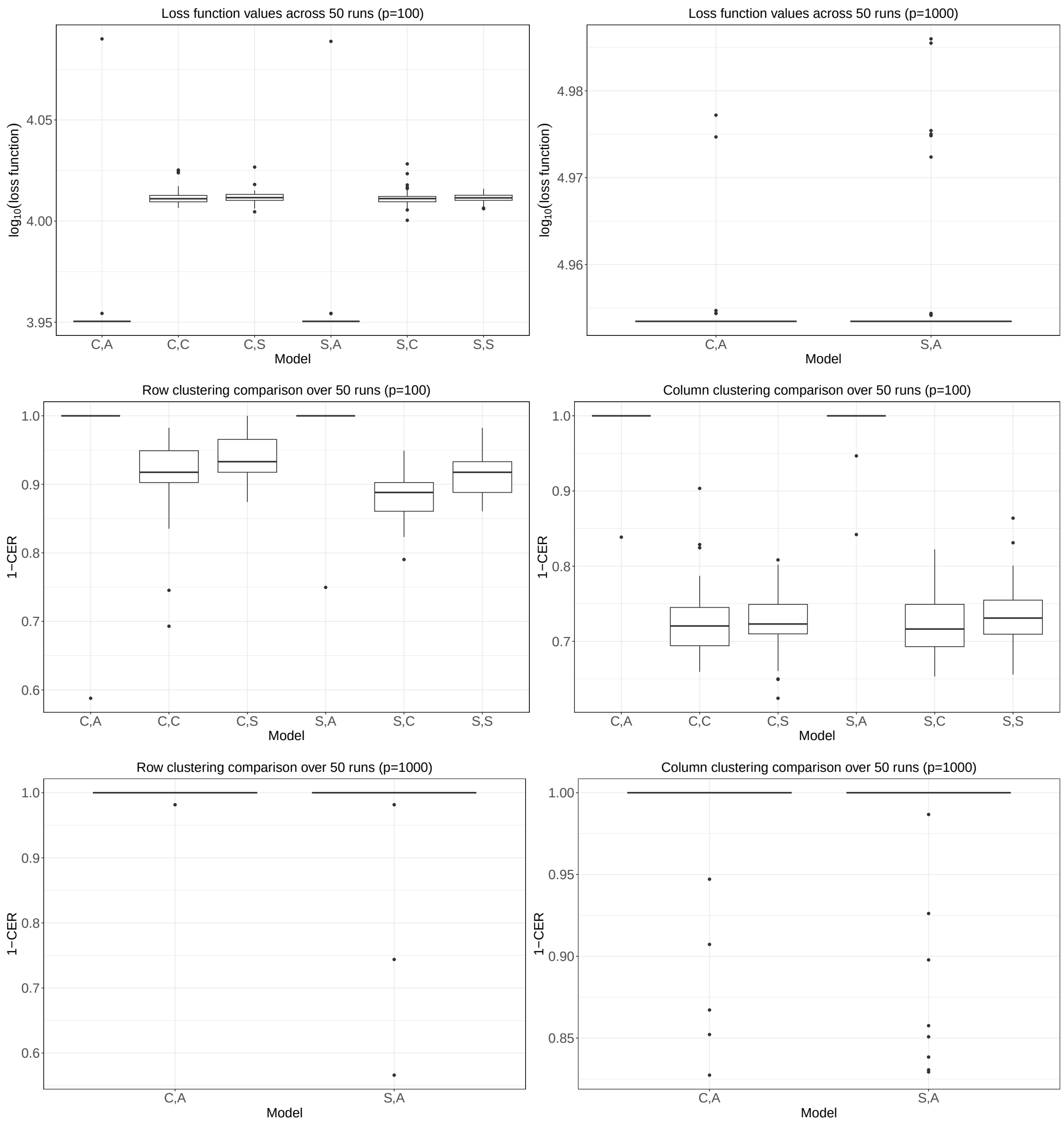

Figure 13: Top row: loss function value (in $\log_{10}$ scale) obtained across 50 runs of a simulated dataset of dimension 90×100 (left column) and on a dataset of dimension 90×1000 (right column). The dataset was generated assuming $K^{\text{true}} = 3$ and $R^{\text{true}} = 4$ and with spatial correlation among columns ($\tau^{\text{true}} = 3$, $\phi^{\text{true}} = 10$). We employed six different estimation strategies of TRIFASE (C,A; C,C; C,S; S,A; S,C; S,S) and we restricted our attention only to the versions of TRIFASE that make use of Step 3A (C,A; S,A) to reduce the computation burden. Every version of TRIFASE has been run setting $K = 3$, $R = 4$, and $\phi = 10$. Central and bottom rows: distribution of (1-CER) values computed between the partition corresponding to the minimum loss function obtained across the 50 runs and all the remaining 49 runs, using the dataset with $p = 100$ columns (central row) and with $p = 1000$ (bottom row).

3.4 Real data application

Figures 14-15 report the average expressions of the ten lipid clusters across the five different spatial clusters pinpointed by TRIFASE. Therefore, the (k, r)-th panel displays the pixels assigned to the r-th cluster, coloured according to the average abundance of lipids assigned to the k-th cluster. For easier understanding, the five-column clusters have been rearranged from left to right, ordering the brain regions from the most central to the most peripheral. The row clusters, instead, have been rearranged from top to bottom based on the abundance of lipids in the central region of the brain, denoted as Column cluster 4. Therefore, clusters at the top indicate a high abundance in Column Cluster 4, while clusters at the bottom indicate a low abundance. These graphs are helpful to visualize different activation patterns among different areas of the brain detected by the spatial clustering.

References

Jan R. Magnus and Heinz Neudecker. *Matrix Differential Calculus with Applications in Statistics and Econometrics.* Wiley series in probability and statistics. Wiley, Hoboken (N.J.), 3rd ed edition, 2019. ISBN 978-1-119-54119-6 978-1-119-54121-9 978-1-119-54116-5.

Hua Wang, Feiping Nie, Heng Huang, and Fillia Makedon. Fast Nonnegative Matrix Tri-Factorization for Large-Scale Data Co-Clustering. In *Proceedings of the Twenty-Second international joint conference on Artificial Intelligence - Volume Volume Two*, pages 1553–1558, Barcelona, Catalonia, Spain, 2011. AAAI Press. ISBN 978-1-57735-514-4.

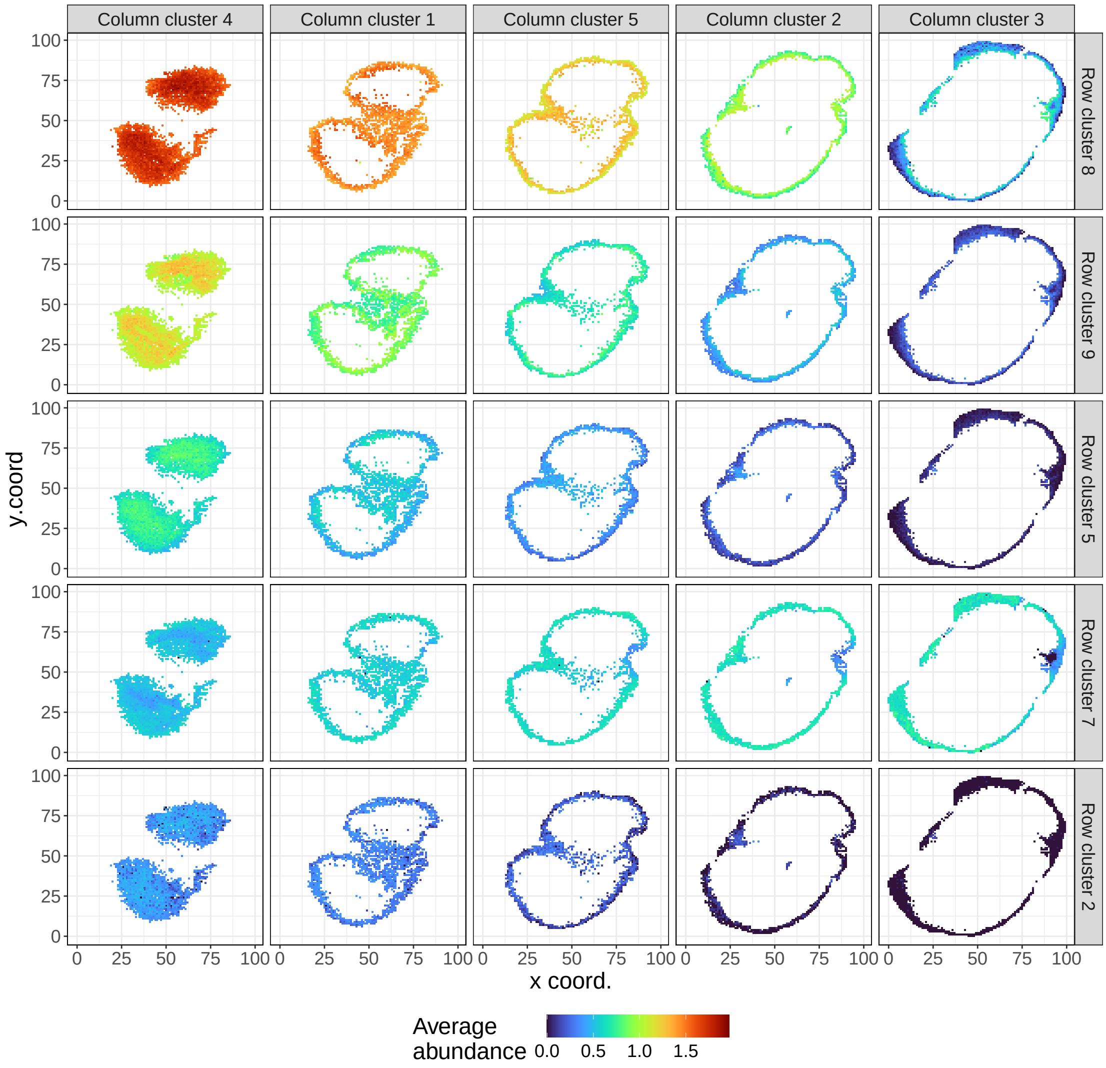

Figure 14: Co-clustering structure estimated by TRIFASE on the MALDI-MSI data. The columns represent the pixels assigned to the five column clusters, rearranged from left to right following the brain regions from the most central to the most peripheral. Pixels are coloured according to the average abundance of the signals assigned to each row cluster. Rows denote the clusters of lipids, which have been rearranged from top to bottom in decreasing order based on the abundance in the central cluster of pixels, denoted as Column cluster 4. Therefore, Row cluster 8 contains the lipids with the largest abundance in Column cluster 4. This figure displays only the first group of five row clusters, while the second group is shown in Figure 15.

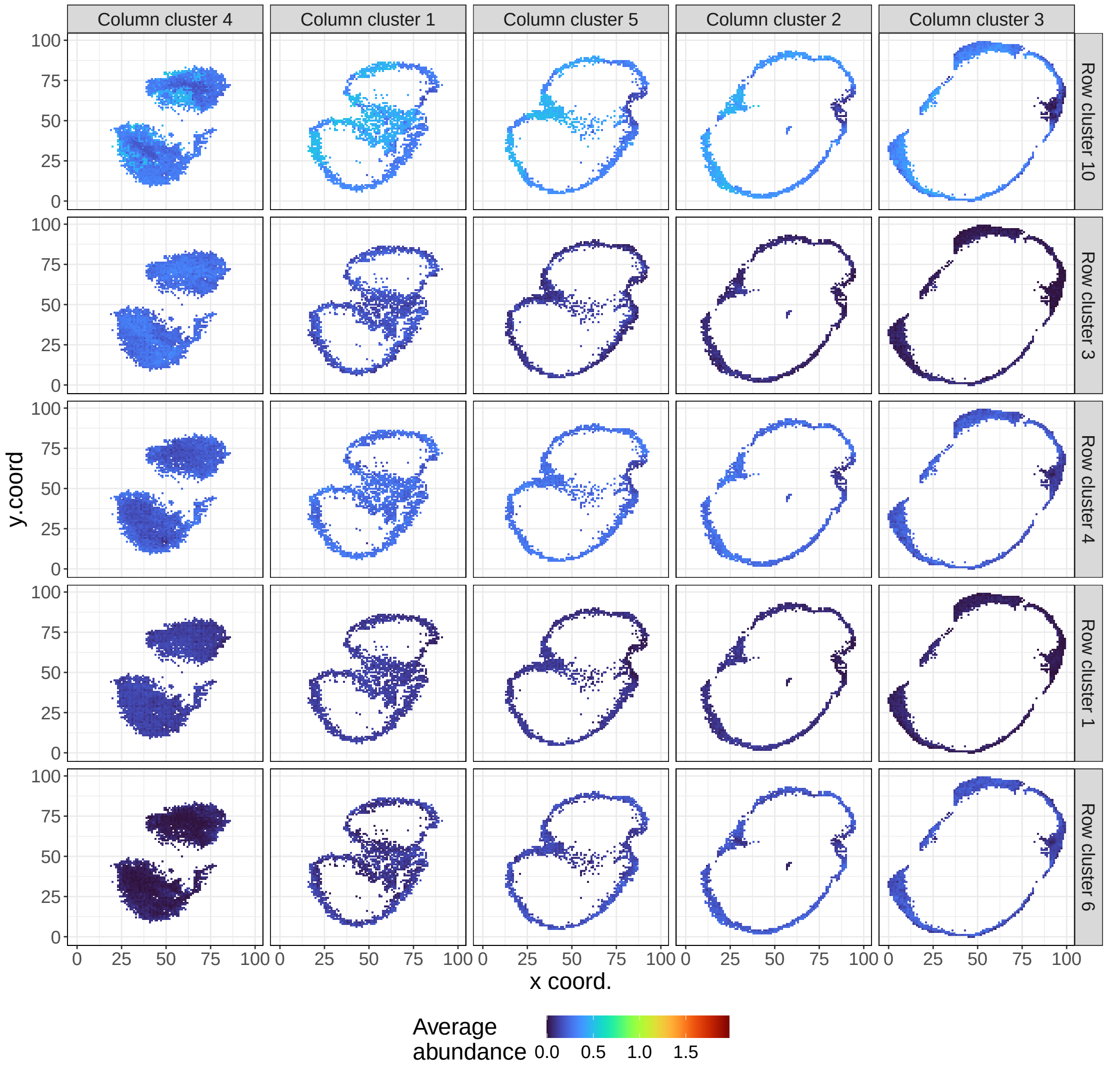

Figure 15: Continuation of Figure 14. The figure represents the second group of five protein clusters.
